# The More the Better—Investigation of Polymethoxylated *N*-Carboranyl Quinazolines as Novel Hybrid Breast Cancer Resistance Protein Inhibitors

**DOI:** 10.3390/pharmaceutics15010241

**Published:** 2023-01-10

**Authors:** Philipp Stockmann, Lydia Kuhnert, Wencke Leinung, Cathleen Lakoma, Birte Scholz, Svetlana Paskas, Sanja Mijatović, Danijela Maksimović-Ivanić, Walther Honscha, Evamarie Hey-Hawkins

**Affiliations:** 1 Institute of Inorganic Chemistry, Faculty of Chemistry and Mineralogy, Universität Leipzig, Johannisallee 29, 04103 Leipzig, Germany; 2 Institute of Pharmacology, Pharmacy and Toxicology, Faculty of Veterinary Medicine, Universität Leipzig, An den Tierkliniken 15, 04103 Leipzig, Germany; 3 Department of Immunology, Institute for Biological Research “Siniša Stanković”, Belgrade University, 11060 Belgrade, Serbia

**Keywords:** multidrug resistance, drug discovery, synthesis, structure-activity relationship, in silico modeling, carborane, ABCG2, breast cancer resistance protein

## Abstract

The ineffectiveness and failing of chemotherapeutic treatments are often associated with multidrug resistance (MDR). MDR is primarily linked to the overexpression of ATP-binding cassette (ABC) transporter proteins in cancer cells. ABCG2 (ATP-binding cassette subfamily G member 2, also known as the breast cancer resistance protein (BCRP)) mediates MDR by an increased drug efflux from the cancer cells. Therefore, the inhibition of ABCG2 activity during chemotherapy ought to improve the efficacy of the administered anti-cancer agents by reversing MDR or by enhancing the agents’ pharmacokinetic properties. Significant efforts have been made to develop novel, powerful, selective, and non-toxic inhibitors of BCRP. However, thus far the clinical relevance of BCRP-selective MDR-reversal has been unsuccessful, due to either adverse drug reactions or significant toxicities in vivo. We here report a facile access towards carboranyl quinazoline-based inhibitors of ABCG2. We determined the influence of different methoxy-substitution patterns on the 2-phenylquinazoline scaffold in combination with the beneficial properties of an incorporated inorganic carborane moiety. A series of eight compounds was synthesized and their inhibitory effect on the ABCG2-mediated Hoechst transport was evaluated. Molecular docking studies were performed to better understand the structure-protein interactions of the novel inhibitors, exhibiting putative binding modes within the inner binding site. Further, the most potent, non-toxic compounds were investigated for their potential to reverse ABCG2-mediated mitoxantrone (MXN) resistance. Of these five evaluated compounds, *N*-(*closo*-1,7-dicarbadodecaboran(12)-9-yl)-6,7-dimethoxy-2-(3,4,5-trimethoxyphenyl)-quinazolin-4-amine (**DMQCd**) exhibited the strongest inhibitory effect towards ABCG2 in the lower nanomolar ranges. Additionally, **DMQCd** was able to reverse BCRP-mediated MDR, making it a promising candidate for further research on hybrid inorganic-organic compounds.

## 1. Introduction

The development and improvement of different chemotherapeutics for cancer treatment have been the main research topic of numerous groups worldwide. Due to the occurrence of multidrug resistance (MDR), the efficacy of chemotherapy is often decreased [1,2,3,4]. MDR was proven to exist either as an intrinsic property of some cancer cells, or, more frequently, it can be developed over time by the cells [5,6,7,8]. MDR is strongly correlated to the overexpression of ATP-binding cassette (ABC) transporters, yet other mechanisms such as altered drug metabolism, decreased drug uptake, or increased resistance to apoptosis, as well as drug compartmentalization, are known [9]. ABC transporters facilitate the active excretion of endogenous or xenobiotic substrates against the concentration gradient by ATP-hydrolysis [10]. The ABC transporter family contains 7 subfamilies with 48 different proteins which are expressed in the human body. Classified into subfamilies ABC A to ABC G, based on their structural similarity as well as their phylogenetic, ABC proteins are often associated with nutrient transport or expression in tissues with barrier function [11,12]. However, three of these transporters are expressed in cancer cells and specifically have been associated with MDR: P-glycoprotein (P-gp or ABCB1), multidrug resistance-associated protein 1 (MRP1 or ABCC1), and the breast cancer resistance protein (BCRP or ABCG2) [13]. These ABC transporters are able to eliminate xenobiotics from cells, including a broad range of chemotherapeutics [13]. This efflux of chemotherapeutics results in a reduced intracellular drug concentration to non-therapeutic levels, lowering the possibility of a sufficient anticancer therapy. In addition, the transporters are considered predictors for the adverse prognosis of metastasis, contributing to inefficient therapy progression as well as higher mortality [14,15,16]. The successful inhibition of these ABC transporters, such as ABCG2, could reverse MDR by disabling the drug efflux from the cancer cells. In addition to the possibility of reversing MDR, the successful inhibition of BCRP can be employed to improve the pharmacokinetic characteristics and, thus, the bioavailability of the given chemotherapeutics [17].

**Figure 1:**
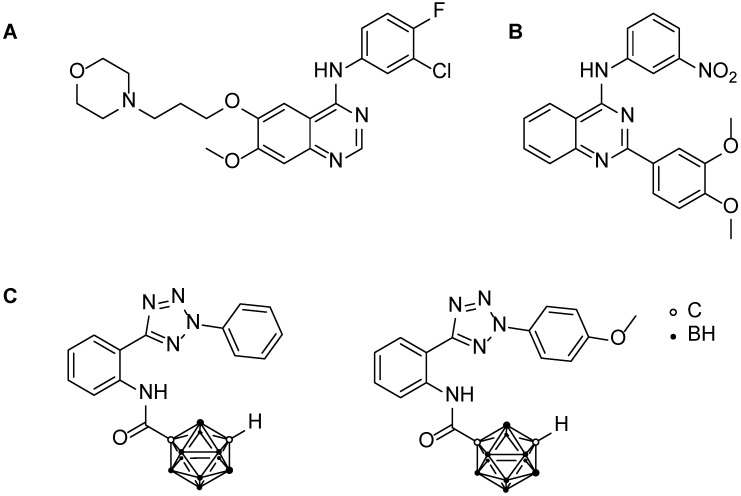
Molecular structures of known ABCG2 inhibitors of different classes. (**A**) Tyrosine kinase inhibitor gefitinib; (**B**) one of the strongest 2-phenyl-quinazoline-based ABCG2 inhibitors known [24]; (**C**) the first reported carborane-functionalized ABCG2 inhibitors [33].

So far, a variety of potential inhibitors for ABCG2 have been reported, constantly improving the efficacy, the selectivity, or the ability to act as pan-inhibitors for the three MDR-associated transporters [18,19,20]. However, creating an ideal inhibitor of BCRP is a challenging task. An optimal inhibitor comprises low cytotoxicity, good solubility, and biocompatibility, in combination with high inhibition of the target protein and the ability to reverse MDR [21]. One of the approaches towards developing an effective BCRP inhibitor is based on the class of tyrosine kinase inhibitors (TKIs). Molecules belonging to the TKI family have similar structural features, such as a quinazoline, quinoline, pyrimidine, or pyridine base [18]. In addition, some TKIs being used as chemotherapeutic agents, such as gefitinib or ceritinib (Figure 1), have been reported to act as substrates for ABCG2 [22,23]. This feature was initially used by Wiese and co-workers who designed and developed ABCG2 inhibitors based on quinazolines, and further assessed the structural requirements and the importance of substrate differences. They reported promising molecules showing the inhibition of BCRP in lower nanomolar ranges (Figure 1) [24,25,26,27,28,29], thus paving the way for future adaptation and research in the field of quinazoline-based molecules as optimal inhibitors of BCRP for the improvement of MDR-limited cancer therapies.

In recent years, a rather new, yet promising, field of research on organic-inorganic hybrid compounds evolved in the medicinal chemistry sphere. For us, one of the interesting incorporations of inorganic moieties into organic scaffolds has been the implementation of *closo*-dicarbadodecaboranes, known as carboranes (C_2_B_10_H_12_) [30]. Carboranes are three-dimensional boron-carbon clusters, recently being the focus of an emerging area of research in medicinal chemistry [31]. They are strongly hydrophobic clusters; implementing them into organic molecules provides a promising approach when dealing with, for example, the lipophilicity of cell membranes, as the clusters enhance the permeability or address hydrophobic binding sites within the target proteins. Further, utilizing the inorganic nature of the hybrid structures appears to be beneficial for the improvement of the drugs’ metabolic stability [32].

Köhler et al. previously reported the implementation of a carborane cluster as a functional group in BCRP inhibitors [33]. However, the two tested compounds (see Figure 1C) exhibited lower activity than their organic counterparts. Nevertheless, combining the favorable properties of a carborane cluster (hydrophobicity and 3D appearance) with a suitable organic scaffold could be useful for the interaction with the ABCG2 protein, leading to specific inhibition and, thus, a successful reversal of multidrug resistance.

Herein, we report the synthesis of eight *N*-carboranyl quinazoline derivatives, based on either a quinazoline or 6,7-dimethoxyquinazoline scaffold. Various methoxy substitution patterns of the quinazoline scaffold were studied to determine the substituents’ effect on the inhibition efficacy towards BCRP. Furthermore, in vitro and in silico experiments were conducted to test the potential of these molecules as BCRP inhibitors. In silico studies serve as an important starting point in understanding the interactions of the *N*-carboranyl quinazoline derivatives with the binding site of the ABCG2 protein. The experiments revealed these compounds to be prominent ABCG2 inhibitors with good levels of cytotoxicity showing signs of MDR reversal, therefore making them promising candidates for further research on hybrid inorganic-organic compounds. 

## 2. Materials and Methods

### 2.1. Synthetic Procedures and Analytical Data

All solvents were degassed, dried, and purified with the solvent purification system SPS-800 by MBraun and stored over activated 4 Å molecular sieves. All reactions were carried out under a nitrogen or argon atmosphere using standard Schlenk techniques and anhydrous, degassed solvents, unless otherwise stated. The deuterated solvent (CDCl_3_) was dried over P_2_O_5_, vacuum-transferred, and degassed by freeze-pump-thaw cycling. SPhos Pd G4 and *closo*-9-bromo-1,7-dicarbadodecaborane(12) (**CbBr**) were prepared according to the literature [34,35]. All other starting materials and reagents are commercially available and were used without further purification. The NMR spectra were recorded on a BRUKER Avance III HD 400 MHz NMR spectrometer at 25 °C (^1^H 400.13 MHz, ^11^B 128.38 MHz, ^13^C 100.63 MHz, two-dimensional (^1^H–^1^H COSY, ^1^H–^13^C HSQC, ^1^H–^13^C HMBC)). The chemical shifts δ are reported in parts per million (ppm). Tetramethylsilane (TMS) or solvent residual peaks were used as the internal reference in ^1^H and ^13^C NMR spectra, and all other nuclei spectra were referenced to TMS using the Ξ scale [36]. The numbering scheme for ^1^H and ^13^C NMR signals is presented for each compound in the SI. Electrospray ionization mass spectrometry was carried out with an ESI-qTOF Impact II by Bruker Daltonics GmbH in the positive mode. The IR spectra were obtained with an FT-IR spectrometer Nicolet iS5 (ATR, transmission, Thermo Scientific, Waltham, MA, USA) scanning between 4000–400 cm^−1^ with a KBr beam splitter (only selected frequencies given). Elemental analyses (C, H, and N) were performed with a Heraeus VARIO EL microanalyzer. The melting points were determined in glass capillaries using a Gallenkamp apparatus and are uncorrected. The column chromatography was performed using a Biotage Isolera ONE and KP-SIL columns.

Detailed chemical synthesis procedures and chemical analysis results of all compounds (**Qa-d**, **DMQa-d**, **QCa-d**, and **DMQCa-d**) are described in Appendix A and the Supplementary Materials.

### 2.2. Biological Methods

#### 2.2.1. Cultivation of MDCKII Cells

The MDCKII-hABCG2 and MDCKII-WT cells were purchased from Alfred Schinkel (Het Nederlands Kanker Instituut, Amsterdam, The Netherlands) and were cultivated in MEM (minimal essential medium) with Earle’s Salts (2.2 g/L NaHCO_3_, stable glutamine; Biowest, Nuaillé, France) supplemented with 10% (*v*/*v*) fetal calf serum (Life Technology, Karlsruhe, Germany), 1% (*v*/*v*) non-essential amino acids (Biowest, Nuaillé, France), 100 U/mL penicillin, and 100 µg/mL streptomycin (Biowest, Nuaillé, France). The cells were grown in a humidified atmosphere (37 °C, 5% CO_2_) and were sub-cultured every 3 to 4 days using 0.05% trypsin/0.02% EDTA (Biowest, Nuaillé, France), up to a total of 14 passages.

#### 2.2.2. Determination of Cell Viability by WST-1 Cell Proliferation Assay

MDCKII-hABCG2 cells and their parental MDCKII-WT cells were seeded in 96-well plates (TPP, Trasadingen, Switzerland) in a density of 2 × 10^4^ cells/mL and 3 × 10^4^ cells/mL, respectively. After 48 h, the cells were incubated with increasing concentrations of up to 50 µM of the novel synthesized compounds, with 0.1% Triton X-100 as the positive control and solvent (0.1% DMSO) as the negative control for 48 h. Afterwards, the substance-specific cytotoxicity was determined by the water-soluble tetrazolium-1 (4-[3-(4-iodophenyl)-2-(4-nitro-phenyl)-2*H*-5-tetrazolio]-1,3-benzene sulfonate; WST-1) assay as previously described [37]. The cell viability was determined by microplate reader at 450 nm (Tecan Sunrise, Crailsheim, Germany). The data of three independent experiments (N = 3) were tested for normality with the Shapiro-Wilk test. The significant differences compared to solvent control were analyzed by one-way ANOVA with the Holm-Šidák post hoc test using SigmaPlot 14.5 (Systec Software, San Jose, CA, USA). The data were normalized against the vehicle control (0.1% DMSO) and were expressed as mean ± SEM.

#### 2.2.3. Determination of ABCG2 Interaction with Hoechst 33342 Accumulation Assay

The Hoechst 33342 accumulation assay was used to detect an interaction of the investigated compounds with the human ABCG2 transporter. The assay protocol was adapted from that published by Wassermann et al. [38]. The MDCKII-hABCG2 (2 × 10^4^ cells/mL) and MDCKII-WT (3 × 10^4^ cells/mL) cells were seeded in 96-well plates and cultured for 72 h. Afterwards, the sub-confluent monolayers were treated with 0.5 µM and 1.0 µM of the investigational compounds or its respective solvent control (0.1% DMSO) for 4 h. The known ABCG2 inhibitor Ko143 (1 µM) was added as positive control. After 5 and 20 min, the cells were washed twice with ice-cold PBS buffer solution and lyzed with 100 µL/well 0.1% (*v*/*v*) SDS/PBS. The intracellular Hoechst 33342 amount was detected by spectrofluorometry (360 nm excitation/465 nm emission wavelengths, Tecan Infinite F200 Pro, Crailsheim, Germany). The examined fluorescence subtracted by the background of non-treated cells was correlated to the protein amount quantified by the bicinchoninic acid assay (BCA, Thermo Scientific, Rockford, IL, USA) following the manufacturer’s instructions. Relative fluorescence units (RFU) were calculated as the relation of fluorescence/mg protein of MDCKII-hABCG2 cells to MDCKII-WT cells, normalized to the solvent control set as 1 and expressed as mean ± SEM. The data from the Hoechst 3342 accumulation assay (five independent experiments N = 5) were tested for normality with the Shapiro-Wilk test. Significant differences compared to the solvent control were analyzed by one-way ANOVA with the Holm-Šidák post hoc test using SigmaPlot 14.5 (Systec Software, San Jose, CA, USA). An inhibition of human ABCG2 efflux transport activity is defined as the significant difference to the solvent control (0.1% DMSO).

#### 2.2.4. Determination of Autofluorescence

The autofluorescence of all compounds was investigated with a modified Hoechst 33342 accumulation assay. The MDCKII-hABCG2 and MDCKII-WT cells were seeded and treated as previously described for the Hoechst accumulation assay. Afterwards, the cells were washed twice with ice-cold PBS buffer, lyzed with 100 µL/well 0.1% (*v*/*v*) SDS/PBS, and, subsequently, the intracellular fluorescence was measured by spectrofluorometer (360 nm excitation/465 nm emission wavelengths, Tecan Infinite F200 Pro, Crailsheim, Germany). The protein amounts were quantified by BCA assay. For this approach, the Hoechst 33342 dye was not added. The background of non-treated cells was subtracted from the measured intracellular fluorescence and was correlated to the protein amount for MDCKII-hABCG2 or MDCKII-WT cells, respectively. All data were expressed as mean ± SEM and tested for normality by the Shapiro Wilk test using SigmaPlot 14.5. An autofluorescence was defined as the significant increase in total intracellular relative fluorescence unit (RFU) in comparison to the solvent-treated control and was determined by one-way ANOVA with Holm-Šidák post hoc test using SigmaPlot 14.5. As the negative control, the ABCG2-Inhibitor Ko143 (1 µM) was added.

#### 2.2.5. Reversal of Multidrug Resistance

The MDCKII cells were seeded as described in the WST-1 assay section, and treated with increasing concentrations of mitoxantrone (0.01 µM up to 50 µM) or solvent (0.1% DMSO) for 48 h. In order to detect a reversal of the ABCG2-mediated chemoresistance, novel ABCG2 interacting compounds (**QCc**, **DMQCa**, **DMQCb**, **DMQCc**, and **DMQCd**) were added to mitoxantrone in 1.0 µM for 48 h. Afterwards, the WST-1 assay was performed as described [37]. The left-shift factor was calculated from IC_50_ minus SEM from the mitoxantrone alone treatment by IC_50_ plus SEM from combined use of mitoxantrone and carborane-based derivatives (**QCc**, **DMQCa**, **DMQCb**, **DMQCc**, and **DMQCd**). Data were tested for normality by the Shapiro Wilk test and significant differences between the mitoxantrone treatment groups were determined by two-way ANOVA with the Holm-Šidák post hoc test using SigmaPlot 14.5. The IC_50_ values were defined as 50% reduced cell viability and calculated with SigmaPlot 14.5 by nonlinear regression. All data were normalized against the vehicle control and were expressed as mean ± SEM, calculated from at least three independent experiments.

### 2.3. Molecular Docking/Computational Methods

The structures were built with open-source molecular builder Avogadro 1.1.1 [39] and geometries were further optimized with ORCA 4.2.1, using the PBEh-3c method [40]. The electrostatic potential derived CHELPG charges were obtained from the ORCA-internal orca_chelpg program. The cryo-EM crystal structures of the whole human ABCG2 transporter protein (PDB code 5NJ3) and the MXN-ABCG2 co-crystallized protein-substrate adduct (PDB code 6VXI) were obtained from the Protein Data Bank (http://www.rcsb.org (accessed on 26 April 2021)) [41,42,43]. The UCSF Chimera package was used to remove all ligands, non-standard residues, and water molecules from the crystal structures [44]. The AutoDock Tools 1.5.6 (ADT) was used to prepare the structures for docking [45]. The protein and ligand structures were constructed for docking using a previously described process [46]. To comply to the AutoDock atom types, the missing hydrogen atoms were added to the protein structures, all polar hydrogen atoms were preserved, and all non-polar hydrogen atoms were merged. All histidine, lysine, and arginine residues were set to their protonated state, whereas all the aspartic acid and glutamic acid residues were deprotonated. For the protein, Gasteiger charges were applied to each atom. The above-described optimized geometries were used for docking. All torsion angles inside the carborane clusters were adjusted to non-rotatable, and the non-polar hydrogen atoms of the ligand were combined to match the AutoDock atom types. The docking was performed with AutoDock 4.2.6 according to a previously reported protocol (100 docking runs) [45,46]. The docking grid box dimension was set to 120 × 120 × 120 grid points along the *x*-, *y*-, and *z*-axes, respectively. The grid spacing of 0.375 Å was utilized. The grid center coordinates were 124.747, 124.778, and 143.202 (x, y, and z assignments, respectively). In order to group the final docked conformations, a tolerance of 1.5 Å root-mean-square deviations (RMSD) was used. The ADT 1.5.6 was used for setting up the docking protocols and result analysis. The AutoDock free energy scoring function had a standard error of 2–3 kcal/mol [45]. The UCSF ChimeraX software was used to visualize and render all molecular docking figures [44].

## 3. Results and Discussion

### 3.1. Chemistry

**Scheme 1:**
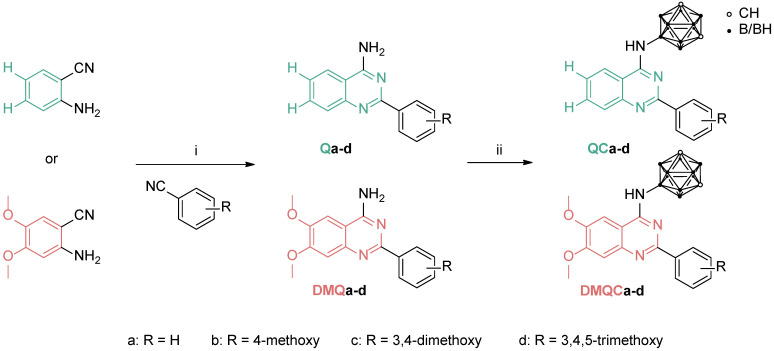
General synthesis of *N*-carboranyl 2-phenylquinazolin-4-amine derivatives (Q = quinazoline scaffold, green; DMQ = 6,7-dimethoxyquinazoline, red). Reagents and conditions: (i) (1) NaH, THF, reflux, 15−24 h; (2) HCl/H_2_O (ii) *closo*-9-Br-1,7-dicarbadodecaborane (**CbBr**), SPhos Pd G4, SPhos, KO^t^Bu, 1,4-dioxane, 80 °C, 1–2 h.

For the design and synthesis of BCRP inhibitors, quinazoline has previously been demonstrated as a potentially interesting structural motif. Many classes of quinazoline-based molecules have been recently described as effective and selective inhibitors [24,25,26,27,28,29]. The promising scaffold originates from tyrosine kinase inhibitors, such as gefitinib, that exhibited ABCG2 inhibition [22]. Eight substituted *N*-carboranyl 2-phenylquinazolin-4-amine derivatives were obtained via a simple two-step synthetic route. First, the 2-phenylquinazoline amines **Qa-d** and **DMQa-d** (Q = quinazoline scaffold, green; DMQ = 6,7-dimethoxyquinazoline scaffold, red; Scheme 1) were prepared following a protocol published by van Muijlwijk-Koezen et al. [47]. Sodium hydride, as a strong base, was used to deprotonate 2-aminobenzonitrile, forming a stable anion as an intermediate which further reacts with substituted benzonitriles. After an acid-catalyzed hydrolysis, quinazolines **Qad** and **DMQa-d** were obtained in moderate to good yields (43–80%). Finally, the desired *N*-carboranyl derivatives **QCa-d** and **DMQCa-d** were prepared in a modified fashion after Dziedzic et al., using *closo*-9-Br-1,7-dicarbadodecaborane (**CbBr**) with SPhos Pd G4 as a pre-catalyst in a Buchwald-Hartwig-like reaction, obtaining the final compounds in high yields (92−99%) [34,35]. All synthesized compounds were characterized by ^1^H NMR, ^11^B NMR (for carboranyl derivatives), ^13^C NMR and 2D NMR spectroscopy (see Supplementary Materials, Figures S1–S55), IR spectroscopy, and HR-MS, and the purity of the target compounds was confirmed by elemental analysis.

### 3.2. Biological Investigations

The eight target compounds (**QCa-d** and **DMQCa-d**) were evaluated for their cytotoxicity and ability to inhibit the ABCG2 transporter using the Madin-Darby canine kidney cells (MDCKII wild-type and MDCKII human ABCG2-transfected). In addition, a cell proliferation assay was used to investigate whether the most potent and promising ABCG2 inhibitors were able to reverse ABCG2-mediated mitoxantrone (MXN) resistance in the MDCKII-hABCG2 cells.

#### 3.2.1. Determination of Cytotoxicity in MDCKII-hABCG2 and MDCKII Wild-Type Cells

The cytotoxicity of the eight *N*-carboranyl quinazoline amines towards human ABCG2-transfected MDCKII cells and their parental MDCKII-WT cells were studied using a WST-1 proliferation assay. Triton X-100 and solvent (0.1% DMSO) were used as the positive and negative control, respectively. Substance-specific IC_50_ values (defined as 50% reduced cell viability) could only be calculated for unsubstituted (**QCa**, Figure S56A, Supplementary Materials) and mono-substituted (**QCb**, Figure S56B, Supplementary Materials) derivatives, with about 45 µM in MDCKII-WT cells (Table 1). The MDCKII-hABCG2 cells treated with the highest concentrations of 50 µM led to 30% cell viability, thus the IC_50_ values could only be given as higher than 10 µM. Due to limited solubility, only stock solutions of 50 mM (**DMQCb**: 25 mM) were used and, thus, no further IC_50_ values could be determined for the target compounds. A decreased cell viability of about 40% was detected for **QCc** (Figure S56C, Supplementary Materials) in MDCKII-hABCG2 cells and 30% for **DMQCb** (Figure S57B) in MDCKII-WT cells. All other compounds did not significantly affect the viability of MDCKII cells up to 50 µM (Figures S56 and S57, Supplementary Materials). The comparison of the toxicities between wild-type and transfected cells revealed no significant difference (Figures S56 and S57, Supplementary Materials), suggesting that the potential inhibitors themselves have a low toxicity. The results indicated that cell viability improves with an increasing degree of substitution. Especially, a higher level of substitution at the 2-phenyl moiety appears to be beneficial (Figure S56, Supplementary Materials). Additional methoxy substituents in the quinazoline structure (**DMQCa**, **DMQCb**, **DMQCc**, **DMQCd**, (Figure S57, Supplementary Materials) caused a reduced cytotoxicity compared with the unsubstituted quinazoline derivatives (**QCa**, **QCb**, **QCc**, **QCd**, Figure S56, Supplementary Materials).

#### 3.2.2. Hoechst 33342 Assay-Based Evaluation of the Inhibitory Potential toward MDCKII-hABCG2 Cells

**Figure 2:**
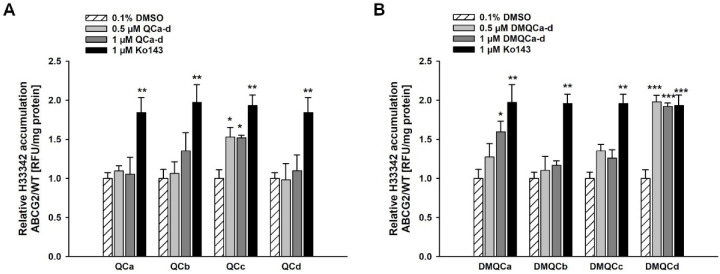
Intracellular Hoechst 33342 accumulation in MDCKII-hABCG2 cells in comparison to MDCKII-WT cells. Cells were treated with (**A**) **QCa**, **QCb**, **QCc**, **QCd**, or (**B**) **DMQCa**, **DMQCb**, **DMQCc**, **DMQCd**, or the positive control Ko143. Data were normalized to solvent control (0.1% DMSO) and are presented as mean ± SEM of five independent experiments (N = 5, one-way ANOVA with Holm-Šidák post hoc test, * significant difference in comparison to solvent control: *** *p* ≤ 0.001, ** *p* ≤ 0.01, * *p* ≤ 0.05).

The Hoechst 33342 accumulation assay was used to evaluate the efficacy of compounds **QCa-d** and **DMQCa-d** towards human ABCG2. Since the fluorescent dye Hoechst 33342 (2′-(4-ethoxyphenyl)-5-(4-methyl-1-piperazinyl)-2,5′-bi-1*H*-benzimidazol) is known to be a substrate of ABCG2, it is actively transported out of the cell. The inhibition of the ABCG2 transporter by the investigated compounds leads to an accumulation of the dye and, thus, increased intracellular fluorescence. The commercially available BCRP inhibitor Ko143 [(3*S*,6*S*,12a*S*)-1,2,3,4,6,7,12,12a-octahydro-9-methoxy-6-(2-methylpropyl)-1,4-dioxopyrazino[1′,2′:1,6]pyrido[3,4-*b*]indole-3-propanoic acid 1,1-dimethylethyl ester] was used as reference. The carboranyl quinazoline derivatives were applied in concentrations of 0.5 and 1.0 µM to determine the inhibition of the human ABCG2 transporter in low- and sub-micromolar ranges (Figure 2). These concentrations were selected due to our previous study showing that concentrations of 0.5 µM Ko143 exhibited lower intracellular Hoechst 33342 amounts than 1 µM. To screen whether the investigated compounds are able to inhibit in nanomolar ranges, 0.5 µM of each selected compound was chosen for a first screening approach. While the positive control Ko143 doubled the intracellular dye amount, the parental compound **QCa** and the methoxy-substituted derivatives **QCb** and **QCd** did not affect the human ABCG2-mediated Hoechst efflux (Figure 2A). Thus, among the unsubstituted quinazoline-scaffold structures (**QCa-d**), the 2-(3,4-dimethoxyphenyl) quinazoline derivative (**QCc**) was the only compound exhibiting inhibition within the tested concentration range. The presence of a third methoxy group at the phenyl moiety (**QCd**) unexpectedly diminished the efficacy towards the human ABCG2 in the Hoechst 33342 accumulation assay (Figure 2A). Juvale et al. investigated similar scaffolds and observed a beneficial effect of the 3,4-dimethoxyphenyl moiety in the 2-position for their compounds [24]. In the second group of compounds (**DMQCa-d**, Figure 2B), methoxy groups were introduced in the 6- and 7-position of the quinazoline moiety. For **DMQCa**, an improved inhibitory potency at 1.0 µM was observed, compared to its parental compound **QCa**. Interestingly, compound **DMQCd**, bearing five methoxy groups, was by far superior to all other tested compounds, showing the strongest inhibition at 0.5 µM with a comparable level as the reference Ko143. Unexpectedly, **DMQCb** and **DMQCc** caused only marginal increases in Hoechst 33342 accumulation (Figure 2B). However, high background values were observed, indicating a potential autofluorescence of **DMQCb** and **DMQCc**, which may have disturbed the fluorescence measurements, leading to an underestimation of ABCG2 interaction. Therefore, this phenomenon was further investigated.

#### 3.2.3. Determination of Autofluorescence

As indicated by the obtained data of the Hoechst accumulation assay, **DMQCb** and **DMQCc** seemed to exhibit autofluorescence. Therefore, all investigated compounds were tested for autofluorescence. To detect intracellular autofluorescence, the Hoechst assay was performed in a modified protocol without the Hoechst 33342 dye. After 4 h treatment, the medium was removed, the cells were washed twice, and the buffer solution was entirely removed before the lysis buffer was added. In contrast to the Hoechst 33342 accumulation assay, the Hoechst 33342 dye was not added and, thus, the detected fluorescence is solely intracellular, and an influence of other compounds can be excluded by comparison with the background and the solvent control. As shown in Figure S58 (Supplementary Materials), **QCb**, **QCd**, **DMQCa**, **DMQCb**, **DMQCc**, and **DMQCd** showed a dose-dependent significant increase in the total intracellular fluorescence in comparison with the solvent control. In addition, a treatment with 1 µM of **QCc** enhanced the intracellular fluorescence in a significant manner. The reference Ko143 emitted no fluorescence itself. This is in line with the data obtained after 5 min in the Hoechst accumulation assay, indicating an increased fluorescence for MDCKII-hABCG2 cells especially treated with **DMQCb** and **DMQCc**. The autofluorescence enhanced the background significantly, preventing the determination of hABCG2-mediated Hoechst accumulation. These results proved that **QCb**, **QCc**, **QCd**, **DMQCa**, **DMQCb**, **DMQCc**, or **DMQCd** are accumulating in MDCKII cells. However, further studies are needed to validate whether these compounds are ABCG2 inhibitors.

#### 3.2.4. Reversal of ABCG2-Mediated MDR

**Figure 3:**
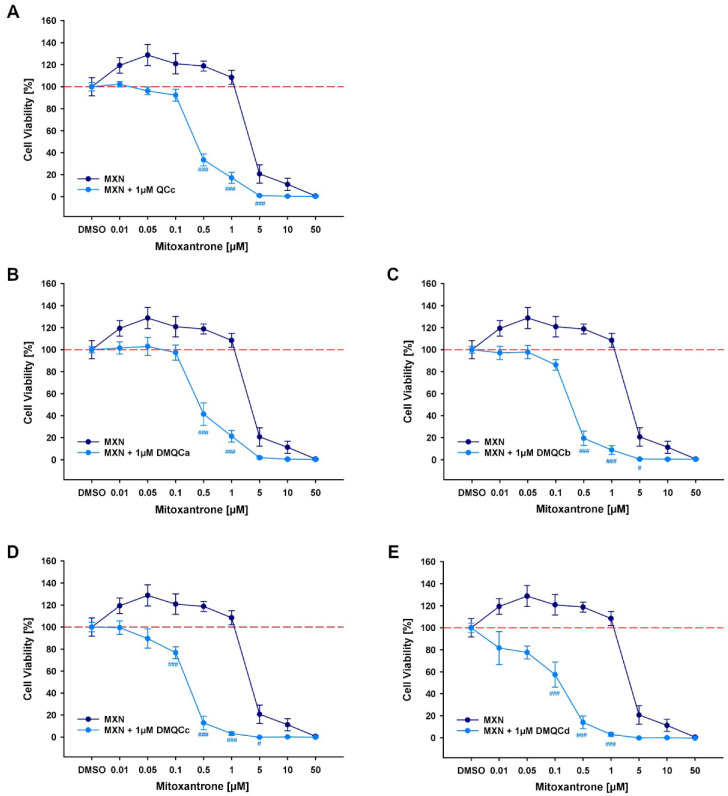
Reversal of ABCG2-mediated mitoxantrone (MXN) resistance of MDCKII-hABCG2 cells by 1 µM of (**A**) **QCc**, (**B**) **DMQCa**, (**C**) **DMQCb**, (**D**) **DMQCc**, and (**E**) **DMQCd**. MDCKII cells were treated with increasing concentrations of mitoxantrone with or without selected compounds for 48 h. Afterwards, cell viability was measured by WST-1 assay. Data are shown as mean ± SEM from three independent experiments (N = 3, two-way ANOVA with Holm-Šidák post hoc test, # significant difference in comparison to MXN treatment alone. ### *p* ≤ 0.001, # *p* ≤ 0.05, significant differences towards solvent control were not shown).

The expression of ABCG2 efflux transporters in several tumors, such as human melanoma and colon cancer, caused chemoresistance and poor 5-year survival rates [4,48,49]. Therefore, it was of interest to investigate whether the quinazoline derivatives reverse the hABCG2-mediated mitoxantrone (MXN) resistance in MDCKII cells. The hABCG2 inhibitors identified in the Hoechst assay (**QCc**, **DMQCa**, **DMQCd**, Figure 3A,B,E) and the compounds exhibiting the highest level of autofluorescence (**DMQCb**, **DMQCc**, Figure 3C,D) were selected for testing. MXN, as a known substrate for the human ABCG2 transporter, is considerably stronger excreted in the hABCG2-overexpressing MDCKII cell lines, compared with their parental cell line (MDCKII-WT). We have shown that the hABCG2-expressing cell line (MDCKII-hABCG2) exhibited a significantly higher MXN-specific IC_50_ value (2.649 ± 0.594 µM) than observed for MDCKII-WT (0.519 ± 0.042 µM). These results underline the hABCG2-mediated decreased toxicity of MXN, which is reversed by 1 µM of the reference compound Ko143 (0.295 ± 0.063 µM). As presented in Figure 3, the combined treatment of MXN with the hABCG2-inhibiting compounds (1 µM) significantly left-shifted the IC_50_ value (Table 2). The strongest left shift (11.6-fold) of MXN-specific IC_50_ values was obtained for **DMQCd** (Figure 3D). In comparison to the IC_50_ value of the combined treatment with Ko143 (1 µM; IC_50_: 0.295 ± 0.063 µM; left-shift factor: 5.7), the addition of **DMQCd** (Figure 3E) further reduced the IC_50_ value to half the value (0.144 ± 0.033 µM). This is in agreement with the data from the Hoechst 33342 accumulation assay, with **DMQCd** exhibiting the strongest ABCG2 inhibition among all tested compounds (Figure 2). Consequently, **QCc** and **DMQa-d** were able to reverse MXN resistance by inhibition of the human ABCG2 transporter in MDCKII cells.

### 3.3. In Silico Investigations—Molecular Docking Simulations and Investigation of Mode of Inhibition

Structure-based molecular docking, even though a rather ‘rough’ approach, represents a rational and useful in silico method to better understand binding modes and interactions between a designed compound and its biological target. As several carboranyl quinazoline derivatives showed cell assay-based inhibition for the ABCG2 transporter protein, we carried out docking simulations in comparison with Hoechst 33342 (in accordance with the literature [50,51,52,53]) and mitoxantrone (MXN, substrate of ABCG2) on the cryo-electron microscopy (cryo-EM) structures of human ABCG2 (PDB code 5NJ3) and human ABCG2 bound to MXN (PDB code 6VXI) [42,43]. Docking simulations of Hoechst and MXN were performed to assess the binding behavior of the carboranyl quinazoline derivatives. Binding of the molecules in the same manner at the binding site could thus indicate competitive inhibition between the inhibitor and substrate. The binding free energy values of all carboranyl derivatives **DMQCa-d** are given in Table S1 (Supplementary Materials).

**Figure 4:**
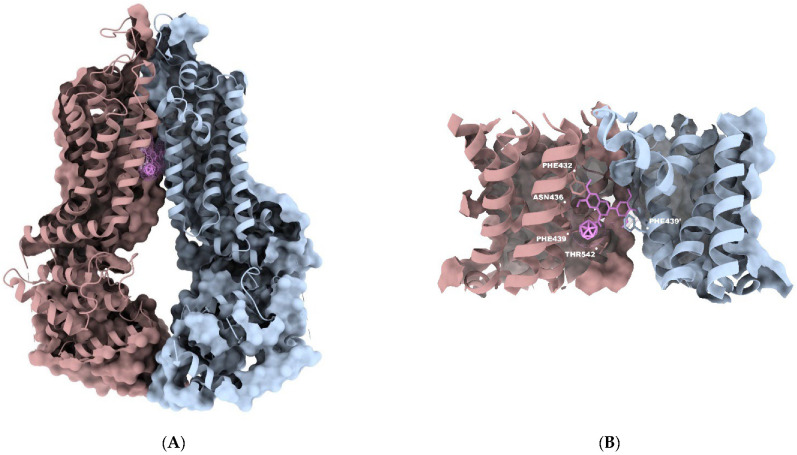

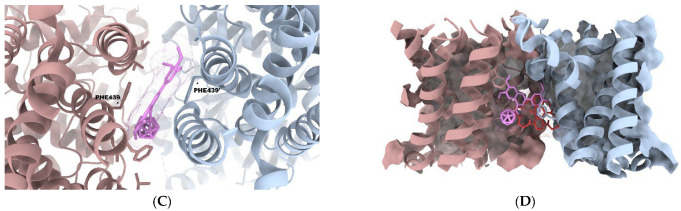
(**A**) Cartoon representation of ABCG2. Monomers are depicted in pink and blue. **DMQCd** bound to inner inhibitor-binding cavity (referred to as cavity S1) [26]. (**B**) Top-ranked pose of **DMQCd** docked into the ABCG2 cryo-EM structure (PDB code 5NJ3); **DMQCd** shown as stick model; hydrogen atoms omitted for clarity; binding free energy: −8.1 kcal/mol. (**C**) Docking of **DMQCd** into the crystal structure of ABCG2 (5NJ3); top-view, showing the sandwich-like binding mode between the Phe-439 residues of the protein. (**D**) Putative binding modes of top-ranked poses of **DMQCd** (pink) and MXN (red).

The predicted binding modalities of the carboranyl quinazoline-based structures showed similar orientations and interactions towards the transporter protein. Exemplarily, the best scoring binding pose of compound **DMQCd** (−8.1 kcal/mol) is shown in Figure 4A,B, as **DMQCd** exhibited the most promising results in the biological investigations. The predicted simulations were found foremost in the slit-like gap between the ABCG2 monomers and bound mostly by hydrophobic interaction—surrounded by hydrophobic amino acids, including Leu-405, Phe-432, Thr-542, Ile-543, and Val-546. Furthermore, π-π stacking was observed in a sandwich-like manner for the aromatic rings between the phenyl moiety of Phe-439 from the opposing monomers (Figure 4C). A detailed 2D interaction diagram of **DMQCd** and the protein side chains is presented in the Supplementary Materials (Figure S59). This is in agreement with the cryo-EM structures of the small molecule inhibitors in complex with ABCG2 [54]. In total, 98% of the top 200 docking poses of **DMQCd** discovered from molecular docking experiments using the AutoDock 4.2.6 were occupying the inner binding site, generally referred to as inhibitor binding site S1 [26,45]. In general, all quinazoline derivatives exhibited S1-occupying docking poses in >95% of the top 100 docking poses. However, depending on the substitution pattern of the quinazoline scaffold, we observed deviations in their orientations. The carborane moiety of the dimethoxyquinazoline scaffolds (**DMQCa-d**) protruded into the inner cavity, suggesting a ‘blocking’-like fashion of the molecule inside the binding site, whereas derivatives with an unsubstituted quinazoline scaffold (**QCa-d**) showed an upward facing (into the extracellular space) orientation of the three-dimensional moiety. 

Additionally, the most abundant predicted docking modes of all examined structures overlaid with the top-ranked poses of MXN in the alleged binding cavity of ABCG2 (Figure 4D). This further agrees with the co-crystallized structure of MXN bound to BCRP (PDB code 6VXI) and the literature [55]. The obtained data indicate that the inhibition of the transporter protein is possibly caused by the competition of the inhibitor and substrate.

## 4. Conclusions

The ability of carboranyl 2-phenylquinazoline derivatives to inhibit the human ABCG2 transporter protein and thus reversing ABCG2-mediated multidrug resistance was studied. The 2-phenylquinazoline scaffold has been reported before as the main structure for strong inhibitors. However, despite the ability for strong inhibition, inconsistencies often persist among the inhibitor’s intrinsic toxicity and metabolic stability. Therefore, the inorganic three-dimensional carborane moiety was introduced as a non-toxic and metabolically stable pharmacophore. Furthermore, the influence of different methoxy-substitution patterns on the 2-phenylquinazoline scaffold on the inhibitory potency towards the human ABCG2 transporter was investigated.

We discovered five potent compounds (**QCc**, **DMQCa**, **DMQCb**, **DMQCc**, and **DMQCd**), exhibiting low cytotoxicity in wild-type and human ABCG2-transfected MDCKII cells, and inhibiting the human ABCG2 transporter at 1 µM. In addition, **QCc** and **DMQCd** inhibited the human ABCG2 transporter in Hoechst 33342 accumulation assays in sub-micromolar concentrations (0.5 µM). Among the examined compounds, **DMQCd**, bearing the highest number of methoxy groups of the tested quinazoline derivatives, showed the strongest inhibition of the transporter protein in both tested concentrations comparable to the reference Ko143 (1 µM). **DMQCb** and **DMQCc** showed no significant inhibition of the human ABCG2 transporter in a Hoechst 33342 accumulation assay, which was explained and confirmed by autofluorescence of both compounds. Therefore, both derivatives were also included in further assessments. As three possible binding sites within the BCRP are known, molecular docking studies were carried out on the cryo-EM structure of ABCG2 in comparison with the Hoechst 33342 and the antineoplastic agent mitoxantrone, in order to understand the mode of inhibition. The computational methods revealed a strong binding of the carboranyl quinazoline derivatives within the inner, slit-like cavity S1 of the protein. The putative binding modes of the examined structures furthermore overlapped with the binding poses of MXN. This suggests a competitive inhibition behavior between the inhibitor and the substrate. Eventually, the most potent compounds were investigated for reversal of ABCG2-mediated MDR in a WST-1-based cytotoxicity assay by co-administration of the chemotherapeutic agent MXN. All five investigated compounds reversed the MXN resistance by inhibiting human ABCG2 efflux activity. Prominently, the compounds **DMQCc** and **DMQCd** showed by far the strongest ABCG2 inhibition with very low cytotoxicity and, strikingly, the ability to reverse ABCG2-mediated resistance in combination with the ABCG2 substrate and antineoplastic agent MXN. **DMQCd** was able to reverse MDR, causing an 11.6-fold left shift of the MXN-specific IC_50_ value, which was even stronger than detected for the reference inhibitor Ko143.

In summary, combining the chemical, computational, and biological methods shown here provides a promising approach for further development of BCRP inhibitors. We have demonstrated that the incorporation of carboranes as a three-dimensional pharmacophore has beneficial effects on the targeted inhibition and reversion of ABCG2-mediated MDR, making these carboranyl quinazoline derivatives promising candidates for further studies.

## Figures and Tables

**Table 1:** Cytotoxicity of compounds **QCa-d** and **DMQCa-d** determined by WST-1 cell proliferation assay using MDCKII wild-type (MDCKII-WT) and human ABCG2-transfected MDCKII cells (MDCKII-hABCG2). Data given as mean ± SEM of three independent experiments (N = 3).

Compound	R	IC_50_ ± SEM [µM] MDCKII-WT	IC_50_ ± SEM [µM] MDCKII-hABCG2
**QCa**	H	43.230 ± 0.159	>10
**QCb**	4-methoxy	44.167 ± 0.174	>10
**QCc**	3,4-dimethoxy	>50	>50
**QCd**	3,4,5-trimethoxy	>50	>50
**DMQCa**	H	>50	>50
**DMQCb**	4-methoxy	>25 *	>25 *
**DMQCc**	3,4-dimethoxy	>50	>50
**DMQCd**	3,4,5-trimethoxy	>50	>50

* Highest applied concentration 25 µM, due to poor solubility of **DMQCb** in DMSO.

**Table 2:** Left-shift factor calculated as IC_50_ MXN/IC_50_ MXN in combination with quinazoline derivatives determined in MDCKII-hABCG2 cells using WST-1 assay. IC_50_ values are given as mean ± SEM, N = 3, two-way ANOVA, * represents significant difference in comparison to single MXN treatment, (*** *p* < 0.001).

Treatment of MDCKII-hABCG2	IC_50_ [µM]	Left-Shift Factor	Comparison to MXN *
MXN	2.649 ± 0.594		
MXN + 1 µM **QCc**	0.346 ± 0.033	5.4-fold	***
MXN + 1 µM **DMQCa**	0.426 ± 0.057	4.3-fold	***
MXN + 1 µM **DMQCb**	0.254 ± 0.031	7.2-fold	***
MXN + 1 µM **DMQCc**	0.184 ± 0.025	9.8-fold	***
MXN + 1 µM **DMQCd**	0.144 ± 0.033	11.6-fold	***

## Data Availability

Not applicable.

## Supplementary Materials

The following supporting information can be downloaded at: https://www.mdpi.com/article/10.3390/pharmaceutics15010241/s1; 1. NMR spectra of quinazoline-4-amines **Qa-d** and **DMQa-d** (Figures S1–S15); 2. NMR spectra of *N*-carboranyl quinazoline-4-amines **QCa-d** and **DMQCa-d** (Figures S16–S55); 3. Biological Data (Figures S56–S58); 4. Molecular Docking (Figure S59, Table S1).

## Abbreviations

ABC, ATP-binding cassette; ATP, adenosine-triphosphate; BCRP, breast cancer resistance protein; cryo-EM, cryogenic electron microscopy; IC50, half-maximal inhibitory concentration; MDCK, Madin-Darby canine kidney; MDR, multidrug resistance; MRP1, multidrug resistance-associated protein 1; P-gp, P-glycoprotein; SEM, standard error of the mean; TKI, tyrosine kinase inhibitor; WST, water-soluble tetrazolium dye.

## Appendix A

A. General procedure for preparation of quinazolin-4-amines

In an oven-dried single-neck flask, the sodium hydride (1.2 eq., 60% dispersion in mineral oil) was dissolved in dry THF (5 mL/mmol). The corresponding 2-aminobenzonitrile (1.0 eq.) was dissolved in dry THF (2 mL/mmol) and added dropwise at 0 °C. After warming to room temperature, the nitrile (2.0 eq.) was added dropwise or in small portions. The reaction mixture was heated under reflux for 15−24 h, or until complete conversion (TLC-monitored). After cooling to room temperature, the distilled water (5 mL/mmol) and conc. HCl_aq_ (1.0 eq.) were added, and the organic solvents were removed under reduced pressure. Water (10 mL/mmol) and EtOAc (25 mL/mmol) were added, and the pH was adjusted to 7–8 using 1.5 M NaOH solution. After phase separation, the aqueous phase was further extracted with EtOAc (2 × 30 mL/mmol). The combined organic phases were washed with saturated brine and dried over MgSO_4_. The crude product was purified by column chromatography, using *n*-hexane/EtOAc as eluents on silica gel.

A.1 The 2-phenylquinazolin-4-amine (**Qa**) was obtained from 2-aminobenzonitrile (4.20 mmol) and benzonitrile as a colorless solid in 59% yield (557 mg) according to the general procedure described above. R_f_ = 0.52 (*n*-hexane/EtOAc, 1:1, *v*/*v*). ^1^H NMR (400 MHz, CDCl_3_): δ [ppm] = 8.51–8.49 (m, 2H), 7.97–7.95 (m, 1H), 7.80–7.73 (m, 2H), 7.52–7.44 (m, 4H), 5.71 (s, 2H). HR-MS (ESI(+), acetonitrile): *m*/*z* calc. [C_14_H_12_N_3_] ([M+H]^+^): 222.1031, found: 222.1034.

A.2 The 2-(4-methoxyphenyl)quinazolin-4-amine (**Qb**) was obtained from 2-aminobenzonitrile (1.50 mmol) and 4-methoxybenzonitrile as a colorless solid in 43% yield (163 mg) according to the general procedure described above. R_f_ = 0.37 (*n*-hexane/EtOAc, 1:1, *v*/*v*). ^1^H NMR (400 MHz, CDCl_3_): δ [ppm] = 8.48–8.45 (m, 2H), 7.92 (d, *J* = 8.3 Hz, 1H), 7.77–7.71 (m, 2H), 7.44–7.40 (m, 1H), 7.01–6.98 (m, 2H), 5.66 (s, 2H), 3.88 (s, 3H). ^13^C{^1^H} NMR (101 MHz, CDCl_3_): δ [ppm] = 161.61, 161.49, 160.68, 151.18, 133.29, 131.26, 130.08, 128.69, 125.43, 121.76, 113.82, 112.96, 55.47. HR-MS (ESI(+), acetonitrile): *m*/*z* calc. [C_15_H_14_N_3_O] ([M+H]^+^): 252.1131, found: 252.1131.

A.3 The 2-(3,4-dimethoxyphenyl)quinazolin-4-amine (**Qc**) was obtained from 2-aminobenzonitrile (4.23 mmol) and 3,4-dimethoxybenzonitrile as a beige solid in 65% yield (779 mg) according to the general procedure described above. R_f_ = 0.21 (*n*-hexane/EtOAc, 1:1, *v*/*v*). ^1^H NMR (400 MHz, CDCl_3_): δ [ppm] = 8.13 (dd, *J* = 8.4, 2.0 Hz, 1H), 8.11 (d, *J* = 2.0 Hz, 1H), 7.93 (d, *J* = 8.6 Hz, 1H), 7.76–7.71 (m, 2H), 7.42–7.38 (m, 1H), 6.96 (d, *J* = 8.4 Hz, 1H), 5.79 (s, 2H), 4.02 (s, 3H), 3.92 (s, 3H). ^13^C{^1^H} NMR (101 MHz, CDCl_3_): δ [ppm] = 161.48, 160.60, 151.22, 151.09, 148.90, 133.30, 131.53, 128.76, 125.49, 121.74, 113.00, 111.21, 110.79, 56.07. HR-MS (ESI(+), methanol): *m*/*z* calc. [C_16_H_16_N_3_O_2_] ([M+H]^+^): 282.1237, found: 282.1243.

A.4 The 2-(3,4,5-trimethoxyphenyl)quinazolin-4-amine (**Qd**) was obtained from 2-aminobenzonitrile (4.23 mmol) and 3,4,5-trimethoxybenzonitrile as a beige solid in 80% yield (1050 mg) according to the general procedure described above. R_f_ = 0.24 (*n*-hexane/EtOAc, 1:1, *v*/*v*). ^1^H NMR (400 MHz, CDCl_3_): δ [ppm] = 7.94 (d, *J* = 8.3 Hz, 1H), 7.82 (s, 2H), 7.76–7.72 (m, 2H), 7.42–7.38 (m, 1H), 5.72 (s, 2H), 3.96 (s, 6H), 3.91 (s, 3H). ^13^C{^1^H} NMR (101 MHz, CDCl_3_): δ [ppm] = 161.59, 160.33, 153.19, 150.98, 140.05, 134.10, 133.37, 128.68, 125.77, 121.85, 113.03, 105.59, 77.48, 61.01, 56.26. HR-MS (ESI(+), methanol): *m*/*z* calc. [C_17_H_18_N_3_O_3_] ([M+H]^+^): 312.1343, found: 312.1345.

A.5 The 6,7-dimethoxy-2-phenylquinazolin-4-amine (**DMQa**) was obtained from 4,5-dimethoxy-2-aminobenzonitrile (5.61 mmol) and benzonitrile as an off-white solid in 61% yield (970 mg) according to the general procedure described above. R_f_ = 0.22 (*n*-hexane/EtOAc, 1:1, *v*/*v*). ^1^H NMR (400 MHz, CDCl_3_): δ [ppm] = 8.38 (dd, *J* = 7.8, 1.8 Hz, 2H), 7.44–7.35 (m, 3H), 7.19 (d, *J* = 1.1 Hz, 1H), 6.86 (s, 1H), 5.32 (s, 2H), 3.98 (d, *J* = 1.1 Hz, 3H). ^13^C{^1^H} NMR (101 MHz, CDCl_3_): δ [ppm] = 160.18, 159.94, 155.15, 149.10, 148.55, 138.85, 129.94, 128.50, 128.14, 108.13, 107.05, 100.29, 56.42, 56.32. HR-MS (ESI(+), methanol): *m*/*z* calc. [C_16_H_16_N_3_O_2_] ([M+H]^+^): 282.1237, found: 282.1237.

A.6 The 6,7-dimethoxy-2-(4-methoxyphenyl)quinazolin-4-amine (**DMQb**) was obtained from 4,5-dimethoxy-2-aminobenzonitrile (3.00 mmol) and 4-methoxybenzonitrile as a pink solid in 69% yield (644 mg) according to the general procedure described above. R_f_ = 0.17 (*n*-hexane/EtOAc, 2:3, *v*/*v*). ^1^H NMR (400 MHz, CDCl_3_): δ [ppm] = 8.42–8.40 (m, 2H), 7.28 (s, 1H), 7.00–6.98 (m, 2H), 6.91 (s, 1H). 5.43 (bs, 2H), 4.03 (s, 3H), 3.98 (s, 3H), 3.87 (s, 3H). ^13^C{^1^H} NMR (101 MHz, CDCl_3_): δ [ppm] = 161.34, 160.10, 159.71, 155.10, 148.79, 148.63, 131.51, 129.67, 113.81, 107.96, 106.77, 100.37, 56.40, 56.30, 55.48, 29.85. HR-MS (ESI(+), acetonitrile): *m*/*z* calc. [C_17_H_18_N_3_O_3_] ([M+H]^+^): 312.1343, found: 312.1343.

A.7 The 6,7-dimethoxy-2-(3,4-dimethoxyphenyl)quinazoline-4-amine (**DMQc**) was obtained from 4,5-dimethoxy-2-aminobenzonitrile (2.81 mmol) and 3,4-dimethoxybenzonitrile as a beige solid in 78% yield (746 mg) according to the general procedure described above. R_f_ = 0.21 (*n*-hexane/EtOAc, 1:4, *v*/*v*). ^1^H NMR (400 MHz, CDCl_3_): δ [ppm] = 8.10–8.07 (m, 2H), 7.30 (s, 1H), 6.96 (d, *J* = 8.4 Hz, 1H), 6.93 (s, 1H), 5.43 (s, 2H), 4.03 (d, *J* = 2.3 Hz, 6H), 3.98 (s, 3H), 3.95 (s, 3H). ^13^C{^1^H} NMR (101 MHz, CDCl_3_): δ [ppm] = 160.10, 159.57, 155.11, 150.82, 148.91, 148.84, 148.59, 131.73, 121.27, 110.92, 110.82, 107.96, 106.81, 100.38, 56.37, 56.29, 56.07. HR-MS (ESI(+), methanol): *m*/*z* calc. [C_18_H_20_N_3_O_4_] ([M+H]^+^): 342.1448, found: 342.1448.

A.8 The 6,7-dimethoxy-2-(3,4,5-trimethoxyphenyl)quinazolin-4-amine (**DMQd**) was obtained from 4,5-dimethoxy-2-aminobenzonitrile (1.68 mmol) and 3,4,5-trimethoxybenzonitrile as a beige solid in 55% yield (347 mg) according to the general procedure described above. R_f_ = 0.39 (*n*-hexane/EtOAc, 1:4, *v*/*v*). ^1^H NMR (400 MHz, CDCl_3_): δ [ppm] = 7.78 (s, 2H), 7.30 (s, 1H), 7.00 (s, 1H), 5.75 (bs, 2H), 4.00 (s, 3H), 3.97 (s, 6H), 3.93 (s, 3H), 3.90 (s, 3H). ^13^C{^1^H} NMR (101 MHz, CDCl_3_): δ [ppm] = 160.24, 159.28, 155.06, 153.18, 148.97, 148.37, 139.73, 134.37, 107.88, 106.91, 105.19, 100.50, 61.00, 56.28. HR-MS (ESI(+), methanol): *m*/*z* calc. [C_19_H_22_N_3_O_5_] ([M+H]^+^): 372.1554, found: 372.1555.

B. General procedure for preparation of *N*-carboranyl 2-phenylquinazolin-4-amines

In an oven-dried Schlenk flask, *closo*-9-bromo-1,7-dicarbadodecaborane(12) (1.00 mmol), quinazoline (1.10 mmol), SPhos (5 mol%), SPhos Pd G4 pre-catalyst (5 mol%), and potassium *tert*-butoxide (2.00 mmol) were placed. The flask was evacuated three times and backfilled with argon. The 1,4-dioxane (2 mL) was added and the mixture was placed in an 80 °C oil bath and stirred for 1–2 h until complete conversion (TLC-monitored). EtOAc was added, and the suspension was filtered over a plug of Celite^®^ and silica. After removal of solvents, the crude product was purified by column chromatography on silica gel (*n*-hexane/EtOAc).

B.1 The *N*-(*closo*-1,7-dicarbadodecaboran(12)-9-yl)-2-phenylquinazolin-4-amine (**QCa**) was obtained from 2-phenylquinazolin-4-amine as a beige solid in 97% yield (354 mg) according to the general procedure described above. R_f_ = 0.31 (*n*-hexane/EtOAc, 6:1, *v*/*v*). Mp. = 178–181 °C (pentane). ^1^H NMR (400 MHz, CDCl_3_): δ [ppm] = 8.62 (d, *J* = 7.1 Hz, 2H, H2′), 7.93 (d, *J* = 8.2 Hz, 1H, H8), 7.78–7.71 (m, 2H, H5, H7), 7.52–7.42 (m, 4H, H6, H3′, H4′), 5.51 (s, 1H, NH), 3.70–1.28 (br, 9H, cluster-BH), 2.94 (s, 2H, cluster-CH). ^13^C{^1^H} NMR (101 MHz, CDCl_3_): δ [ppm] = 161.69 (1C, C4), 160.84 (1C, C2), 151.12 (1C, C8a), 139.46 (1C, C2′), 132.57 (1C, C7), 129.96 (1C, C4′), 129.13 (1C, C8), 128.86 (2C, C3′), 128.29 (2C, C2′), 125.51 (1C, C5), 121.02 (1C, C6), 114.74 (1C, C4a), 51.64 (2C, cluster-C). ^11^B{^1^H} NMR (128 MHz, CDCl_3_): δ [ppm] = 0.4 (s, 1B, BN), −6.5 (s, 2B), −11.0 (s, 1B), −13.7 (s, 2B), −15.5 (s, 2B), −18.6 (s, 1B), −21.9 (s, 1B). ^11^B NMR (128 MHz, CDCl_3_): δ [ppm] = 0.4 (s, 1B, BN), −6.5 (d, 2B), −11.0 (d, 1B), −14.7 (t, 4B), −18.6 (d, 1B), −21.9 (d, 1B). HR-MS (ESI(+), acetonitrile): *m*/*z* calc. [C_17_H_22_B_10_N_3_] ([M+H]^+^): 364.2811, found: 364.2830. IR (KBr): ṽ [cm^−1^] = 3440, 3054, 2586, 1618, 1363, 1201, 751, 705. Elemental Analysis (C_16_H_21_B_10_N_3_) calc. (%): C 52.87, H 5.82, N 11.56 found (%): C 52.96, H 5.91, N 11.50.

B.2 The *N*-(*closo*-1,7-dicarbadodecaboran(12)-9-yl)-2-(4-methoxyphenyl)-quinazolin-4-amine (**QCb**) was obtained from 2-(4-methoxyphenyl)quinazolin-4-amine as a yellowish solid in 94% yield (369 mg) according to the general procedure described above. R_f_ = 0.31 (*n*-hexane/EtOAc, 3:1, *v*/*v*). Mp. = 151–154 °C (pentane). ^1^H NMR (400 MHz, CDCl_3_): δ [ppm] = 8.60 (d, *J* = 8.4 Hz, 2H, H2′), 7.91 (d, *J* = 8.3 Hz, 1H, H8), 7.76–7.68 (m, 2H, H5, H7), 7.39 (t, *J* = 7.5 Hz, 1H, H6), 7.03 (d, *J* = 8.4 Hz, 2H, H3′), 5.51 (s, 1H, NH), 3.89 (s, 3H, OCH_3_), 3.62–1.35 (br, 9H, cluster-BH), 2.94 (s, 2H, cluster-CH). ^13^C{^1^H} NMR (101 MHz, CDCl_3_): δ [ppm] = 161.52 (1C, C_q_), 161.36 (1C, C_q_), 160.53 (1C, C_q_), 151.04 (1C, C_q_), 132.50 (1C, C7), 132.08 (1C, C_q_), 130.39 (2C, C2′), 128.72 (1C, C8), 125.08 (1C, C6), 121.01 (1C, C5), 114.45 (1C, C4a), 113.61 (2C, C3′), 55.42 (1C, OCH_3_), 51.58 (2C, cluster-C). ^11^B{^1^H} NMR (128 MHz, CDCl_3_): δ [ppm] = 0.4 (s, 1B, BN), −6.5 (s, 2B), −11.0 (s, 1B), −13.8 (s, 2B), −15.5 (s, 2B), −18.7 (s, 1B), −21.9 (s, 1B). ^11^B NMR (128 MHz, CDCl_3_): δ [ppm] = 0.4 (s, 1B, BN), −6.5 (d, 2B), −11.1 (d, 1B), −14.7 (t, 4B), −18.7 (d, 1B), −22.6 (d, 1B). HR-MS (ESI(+), acetonitrile): *m*/*z* calc. [C_17_H_23_B_10_N_3_O] ([M+H]^+^): 394.2917, found: 394.2920. IR (KBr): ṽ [cm^−1^] = 3444, 3054, 2970, 2610, 1607, 1357, 1249, 834, 756. Elemental Analysis (C_17_H_23_B_10_N_3_O) calc. (%): C 51.89, H 5.89, N 10.68 found (%): C 51.99, H 5.98, N 10.70.

B.3 The *N*-(*closo*-1,7-dicarbadodecaboran(12)-9-yl)-2-(3,4-dimethoxyphenyl)-quinazolin-4-amine (**QCc**) was obtained from 2-(3,4-dimethoxyphenyl)quinazolin-4-amine as a light-yellow solid in 96% (407 mg) yield according to the general procedure described above. R_f_ = 0.59 (*n*-hexane/EtOAc, 1:1, *v*/*v*). Mp. = 285–287 °C (pentane). ^1^H NMR (400 MHz, CDCl_3_): δ [ppm] = 8.29 (d, *J* = 2.0 Hz, 1H, H2′), 8.26 (d, *J* = 8.7 Hz, 1H, H6′), 7.91 (d, *J* = 8.4 Hz, 1H, H8), 7.76 (d, *J* = 8.2 Hz, 1H, H5), 7.70 (t, *J* = 7.6 Hz, 1H, H7), 7.40 (t, *J* = 7.6 Hz, 1H, H6), 6.99 (d, *J* = 8.4 Hz, 1H, H5′), 5.54 (s, 1H, NH), 4.07 (s, 3H, OCH_3_), 3.96 (s, 3H, OCH_3_), 3.68–1.39 (br, 9H, cluster-BH), 2.93 (s, 2H, cluster-CH). ^13^C{^1^H} NMR (101 MHz, CDCl_3_): δ [ppm] = 161.37 (1C, C4), 160.19 (1C, C2), 150.66 (1C, C4′), 148.54 (1C, C3′), 132.46 (1C, C7), 132.05 (1C, C1′), 128.64 (1C, C8), 125.07 (1C, C6), 121.70 (1C, C6′), 120.91 (1C, C5), 114.34 (1C, C4a), 111.64 (1C, C2′), 110.6 (1C, C5′), 55.92 (2C, OCH_3_), 51.43 (2C, cluster-C). ^11^B{^1^H} NMR (128 MHz, CDCl_3_): δ [ppm] = 0.4 (s, 1B, BN), −6.4 (s, 2B), −11.1 (s, 1B), −13.8 (s, 2B), −15.4 (s, 2B) −18.7 (s, 1B), −22.0 (s, 1B). ^11^B NMR (128 MHz, CDCl_3_): δ [ppm] = 0.4 (s, 1B, BN), −6.4 (d, 2B), −11.1 (d, 1B), −14.7 (t, 4B), −18.7 (d, 1B), −21.8 (d, 1B). HR-MS (ESI(+), acetonitrile): *m*/*z* calc. [C_18_H_25_B_10_N_3_O_2_] ([M+H]^+^): 424.3023, found: 424.3024. IR (KBr): ṽ [cm^−1^] = 3382, 3060, 2932, 2591, 1557, 1369, 1214, 757, 719. Elemental Analysis (C_18_H_25_B_10_N_3_O_2_) calc. (%): C 51.05, H 5.95, N 9.92 found (%): C 51.18, H 6.03, N 9.89.

B.4 The *N*-(*closo*-1,7-dicarbadodecaboran(12)-9-yl)-2-(3,4,5-trimethoxyphenyl)-quinazolin-4-amine (**QCd**) was obtained from 2-(3,4,5-trimethoxyphenyl)quinazolin-4-amine as a light yellow solid in 95% yield (431 mg) according to the general procedure described above. R_f_ = 0.65 (*n*-hexane/EtOAc, 1:1, *v*/*v*). Mp. = 212–215 °C (pentane). ^1^H NMR (400 MHz, CDCl_3_): δ [ppm] = 8.00 (d, *J* = 1.4 Hz, 2H, H2′), 7.93 (d, *J* = 8.4 Hz, 1H, H8), 7.77 (d, *J* = 8.2 Hz, 1H, H5), 7.72 (t, *J* = 7.7 Hz, 1H, H7), 7.43 (t, *J* = 7.6 Hz, 1H, H6), 5.55 (s, 1H, NH), 4.04 (d, *J* = 1.4 Hz, 6H, OCH_3_), 3.93 (s, 3H, OCH_3_), 3.69–1.39 (br, 9H, cluster-BH), 2.92 (s, 2H, cluster-CH). ^13^C{^1^H} NMR (101 MHz, CDCl_3_): δ [ppm] = 161.51 (1C, C4), 160.03 (1C, C2), 153.09 (2C, C3′), 139.81 (1C, C4′), 134.70 (1C, C1′), 132.64 (1C, C7), 128.91 (1C, C8), 125.49 (1C, C6), 121.07 (1C, C5), 114.57 (1C, C4a), 105.81 (2C, C2′), 61.04 (1C, OCH_3_), 56.28 (2C, OCH_3_), 51.57 (2C, cluster-C). ^11^B{^1^H} NMR (128 MHz, CDCl_3_): δ [ppm] = 0.5 (s, 1B, BN), −6.2 (s, 2B), −11.1 (s, 1B), −14.6 (d, 4B), −18.6 (s, 1B), −21.9 (s, 1B). ^11^B NMR (128 MHz, CDCl_3_): δ [ppm] = 0.5 (s, 1B, BN), −6.3 (d, 2B), −11.1 (d, 1B), −14.6 (t, 4B), −18.7 (d, 1B), −21.7 (d, 1B). HR-MS (ESI(+), acetonitrile): *m*/*z* calc. [C_19_H_27_B_10_N_3_O_3_] ([M+H]^+^): 454.3128, found: 454.3131. IR (KBr): ṽ [cm^−1^] = 3363, 3053, 2940, 2605, 1554, 1355, 1221, 764, 725. Elemental Analysis (C_19_H_27_B_10_N_3_O_3_) calc. (%): C 50.32, H 6.00, N 9.26 found (%): C 50.48, H 6.09, N 9.20.

B.5 The *N*-(*closo*-1,7-dicarbadodecaboran(12)-9-yl)-6,7-dimethoxy-2-phenyl-quinazolin-4-amine (**DMQCa**) was obtained from 6,7-dimethoxy-2-phenylquinazolin-4-amine as an off-white solid in 99% yield (419 mg) according to the general procedure described above. R_f_ = 0.59 (*n*-hexane/EtOAc, 1:1, *v*/*v*). Mp. = 261–264 °C (pentane). ^1^H NMR (400 MHz, CDCl_3_): δ [ppm] = 8.59–8.56 (m, 2H, H2′), 7.50–7.41 (m, 3H, H3′, H4′), 7.31 (s, 1H, H8), 6.93 (s, 1H, H5), 5.17 (s, 1H, NH), 4.04 (s, 3H, OCH_3_), 4.04 (s, 3H, OCH_3_), 3.74–1.43 (br, 9H, cluster-BH), 2.94 (s, 2H, cluster-CH). ^13^C{^1^H} NMR (101 MHz, CDCl_3_): δ [ppm] = 160.31 (1C, C4), 159.68 (1C, C2), 154.45 (1C, C7), 148.88 (1C, C6), 148.22 (1C, C8a), 139.56 (1C, C1′), 129.62 (1C, C4′), 128.51 (2C, C2′), 128.26 (2C, C3′), 108.34 (2C, C4a, C8), 100.03 (1C, C5), 56.45 (1C, OCH_3_), 56.33 (1C, OCH_3_), 51.52 (2C, cluster-C). ^11^B{^1^H} NMR (128 MHz, CDCl_3_): δ [ppm] = 0.5 (s, 1B, BN), −6.6 (s, 2B), −11.2 (s, 1B), −13.8 (s, 2B), −15.6 (s, 2B), −18.7 (s, 1B), −22.1 (s, 1B). ^11^B NMR (128 MHz, CDCl_3_): δ [ppm] = 0.5 (s, 1B, BN), −6.6 (d, 2B), −11.2 (d, 1B), −14.8 (t, 4B), −18.7 (d, 1B), −22.6 (d, 1B). HR-MS (ESI(+), acetonitrile): *m*/*z* calc. [C_18_H_25_B_10_N_3_O_2_] ([M+H]^+^): 424.3023, found: 424.3030. IR (KBr): ṽ [cm^−1^] = 3440, 3059, 2929, 2599, 1617, 1363, 1211, 772, 708. Elemental Analysis (C_18_H_25_B_10_N_3_O_2_) calc. (%): C 51.05, H 5.95, N 9.92 found (%): C 51.07, H 6.04, N 9.80.

B.6 The *N*-(*closo*-1,7-dicarbadodecaboran(12)-9-yl)-6,7-dimethoxy-2-(4-methoxy-phenyl)quinazolin-4-amine (**DMQCb**) was obtained from 6,7-dimethoxy-2-(4-methoxyphenyl)quinazolin-4-amine as a beige solid in 98% yield (444 mg) according to the general procedure described above. R_f_ = 0.27 (*n*-hexane/EtOAc, 3:2, *v*/*v*). Mp. = 140–143 °C (CHCl_3_). ^1^H NMR (400 MHz, CDCl_3_): δ [ppm] = 8.54 (d, *J* = 8.5 Hz, 2H, H2′), 7.29 (s, 1H, H8), 7.04 (s, 1H, H5), 7.00 (d, *J* = 8.4 Hz, 2H, H3′), 5.42 (s, 1H, NH), 3.98 (s, 3H, OCH_3_), 3.94 (s, 3H, OCH_3_), 3.86 (s, 3H, OCH_3_), 3.56–1.43 (br, 9H, cluster-BH), 2.91 (s, 2H, cluster-CH). ^13^C{^1^H} NMR (101 MHz, CDCl_3_): δ [ppm] = 161.04 (1C, C_q_), 160.24 (1C, C_q_), 159.37 (1C, C_q_), 154.29 (1C, C_q_), 148.47 (1C, C_q_), 148.09 (1C, C_q_), 132.16 (1C, C_q_), 129.98 (2C, C2′), 113.53 (2C, C3′), 108.08 (1C, C4a), 107.93 (1C, C8), 100.22 (1C, C5), 56.26 (1C, OCH_3_), 56.18 (1C, OCH_3_), 55.35 (1C, OCH_3_), 51.41 (2C, cluster-C). ^11^B{^1^H} NMR (128 MHz, CDCl_3_): δ [ppm] = 0.5 (s, 1B, BN), −6.6 (s, 2B), −11.2 (s, 1B), −13.8 (s, 2B), −15.6 (s, 2B), −18.7 (s, 1B), −22.2 (s, 1B). ^11^B NMR (128 MHz, CDCl_3_): δ [ppm] = 0.5 (s, 1B, BN), −6.5 (d, 2B), −11.3 (d, 1B), −15.6 (t, 4B), −18.9 (d, 1B), −22.0 (d, 1B). HR-MS (ESI(+), acetonitrile): *m*/*z* calc. [C_19_H_27_B_10_N_3_O_3_] ([M+H]^+^): 454.3128, found: 454.3149. IR (KBr): ṽ [cm^−1^] = 3387, 3059, 2932, 2601, 1607, 1362, 1211, 792, 748. Elemental Analysis (C_19_H_27_B_10_N_3_O_3_) calc. (%): C 50.32, H 6.00, N 9.26 found (%): C 50.18, H 6.11, N 9.31.

B.7 The *N*-(*closo*-1,7-dicarbadodecaboran(12)-9-yl)-6,7-dimethoxy-2-(3,4-dimethoxy-phenyl)-quinazolin-4-amine (**DMQCc**) was obtained from 6,7-dimethoxy-2-(3,4-dimethoxyphenyl)quinazolin-4-amine as an off-white solid in 94% yield (455 mg) according to the general procedure described above. R_f_ = 0.23 (*n*-hexane/EtOAc, 1:1, *v*/*v*). Mp. = 147–150 °C (CHCl_3_). ^1^H NMR (400 MHz, CDCl_3_): δ [ppm] = 8.24 (d, *J* = 8.7 Hz, 2H, H2′, H6′), 7.46 (s, 1H, H8), 7.03 (s, 1H, H5), 6.98 (d, *J* = 8.4 Hz, 1H, H5′), 5.50 (s, 1H, NH), 4.06 (s, 3H, OCH_3_), 4.01 (d, *J* = 8.8 Hz, 6H, OCH_3_), 3.94 (s, 3H, OCH_3_), 3.54–1.50 (br, 9H, cluster-BH), 2.92 (s, 2H, cluster-CH). ^13^C{^1^H} NMR (101 MHz, CDCl_3_): δ [ppm] = 160.22 (1C, C4), 159.14 (1C, C2), 154.50 (1C, C7), 150.56 (1C, C_q_), 148.68 (3C, C_q_), 121.42 (1C, C6′), 111.51 (1C, C2′), 110.73 (1C, C5′), 108.06 (1C, C4a), 107.96 (1C, C8), 100.16 (1C, C5), 56.44 (1C, OCH_3_), 56.32 (1C, OCH_3_), 56.04 (2C, OCH_3_), 51.48 (2C, cluster-C). ^11^B{^1^H} NMR (128 MHz, CDCl_3_): δ [ppm] = 0.5 (s, 1B, BN), −6.4 (s, 2B), −11.2 (s, 1B), −14.0 (s, 2B), −15.5 (s, 2B), −18.8 (s, 1B), −22.3 (s, 1B). ^11^B NMR (128 MHz, CDCl_3_): δ [ppm] = 0.5 (s, 1B, BN), −6.4 (d, 2B), −11.1 (d, 1B), −14.7 (t, 2B), −18.6 (d, 1B), −21.8 (m, 1B). HR-MS (ESI(+), acetonitrile): *m*/*z* calc. [C_20_H_29_B_10_N_3_O_4_] ([M+H]^+^): 484.3234, found: 484.3235. IR (KBr): ṽ [cm^−1^] = 3381, 3054, 2923, 2600, 1562, 1364, 1208, 792, 718. Elemental Analysis (C_20_H_29_B_10_N_3_O_4_) calc. (%): C 49.67, H 6.04, N 8.69 found (%): C 49.77, H 6.13, N 8.59.

B.8 The *N*-(*closo*-1,7-dicarbadodecaboran(12)-9-yl)-6,7-dimethoxy-2-(3,4,5-trimethoxyphenyl)-quinazolin-4-amine (**DMQCd**) was obtained from 6,7-dimethoxy-2-(3,4,5-trimethoxyphenyl)quinazolin-4-amine as an off-white solid in 92% yield (472 mg) according to the general procedure described above. R_f_ = 0.29 (*n*-hexane/EtOAc, 1:1, *v*/*v*). Mp. = 162–163 °C (CHCl_3_). ^1^H NMR (400 MHz, CDCl_3_): δ [ppm] = 7.96 (s, 2H, H2′), 7.31 (s, 1H, H8), 6.95 (s, 1H, H5), 5.20 (s, 1H, NH), 4.04 (d, *J* = 2.7 Hz, 12H, OCH_3_), 3.92 (s, 3H, OCH_3_), 3.55–1.48 (br, 9H, cluster-BH), 2.92 (s, 2H, cluster-CH). ^13^C{^1^H} NMR (101 MHz, CDCl_3_): δ [ppm] = 160.15 (1C, C4), 158.97 (1C, C2), 154.48 (1C, C7), 153.09 (2C, C3′), 148.87 (1C, C6), 148.18 (1C, C8a) 139.50 (1C, C4′), 134.99 (1C, C1′), 108.29 (1C, C4a), 105.43 (2C, C2′, C8), 100.06 (1C, C5), 61.04 (1C, OCH_3_), 56.47 (1C, OCH_3_), 56.29 (1C, OCH_3_), 56.26 (2C, OCH_3_), 51.5 (2C, cluster-C). ^11^B{^1^H} NMR (128 MHz, CDCl_3_): δ [ppm] = 0.5 (s, 1B, BN), −6.4 (s, 2B), −11.4 (s, 1B), −14.8 (d, 4B), −18.8 (s, 1B), −22.3 (s, 1B). ^11^B NMR (128 MHz, CDCl_3_): δ [ppm] = 0.5 (s, 1B, BN), −6.4 (d, 2B), −11.3 (d, 1B), −14.8 (t, 4B), −18.8 (d, 1B), −22.1 (d, 1B). HR-MS (ESI(+), acetonitrile): *m*/*z* calc. [C_21_H_31_B_10_N_3_O_5_] ([M+H]^+^): 514.3340, found: 514.3353. IR (KBr): ṽ [cm^−1^] = 3384, 3054, 2928, 2600, 1562, 1362, 1208, 852, 823. Elemental Analysis (C_21_H_31_B_10_N_3_O_5_) calc. (%): C 49.11, H 6.08, N 8.18 found (%): C 49.17, H 6.18, N 8.13.
