# Supplementary material for: The More the Better—Investigation of Polymethoxylated N-Carboranyl Quinazolines as Novel Hybrid Breast Cancer Resistance Protein Inhibitors
Source: Pharmaceutics. 2023 Jan 10;15(1):241. doi: 10.3390/pharmaceutics15010241 (PMC9866861; doi:10.3390/pharmaceutics15010241)
Supplement: Supplementary file 1 [file pharmaceutics-15-00241-s001.zip › pharmaceutics-2107174-supplementary.pdf]

Supporting information to:

The More the Better – Investigation of Polymethoxylated *N*-Carboranyl Quinazolines as Novel Hybrid Breast Cancer Resistance Protein Inhibitors

Philipp Stockmann [1], Lydia Kuhnert [2,*], Wencke Leinung [1,†], Cathleen Lakoma [2], Birte Scholz [2], Svetlana Paskas [3], Sanja Mijatović [3], Danijela Maksimović-Ivanić [3], Walther Honscha [2] and Evamarie Hey-Hawkins [1,*]

[1] Institute of Inorganic Chemistry, Faculty of Chemistry and Mineralogy, Universität Leipzig, Johannisallee 29, 04103 Leipzig, Germany
[2] Institute of Pharmacology, Pharmacy and Toxicology, Faculty of Veterinary Medicine, Universität Leipzig, An den Tierkliniken 15, 04103 Leipzig, Germany
[3] Department of Immunology, Institute for Biological Research "Siniša Stanković", Belgrade University, 11060 Belgrade, Serbia
* Correspondence: lydia.kuhnert@vetmed.uni-leipzig.de (L.K.); hey@uni-leipzig.de (E.H.-H.)
† Current address: Max-Planck-Institut für Kohlenforschung, Kaiser-Wilhelm-Platz 1, 45470 Mülheim an der Ruhr, Germany.

This PDF file includes

1. NMR spectra of quinazoline-4-amines **Qa-d** and **DMQa-d**
(Figures S1 to S15)

2. NMR spectra of *N*-carboranyl quinazoline-4-amines **QCa-d** and **DMQCa-d**
(Figures S16 to S55)

3. Biological Data
(Figures S56 to S58)

4. Molecular Docking
(Figure S59, Table S1)

1. NMR spectra of quinazoline-4-amines Qa-d and DMQa-d

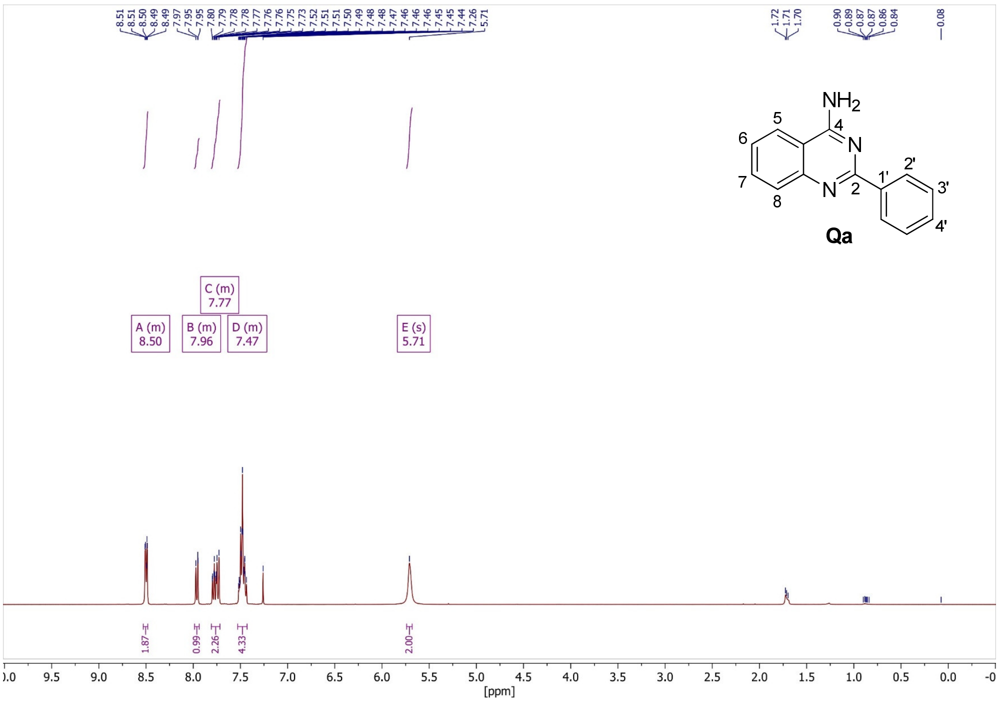

Figure S1. ^{1}H NMR spectrum of **Qa** in $CDCl_3$.

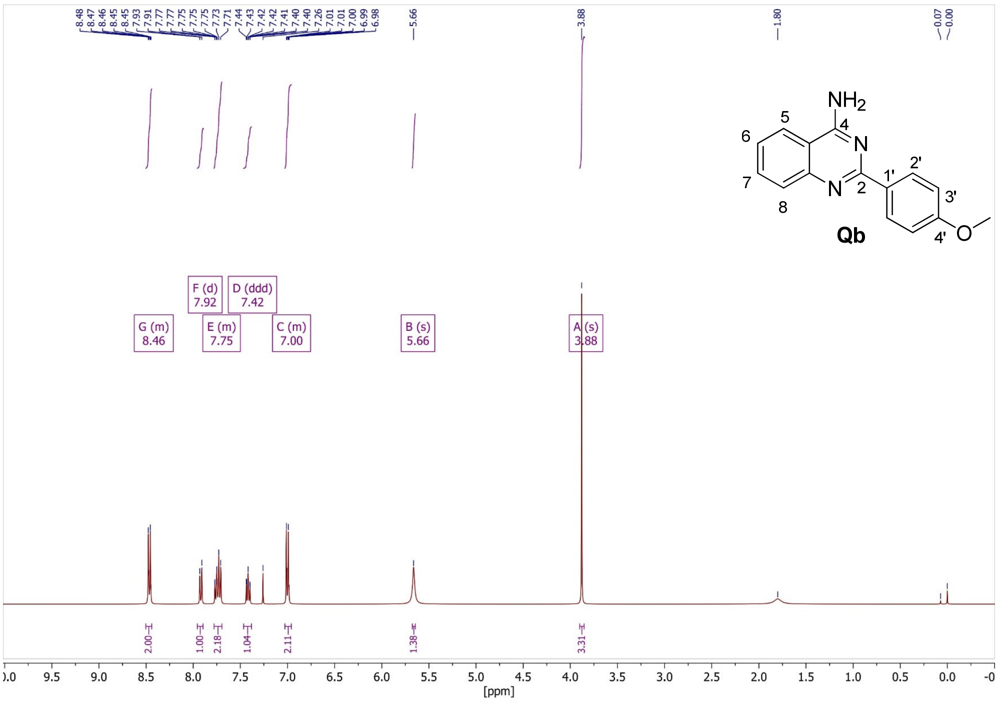

Figure S2. ^{1}H NMR spectrum of **Qb** in $CDCl_3$.

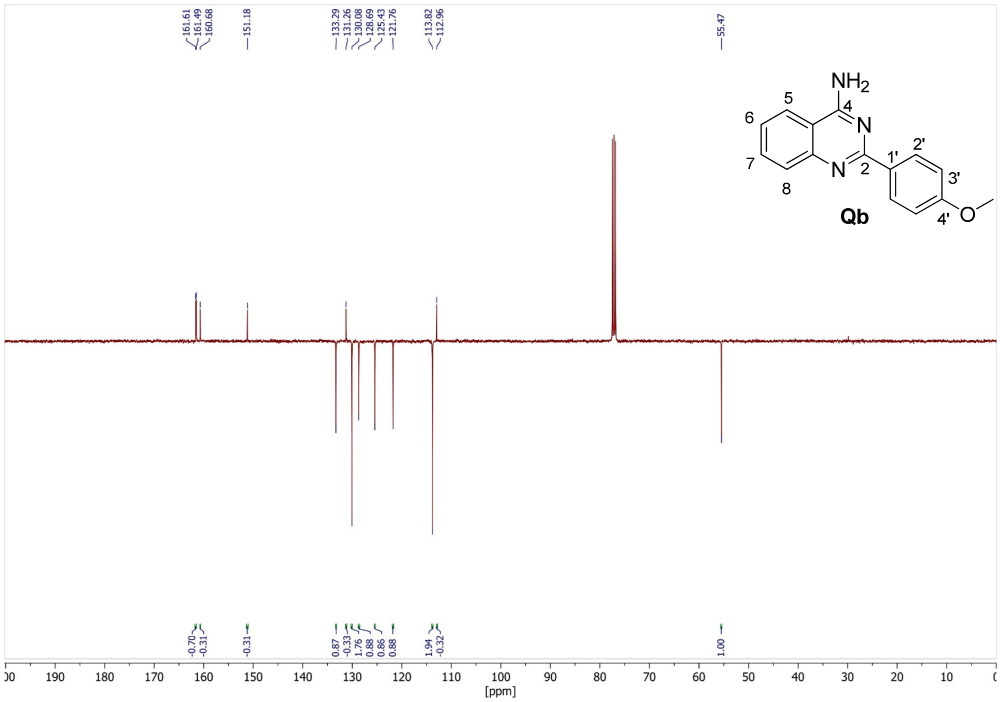

Figure S3. APT-$^{13}C\{^1H\}$ NMR spectrum of **Qb** in $CDCl_3$.

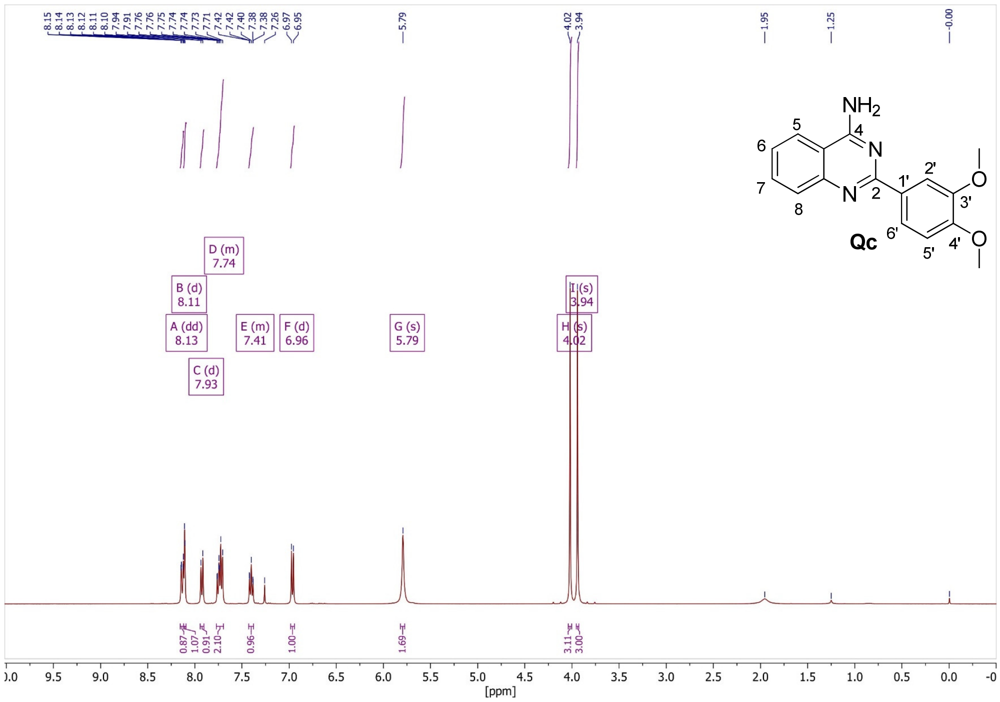

Figure S4. ^1H NMR spectrum of **Qc** in $CDCl_3$.

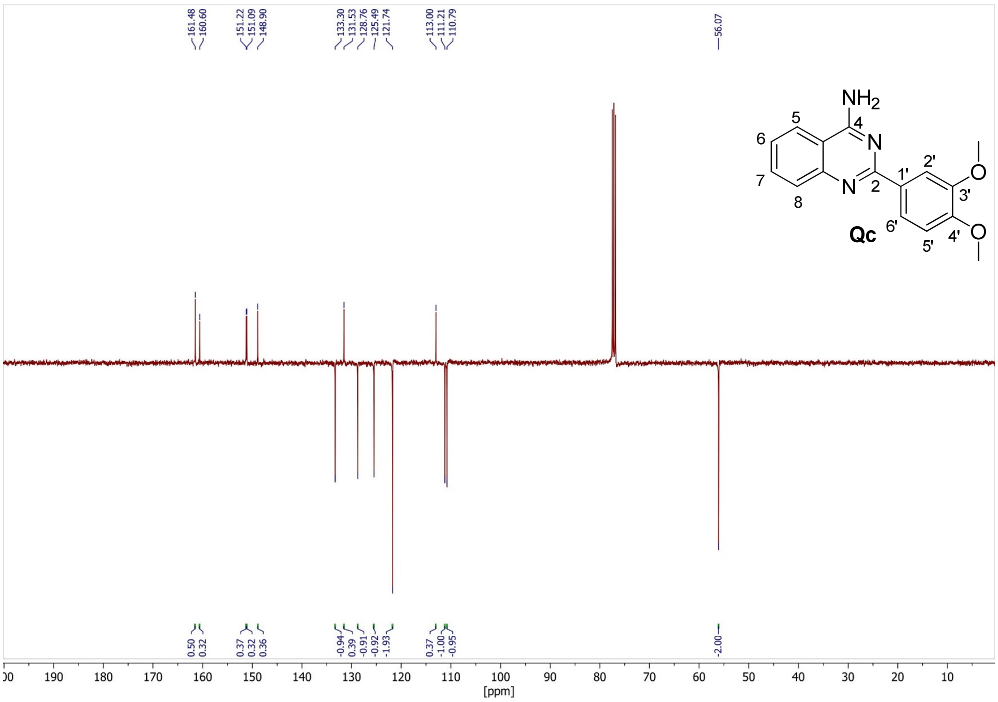

Figure S5. APT-$^{13}C\{^{1}H\}$ NMR spectrum of **Qc** in $CDCl_3$.

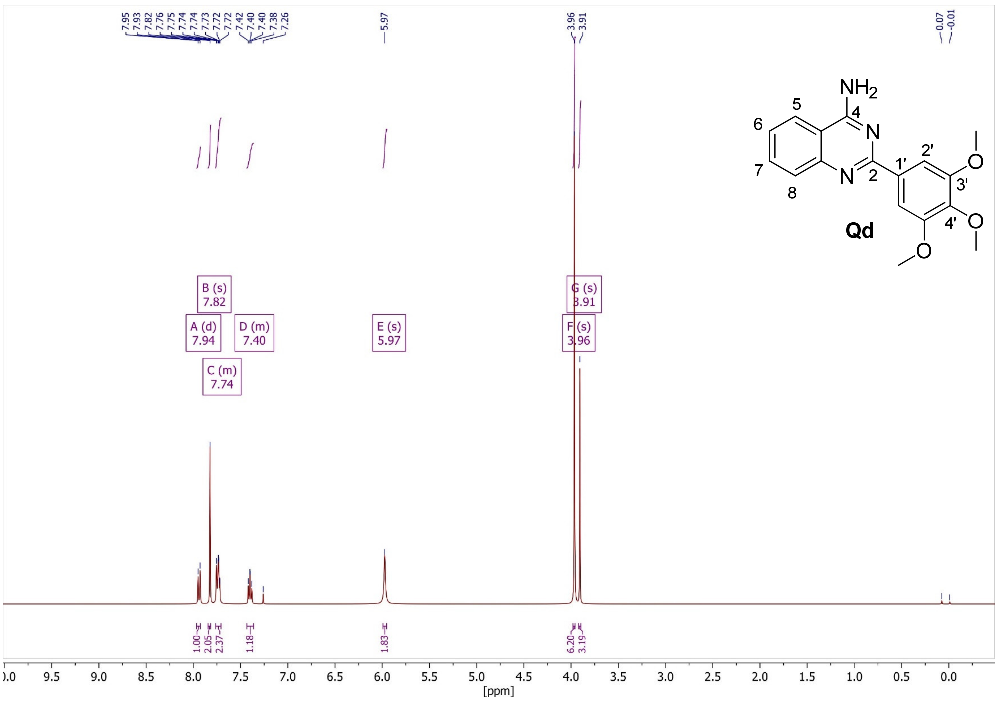

Figure S6. ^{1}H NMR spectrum of **Qd** in $CDCl_3$.

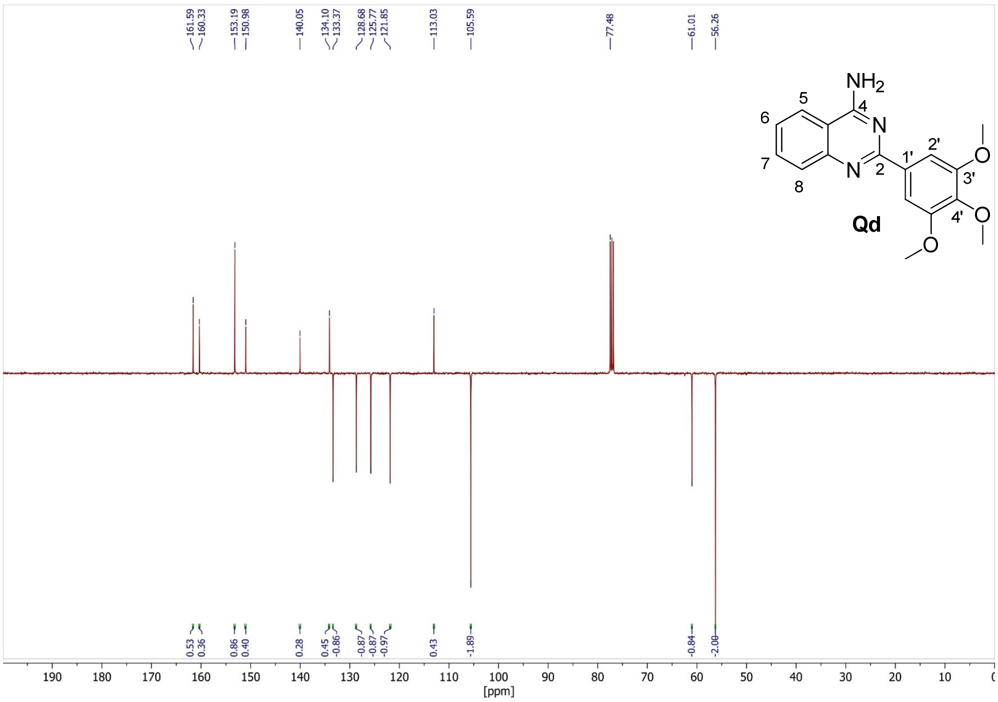

Figure S7. APT-$^{13}C\{^1H\}$ NMR spectrum of **Qd** in $CDCl_3$.

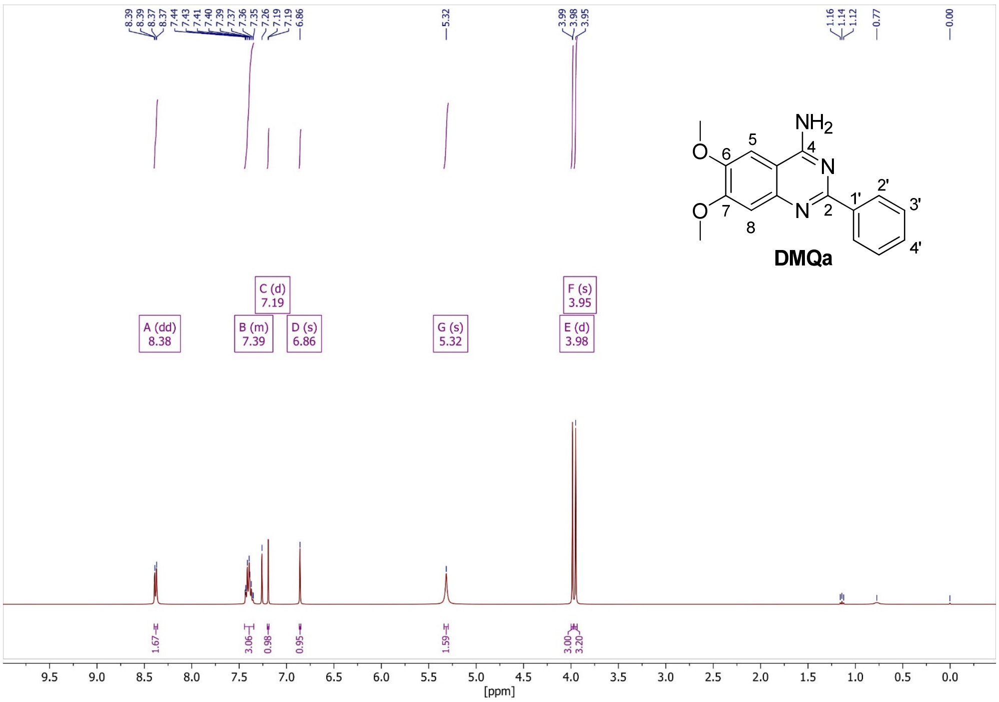

Figure S8. ^1H NMR spectrum of **DMQa** in $CDCl_3$.

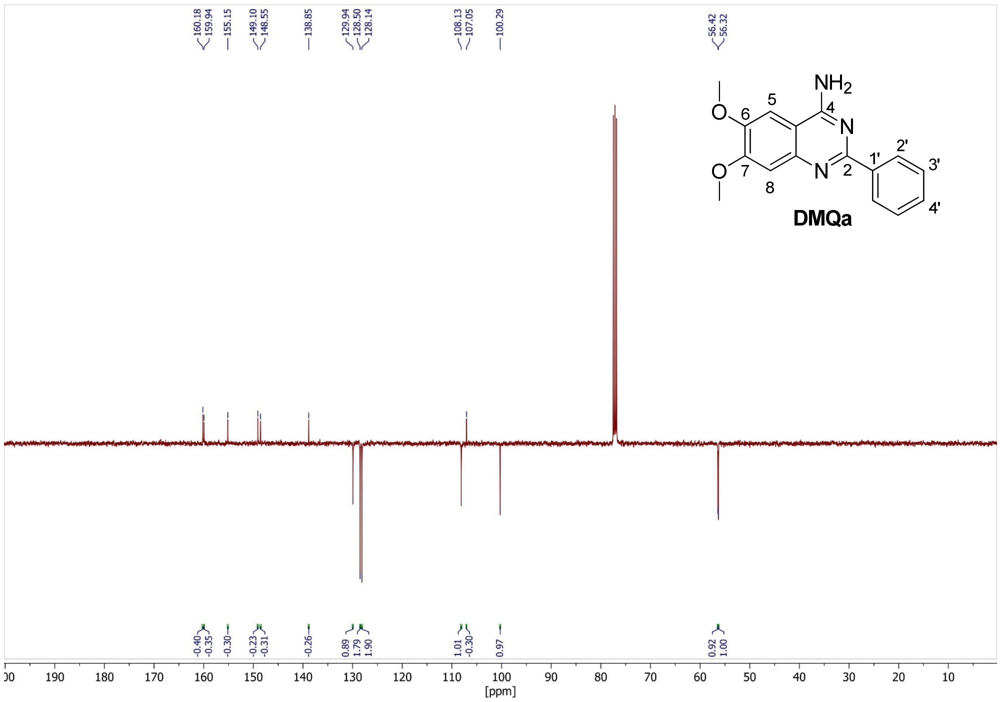

Figure S9. APT-$^{13}C\{^1H\}$ NMR spectrum of **DMQa** in $CDCl_3$.

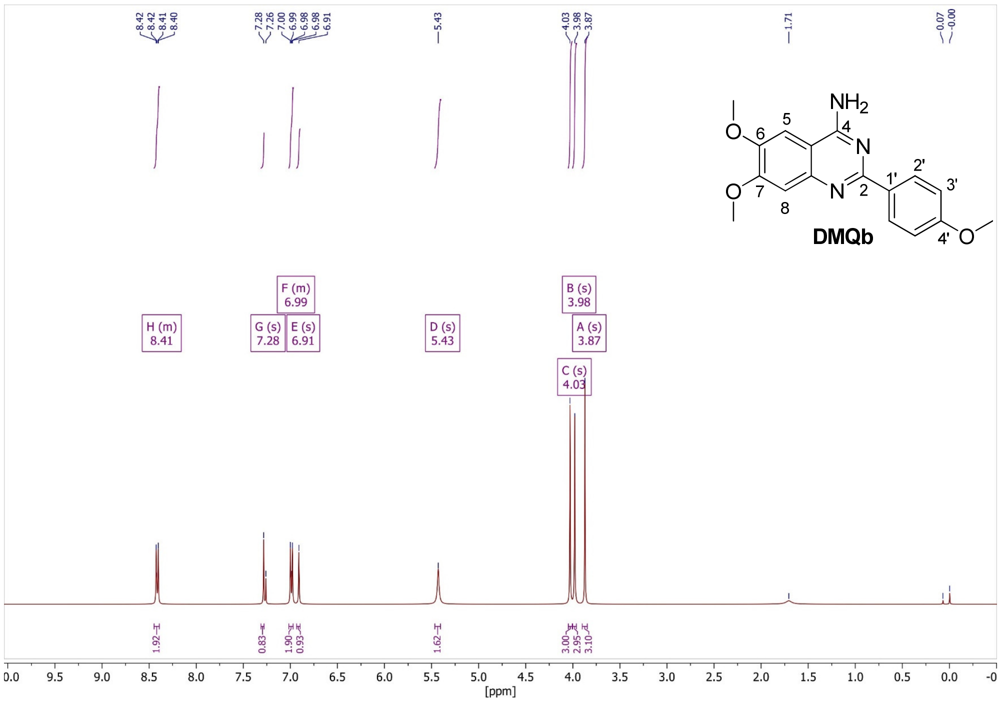

Figure S10. ^1H NMR spectrum of **DMQb** in $CDCl_3$.

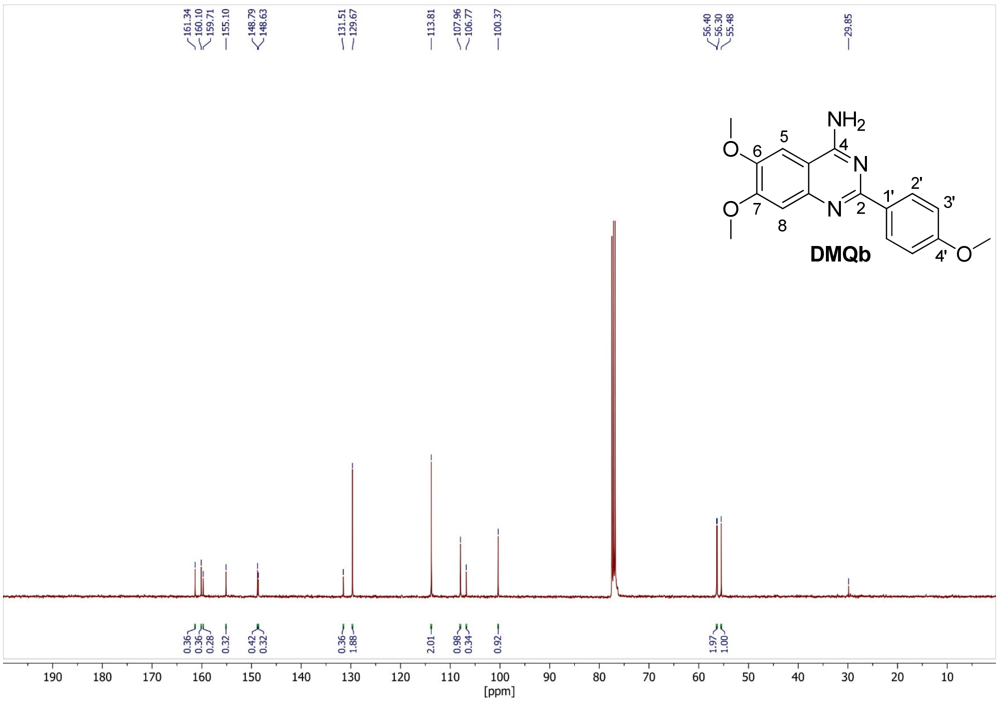

Figure S11. $^{13}C\{^{1}H\}$ NMR spectrum of **DMQb** in $CDCl_3$.

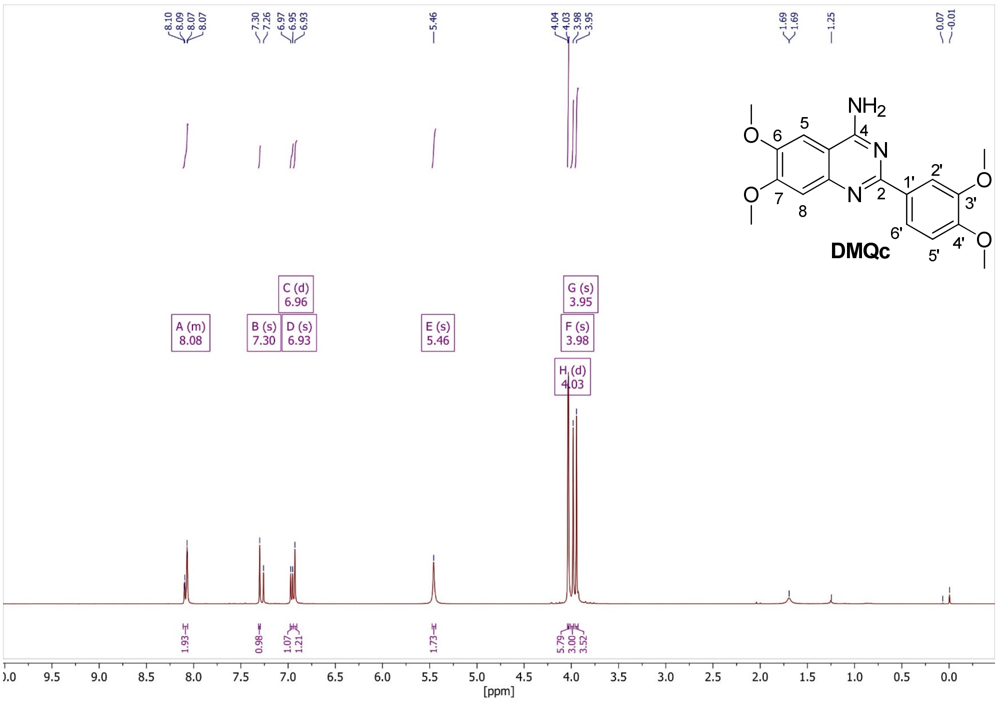

Figure S12. ^{1}H NMR spectrum of **DMQc** in $CDCl_3$.

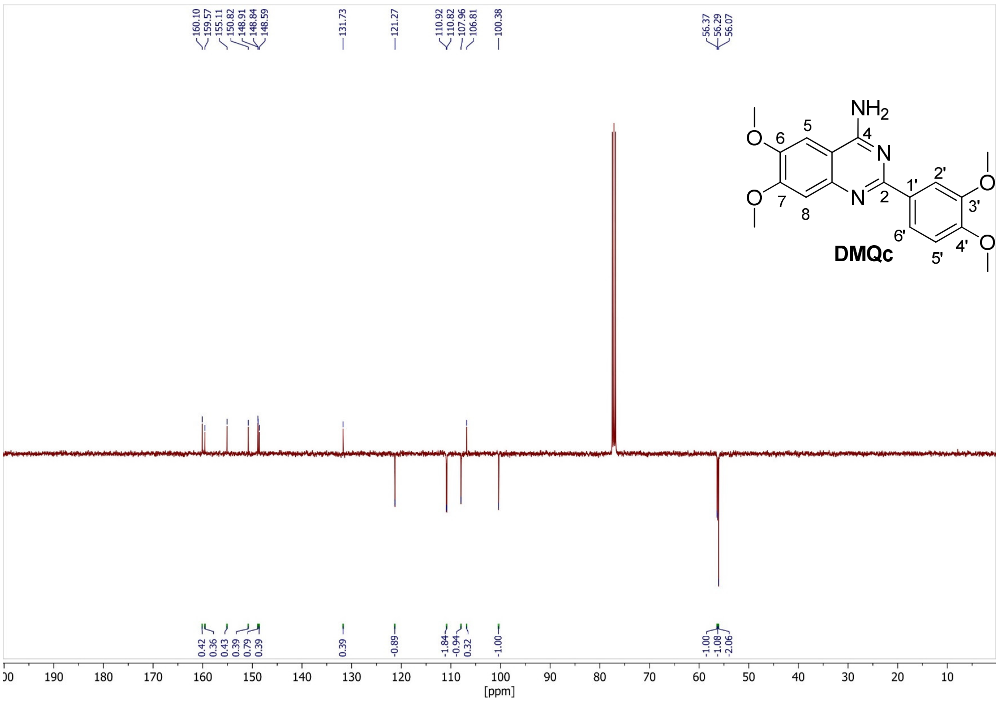

Figure S13. APT-$^{13}C\{^1H\}$ NMR spectrum of **DMQc** in $CDCl_3$.

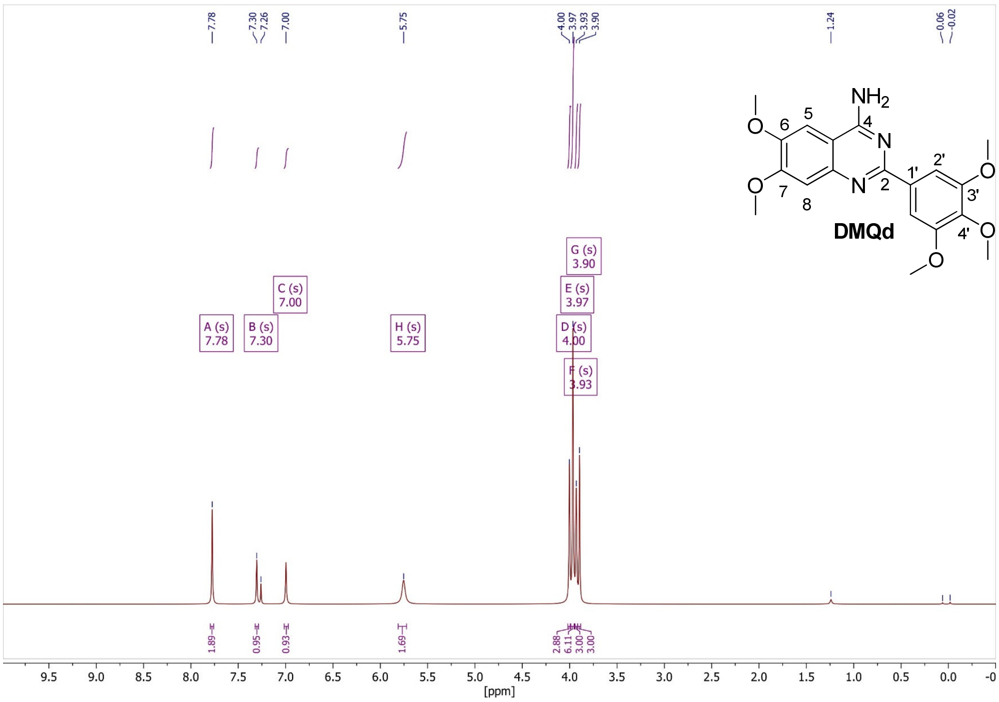

Figure S14. ^1H NMR spectrum of **DMQd** in $CDCl_3$.

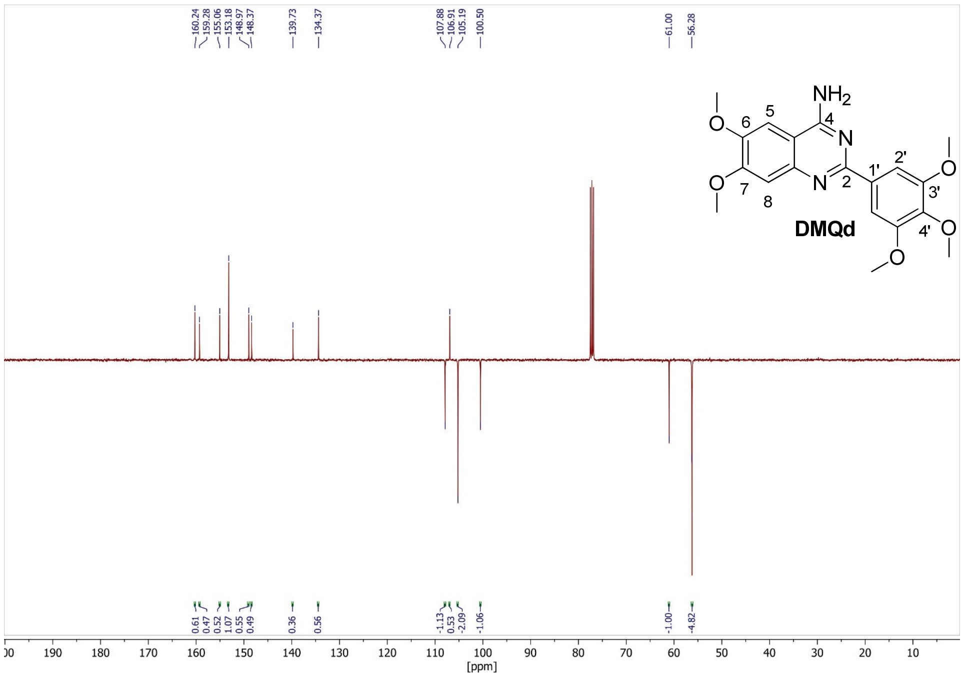

Figure S15. APT-$^{13}C\{^1H\}$ NMR spectrum of **DMQd** in $CDCl_3$.

2. NMR spectra of *N*-carboranyl quinazoline-4-amines QCa-d and DMQCa-d

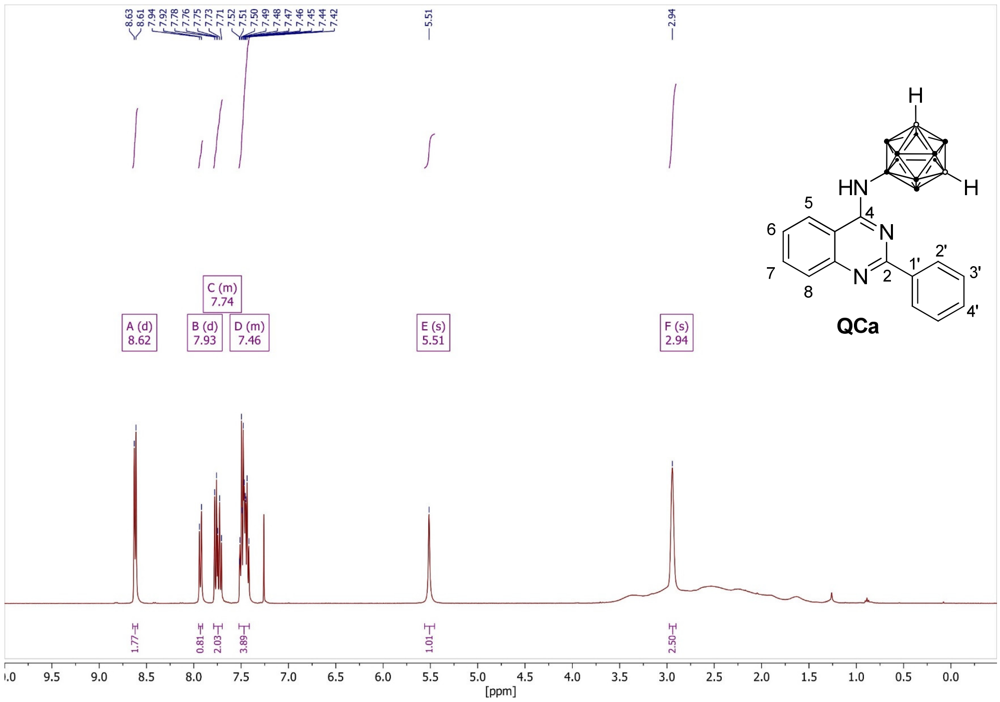

Figure S16. ^{1}H NMR spectrum of **QCa** in $CDCl_3$.

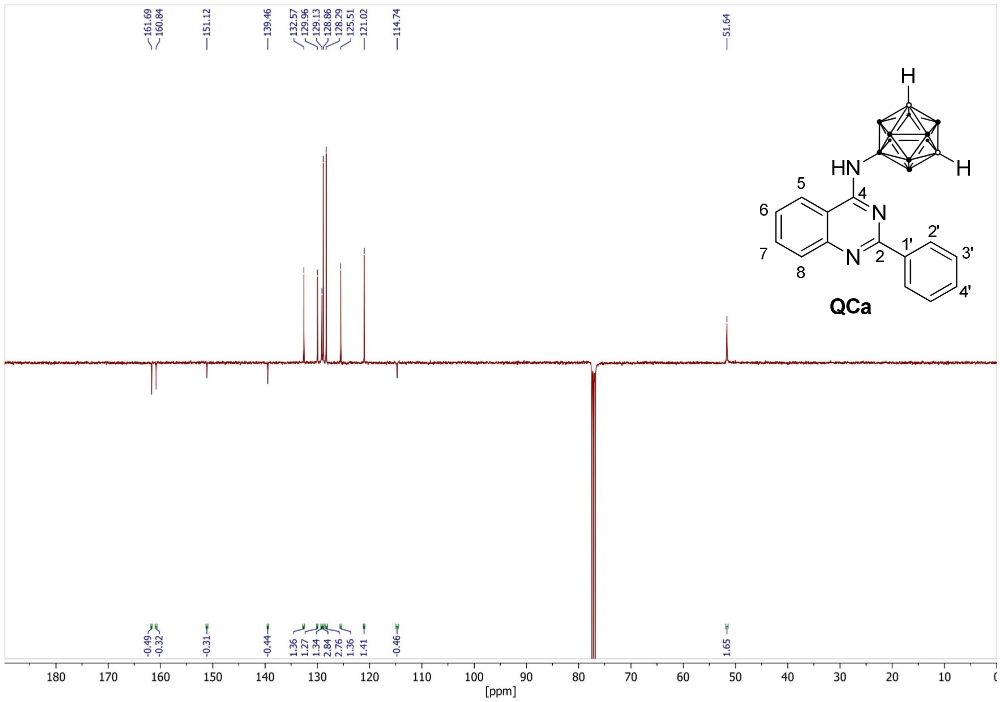

Figure S17. APT-$^{13}C\{^{1}H\}$ NMR spectrum of **QCa** in $CDCl_3$.

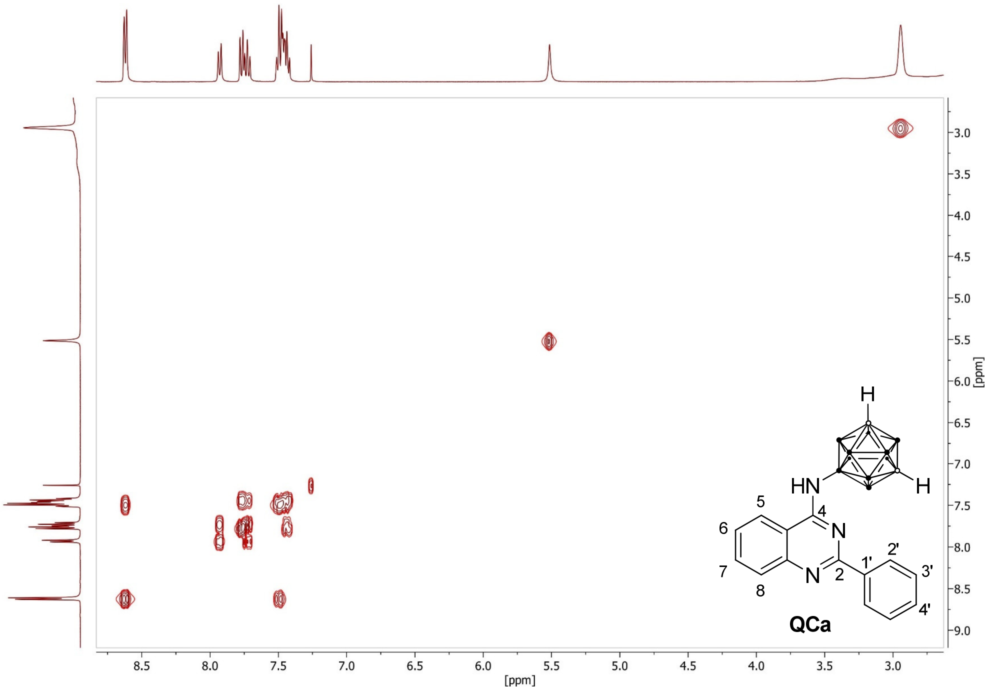

Figure S18. ^{1}H-COSY NMR spectrum of **QCa** in $CDCl_3$.

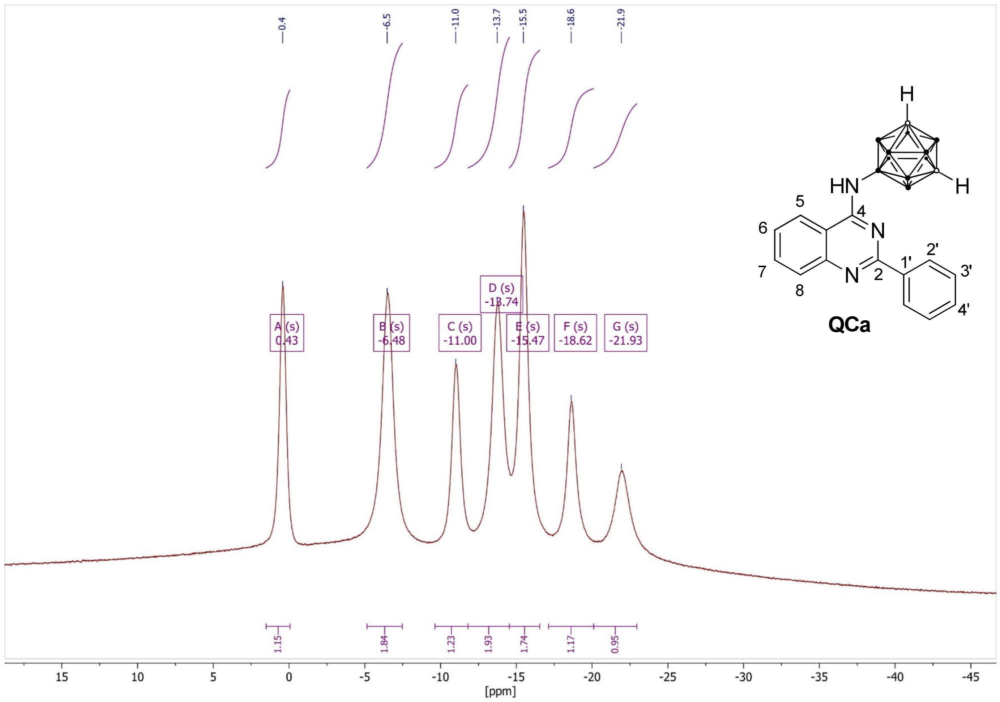

Figure S19. $^{11}B\{^1H\}$ NMR spectrum of **QCa** in $CDCl_3$.

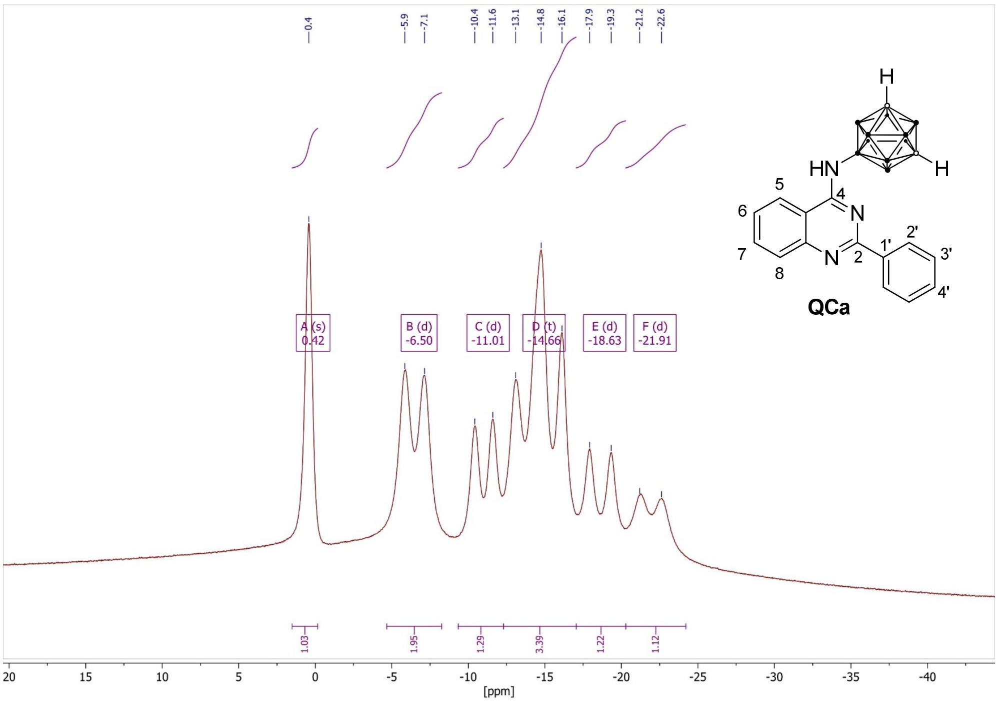

Figure S20. ¹¹B NMR spectrum of **QCa** in $CDCl_3$.

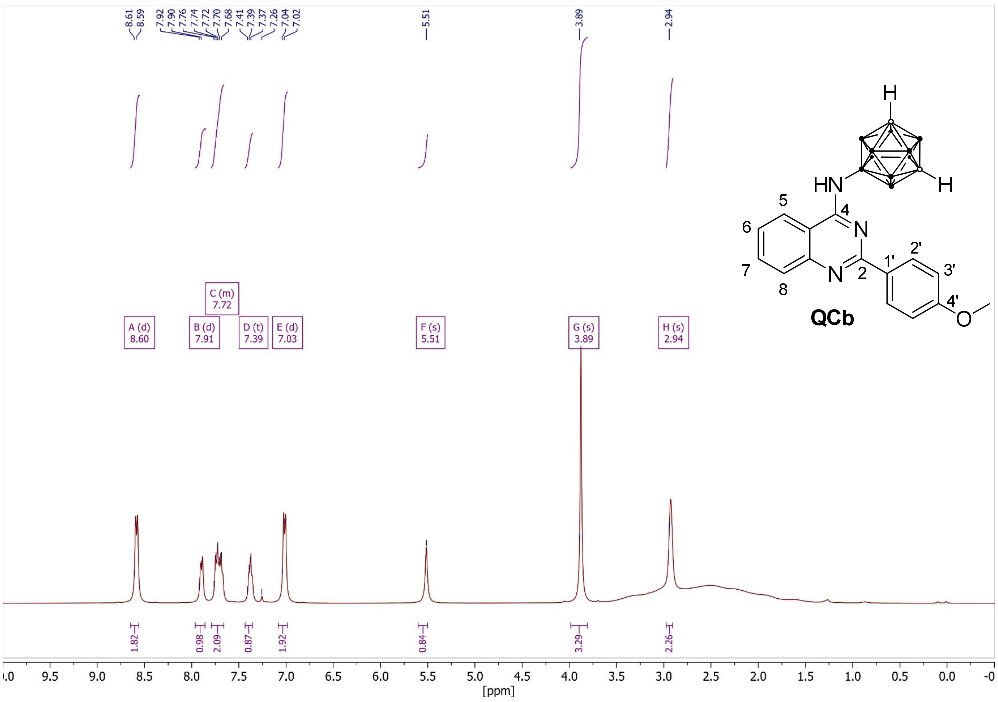

Figure S21. ¹H NMR spectrum of **QCb** in $CDCl_3$.

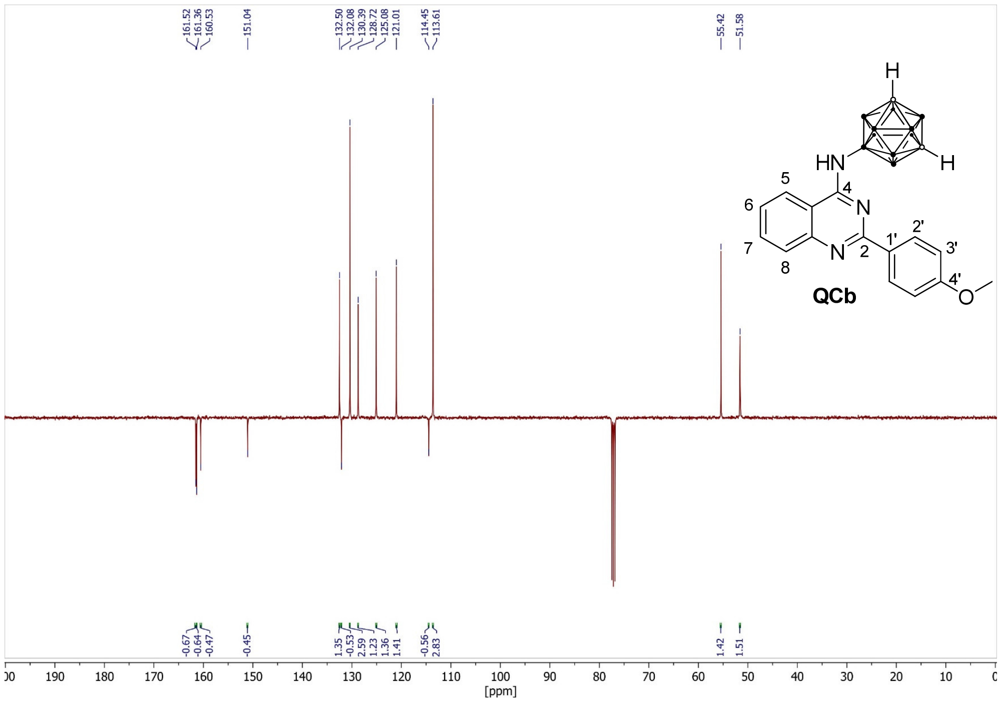

Figure S22. APT-$^{13}C\{^1H\}$ NMR spectrum of **QCb** in $CDCl_3$.

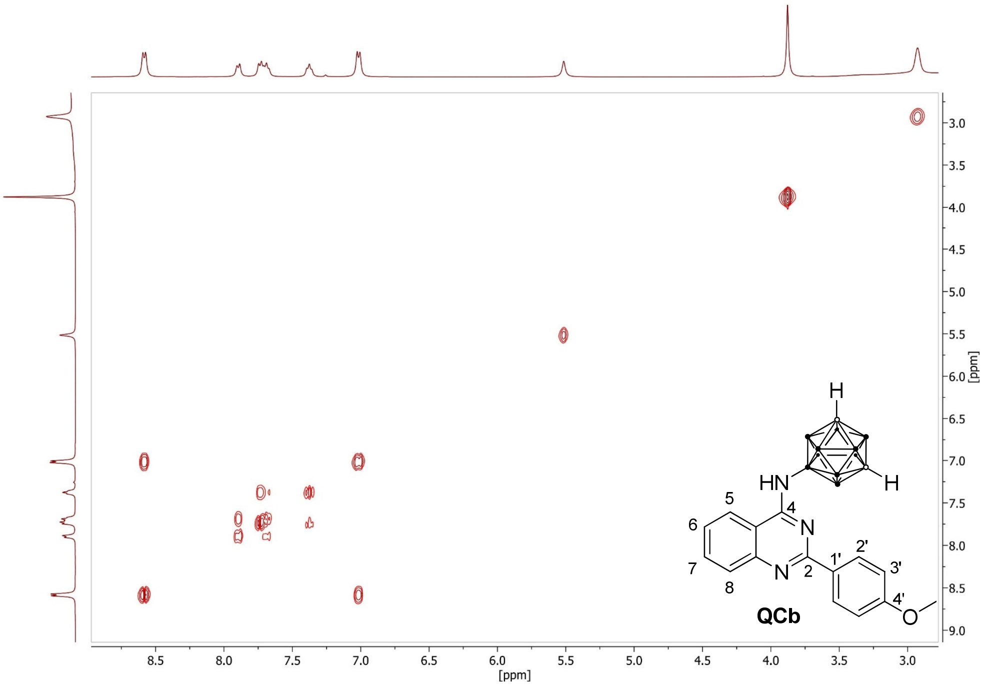

Figure S23. ^1H-COSY NMR spectrum of **QCb** in $CDCl_3$.

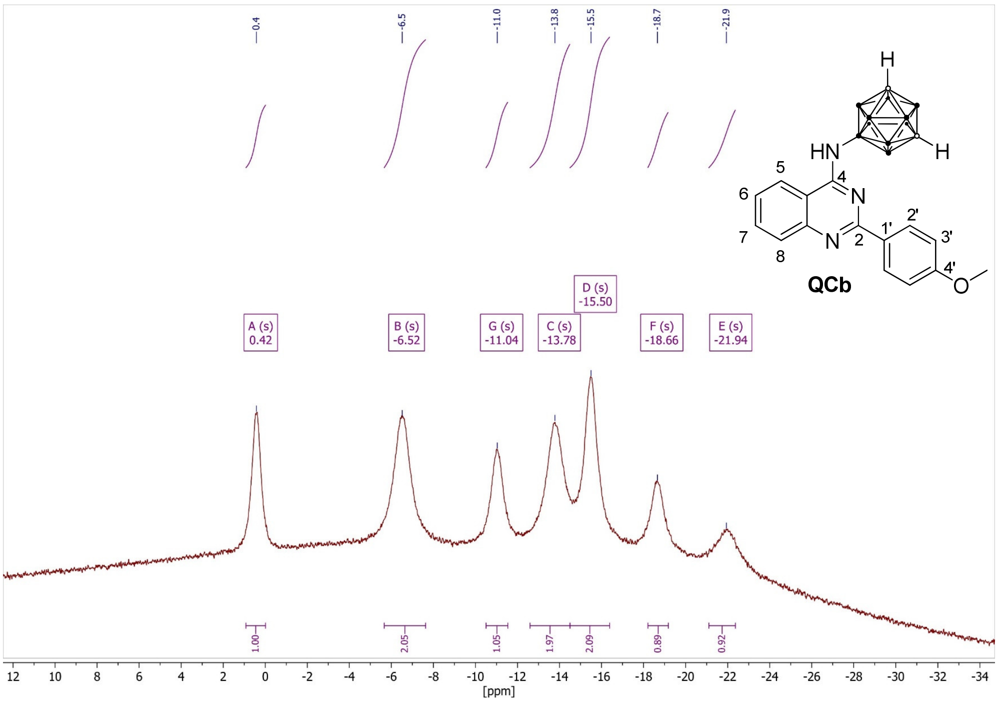

Figure S24. $^{11}B\{^{1}H\}$ NMR spectrum of **QCb** in $CDCl_3$.

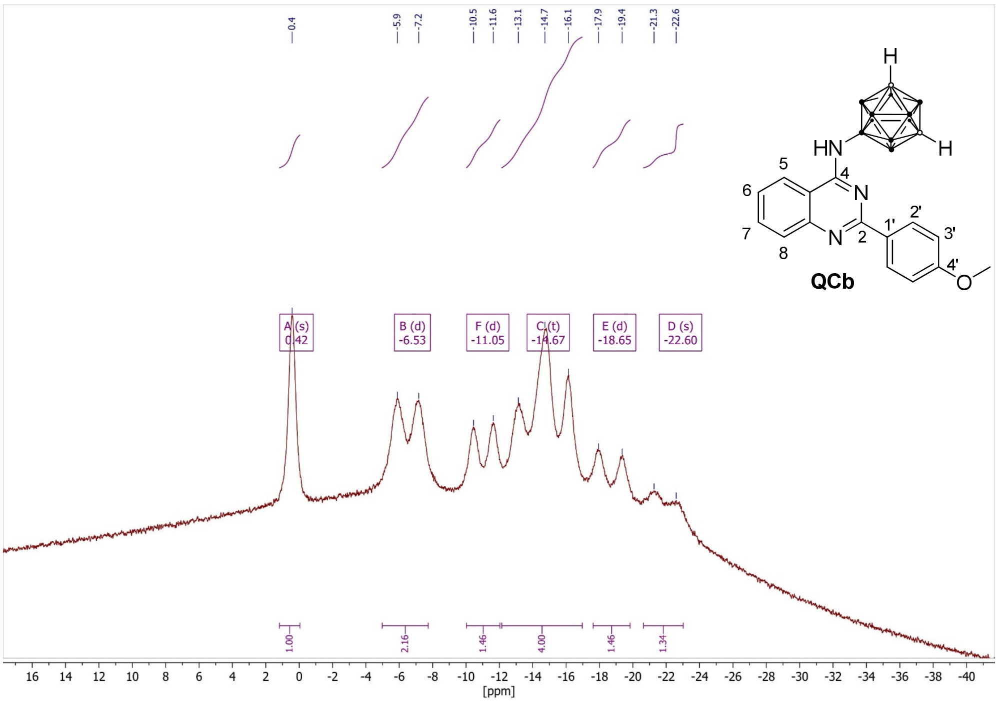

Figure S56. ^{11}B NMR spectrum of **QCb** in $CDCl_3$.

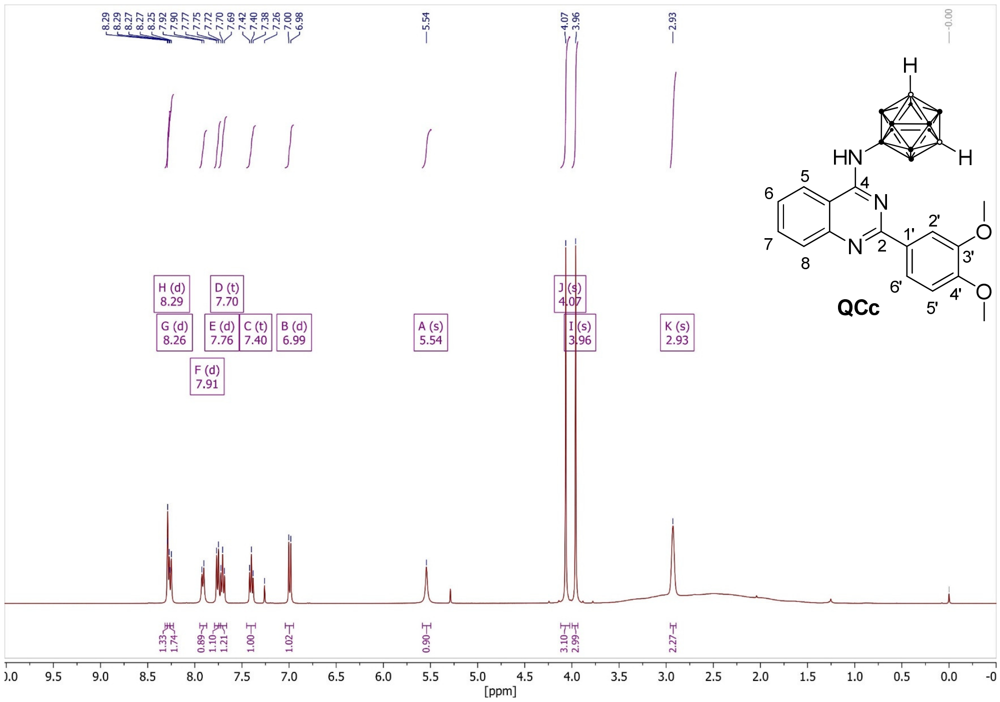

Figure S26. ^{1}H NMR spectrum of **QCc** in $CDCl_3$.

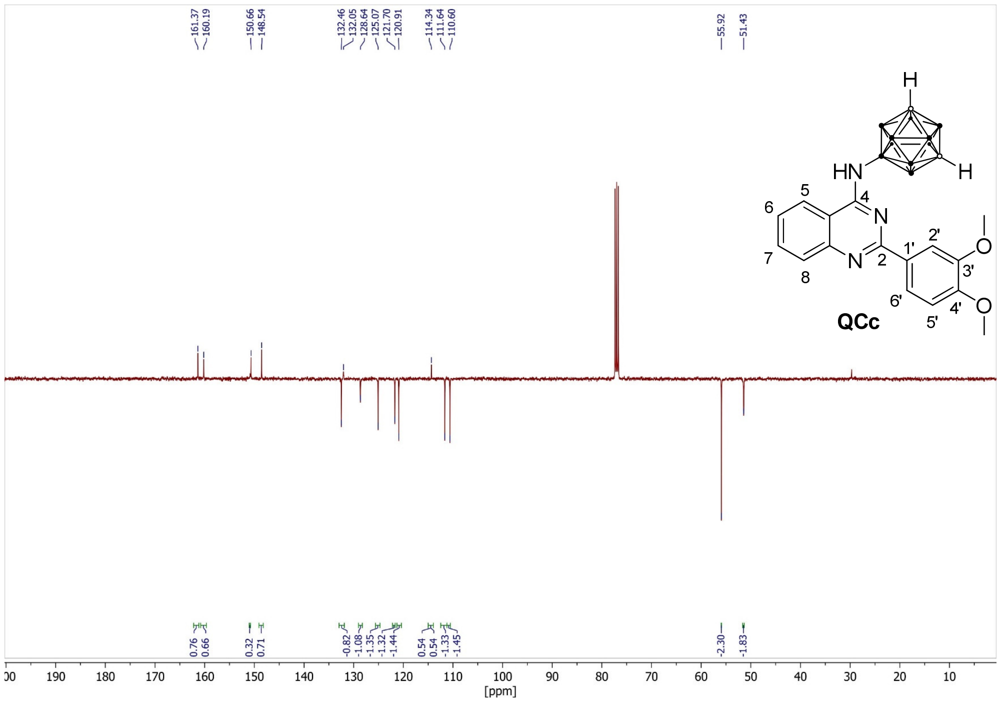

Figure S27. APT-$^{13}C\{^{1}H\}$ NMR spectrum of **QCc** in $CDCl_3$.

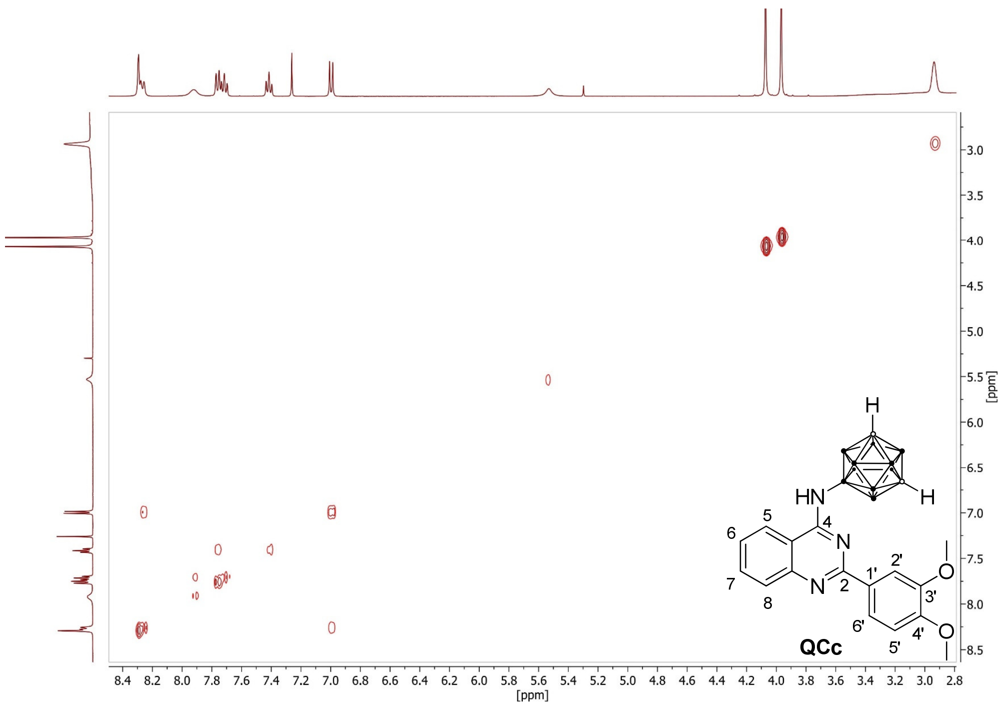

Figure S28. ^{1}H-COSY NMR spectrum of **QCc** in $CDCl_3$.

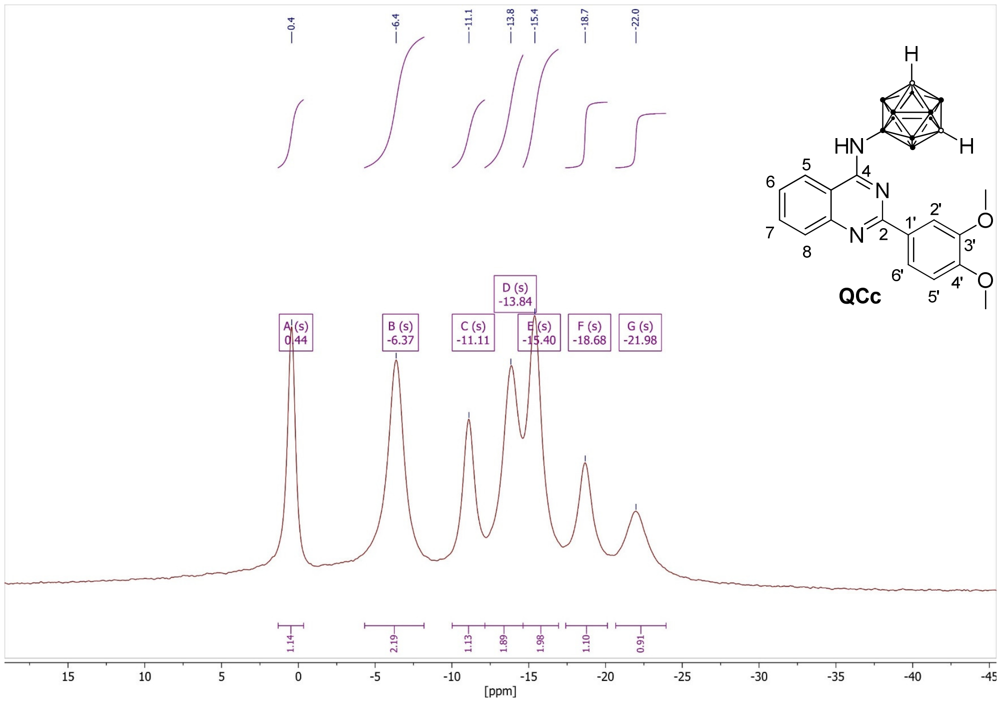

Figure S29. ^{11}B{^{1}H} NMR spectrum of **QCc** in $CDCl_3$.

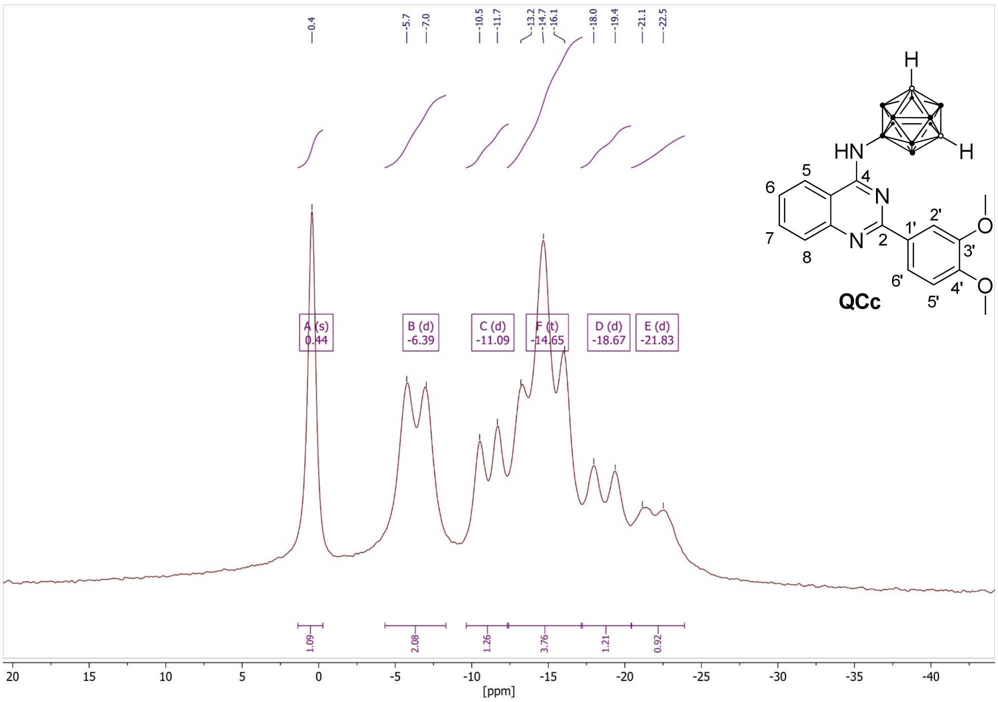

Figure S30. ^{11}B NMR spectrum of **QCc** in $CDCl_3$.

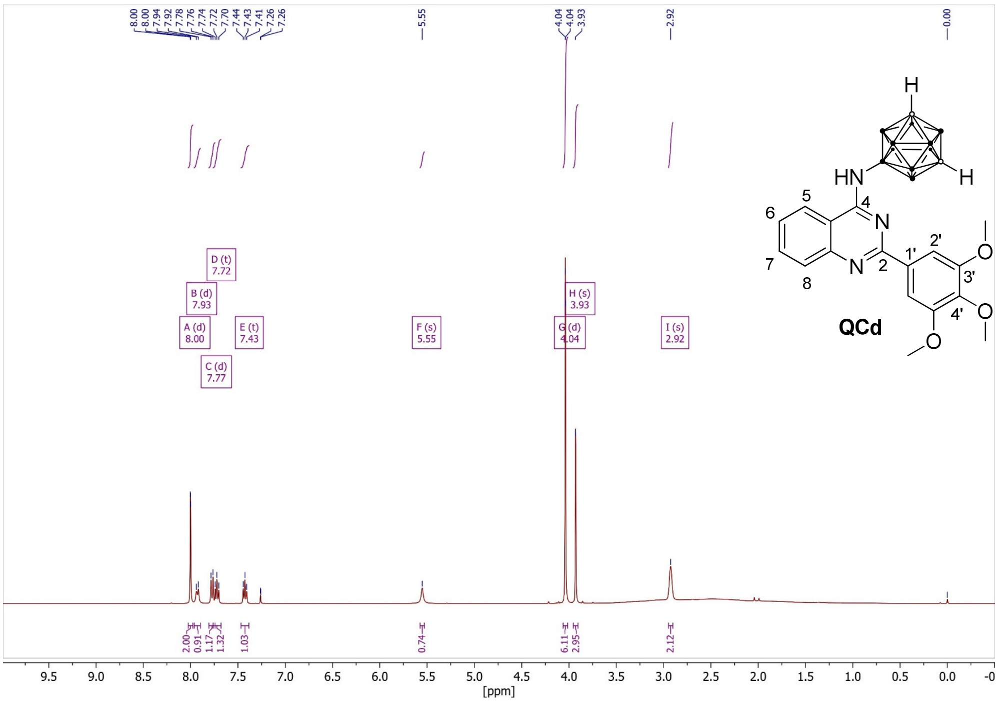

Figure S31. ^{1}H NMR spectrum of **QCd** in $CDCl_3$.

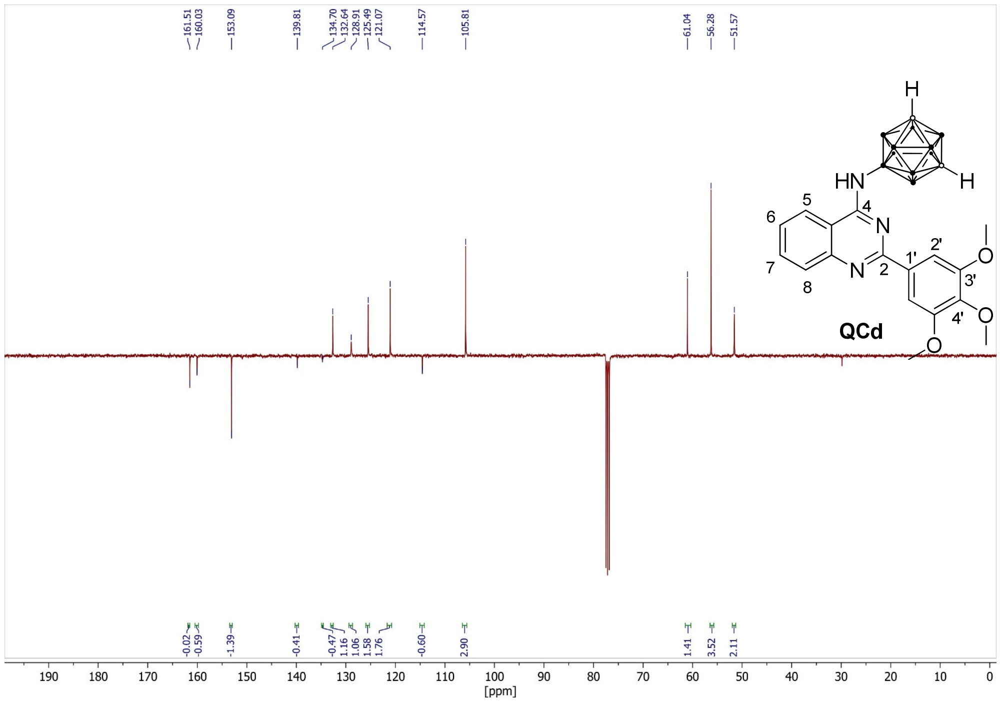

Figure S32. APT-$^{13}C\{^{1}H\}$ NMR spectrum of **QCd** in $CDCl_3$.

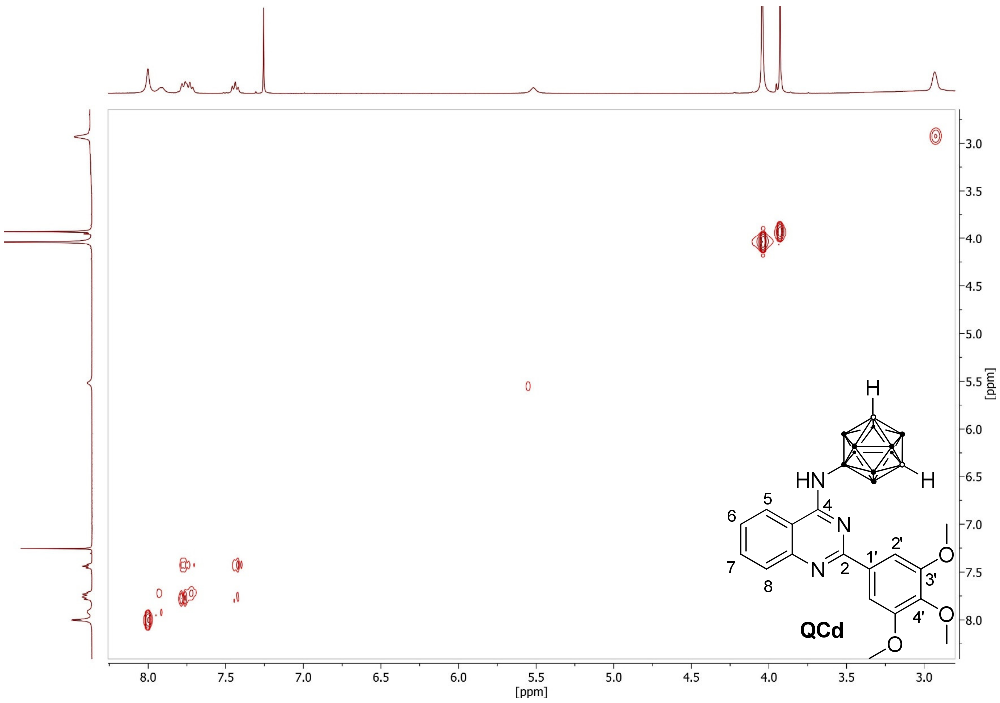

Figure S33. ^{1}H-COSY NMR spectrum of **QCd** in $CDCl_3$.

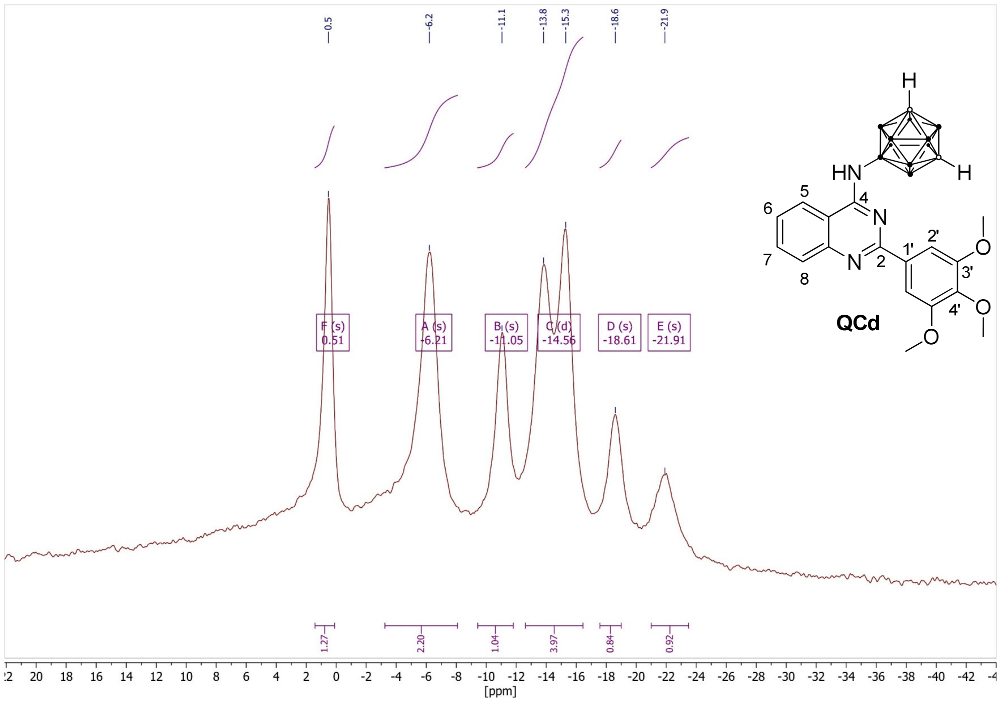

Figure S34. $^{11}B\{^{1}H\}$ NMR spectrum of **QCd** in $CDCl_3$.

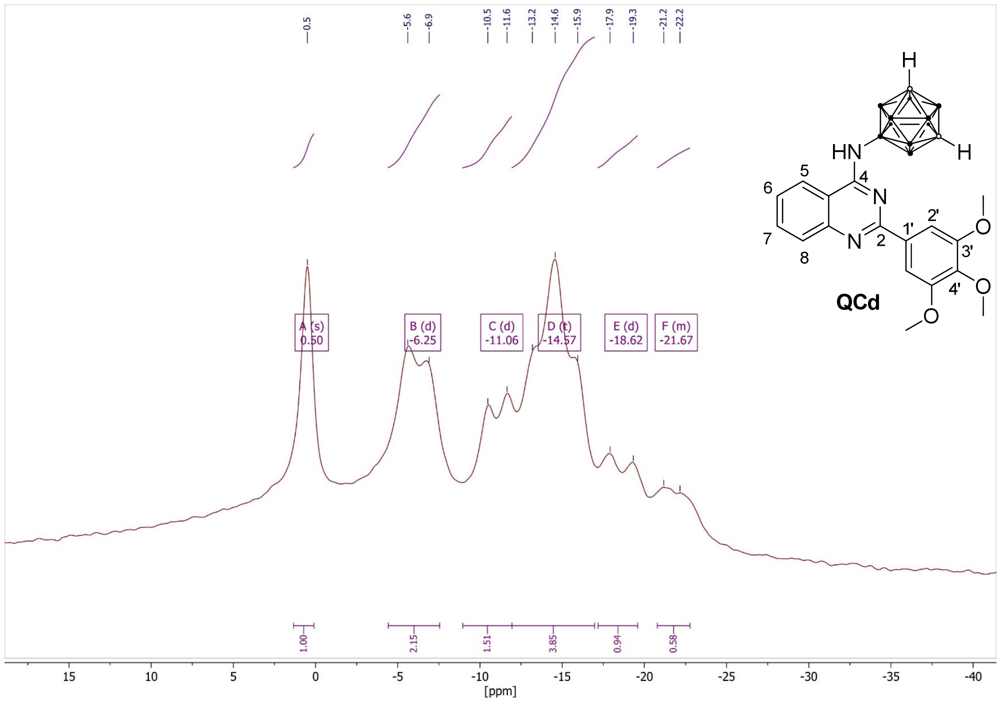

Figure S35. ^{11}B NMR spectrum of **QCd** in $CDCl_3$.

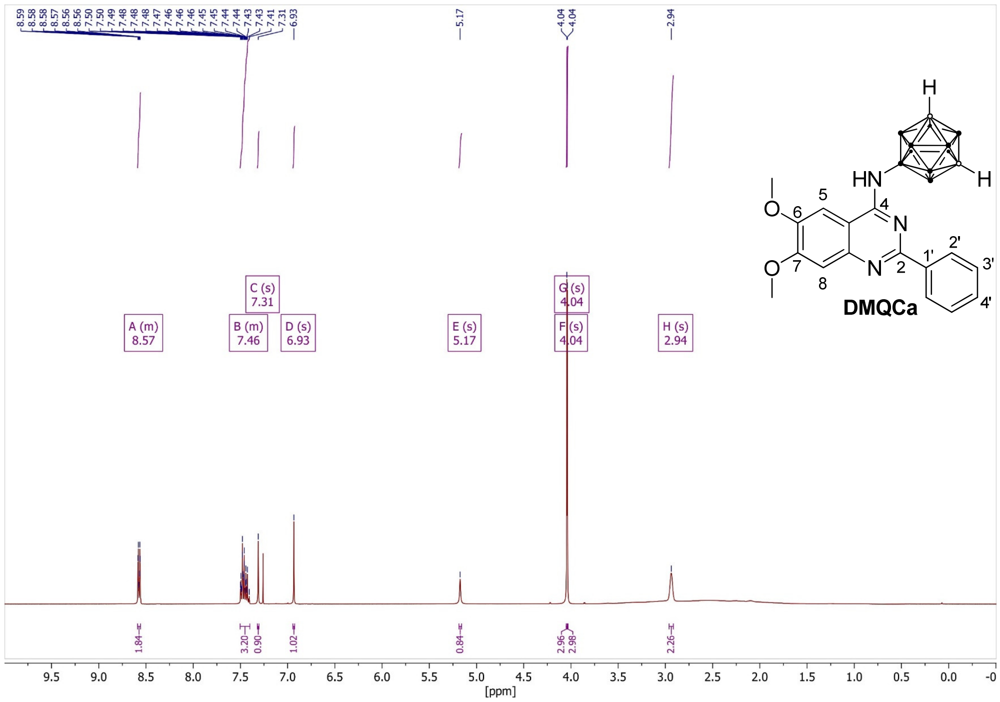

Figure S36. ^{1}H NMR spectrum of **DMQCa** in $CDCl_3$.

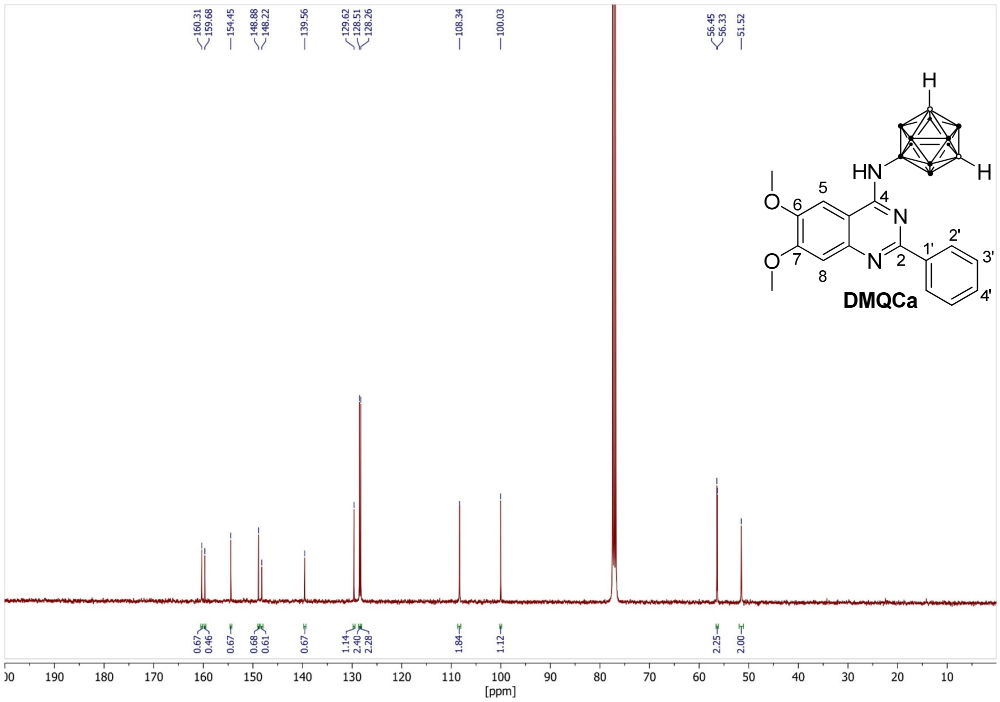

Figure S37. $^{13}C\{^{1}H\}$ NMR spectrum of **DMQCa** in $CDCl_3$.

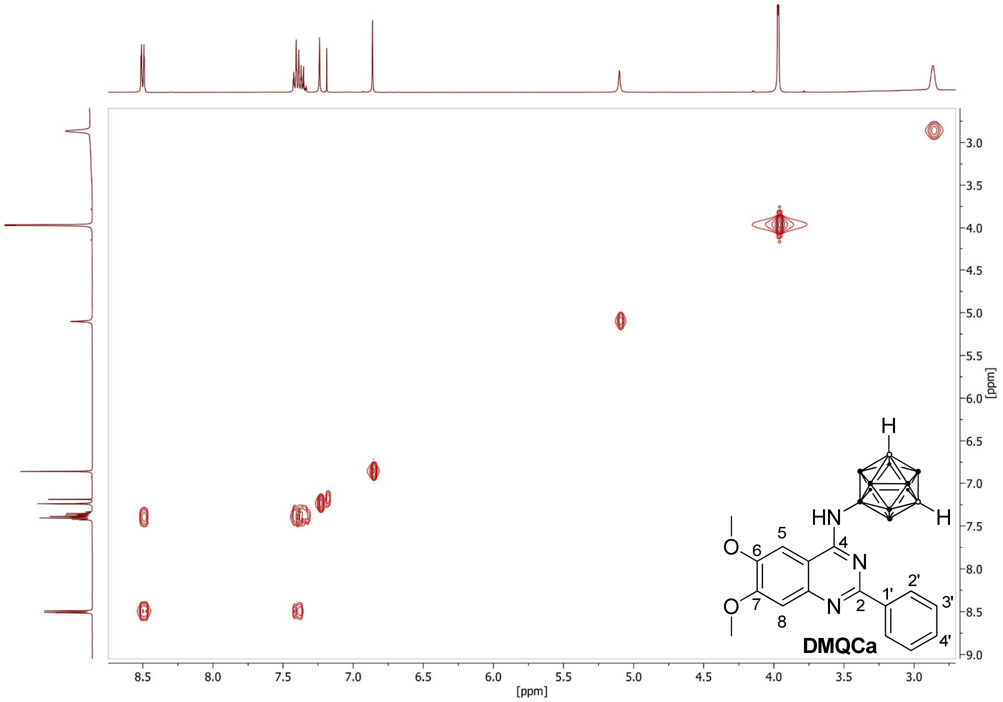

Figure S38. ^{1}H-COSY NMR spectrum of **DMQCa** in $CDCl_3$.

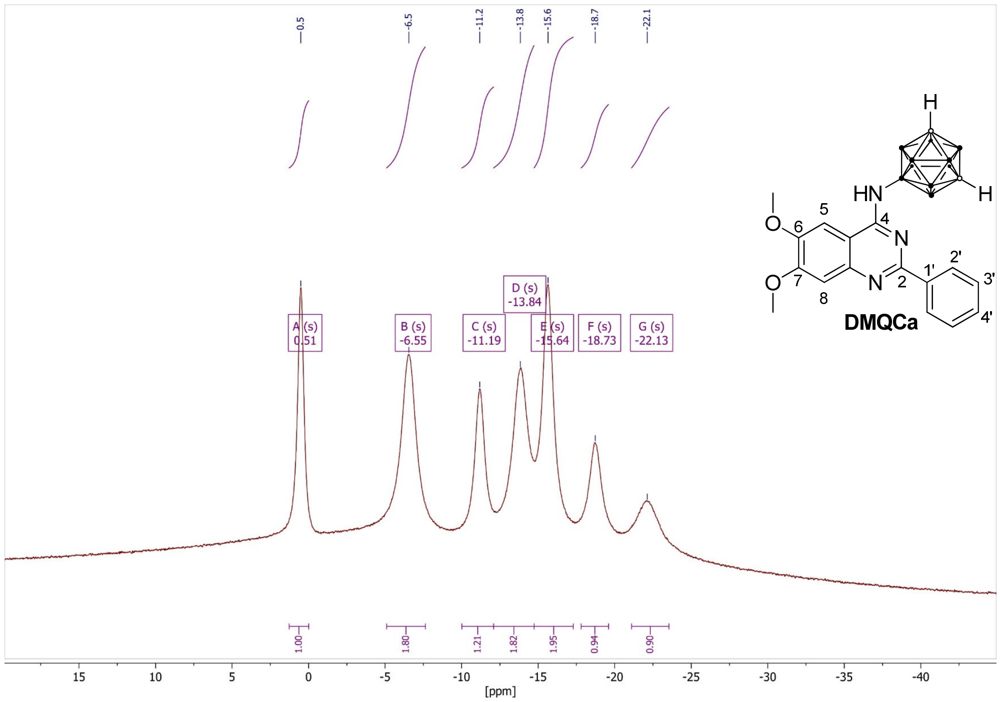

Figure S39. $^{11}B\{^1H\}$ NMR spectrum of **DMQCa** in $CDCl_3$.

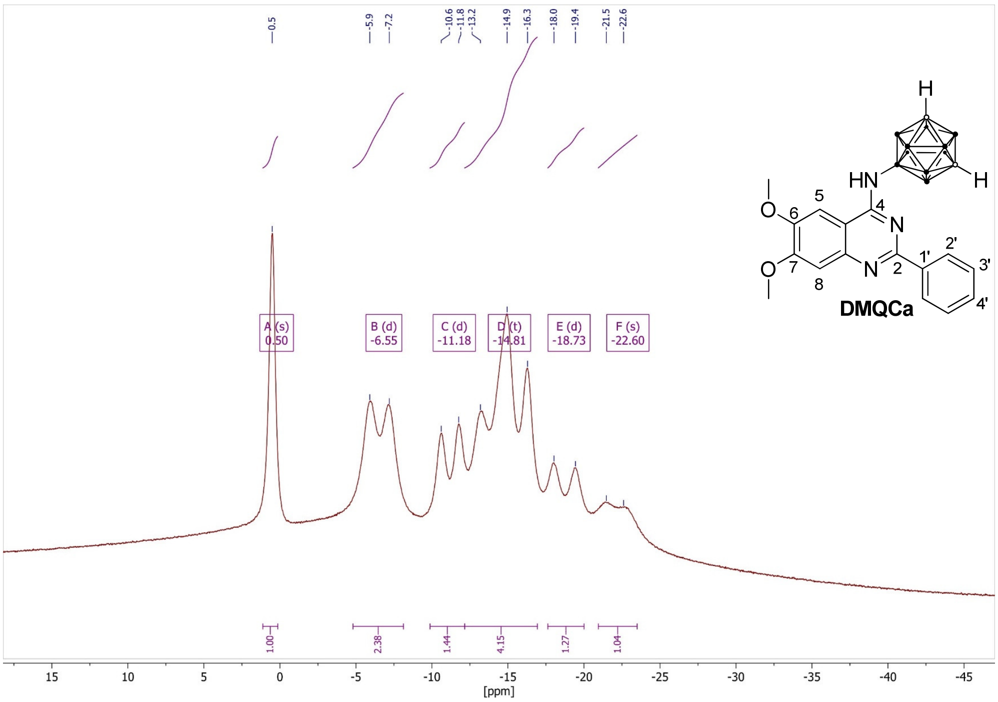

Figure S40. ^{11}B NMR spectrum of **DMQCa** in $CDCl_3$.

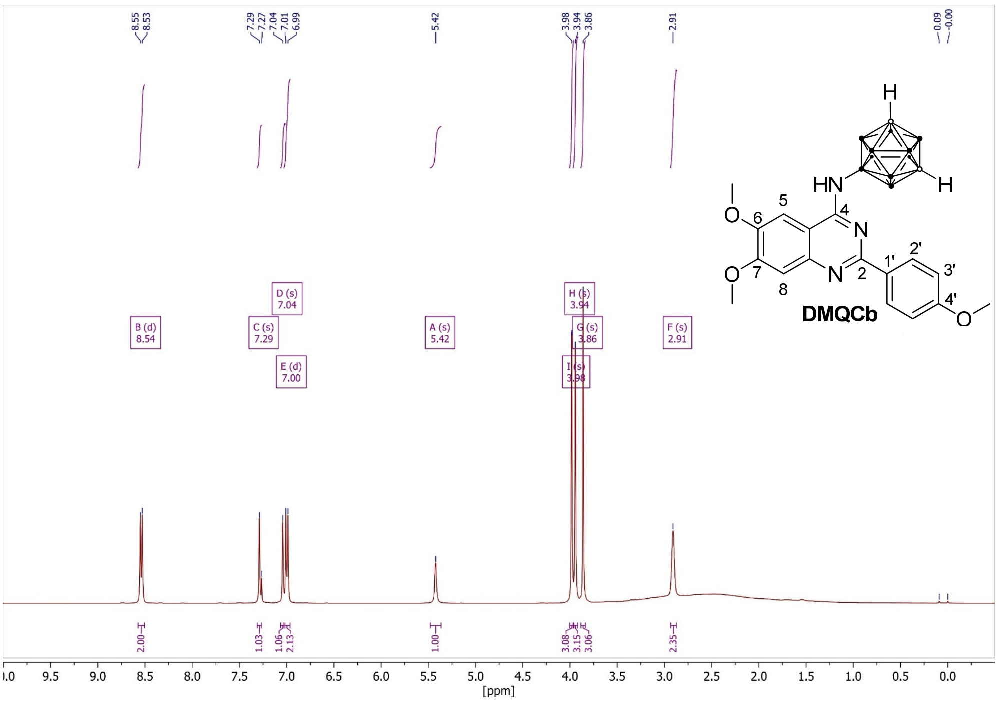

Figure S41. ^{1}H NMR spectrum of **DMQCb** in $CDCl_3$.

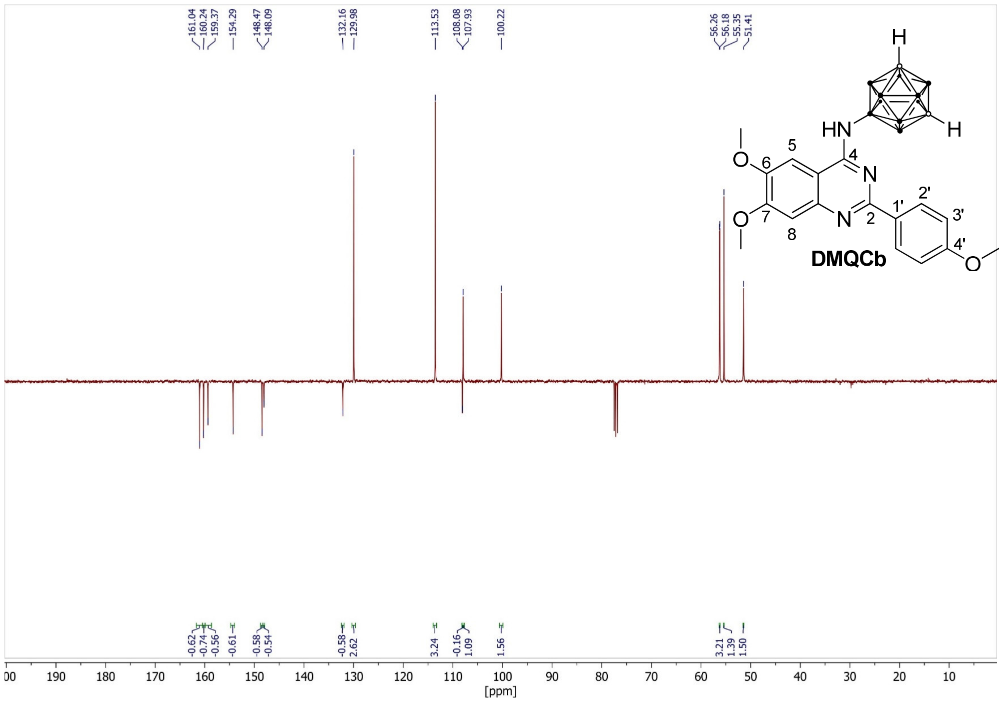

Figure S42. APT-$^{13}C\{^{1}H\}$ NMR spectrum of **DMQCb** in $CDCl_3$.

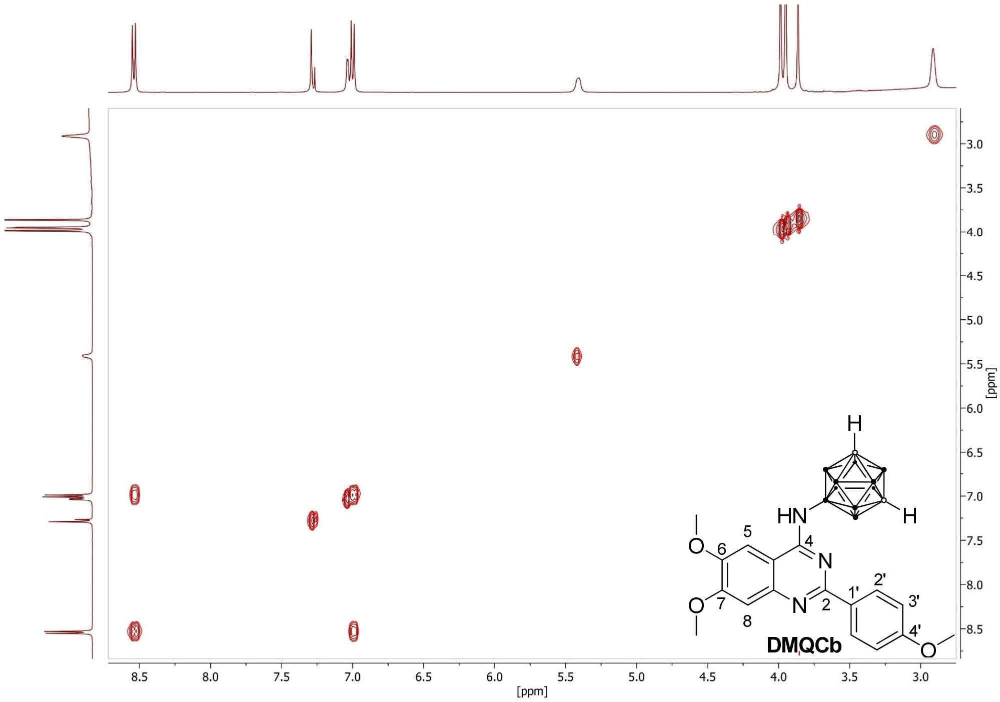

Figure S43. ^{1}H-COSY NMR spectrum of **DMQCb** in $CDCl_3$.

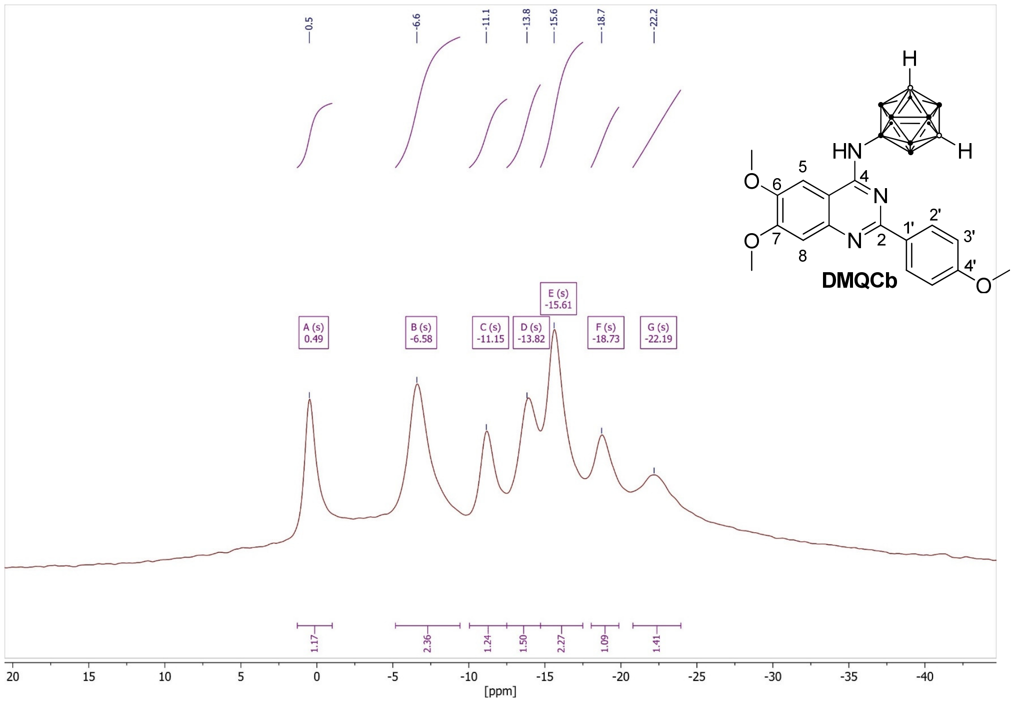

Figure S44. $^{11}B\{^{1}H\}$ NMR spectrum of **DMQCb** in $CDCl_3$.

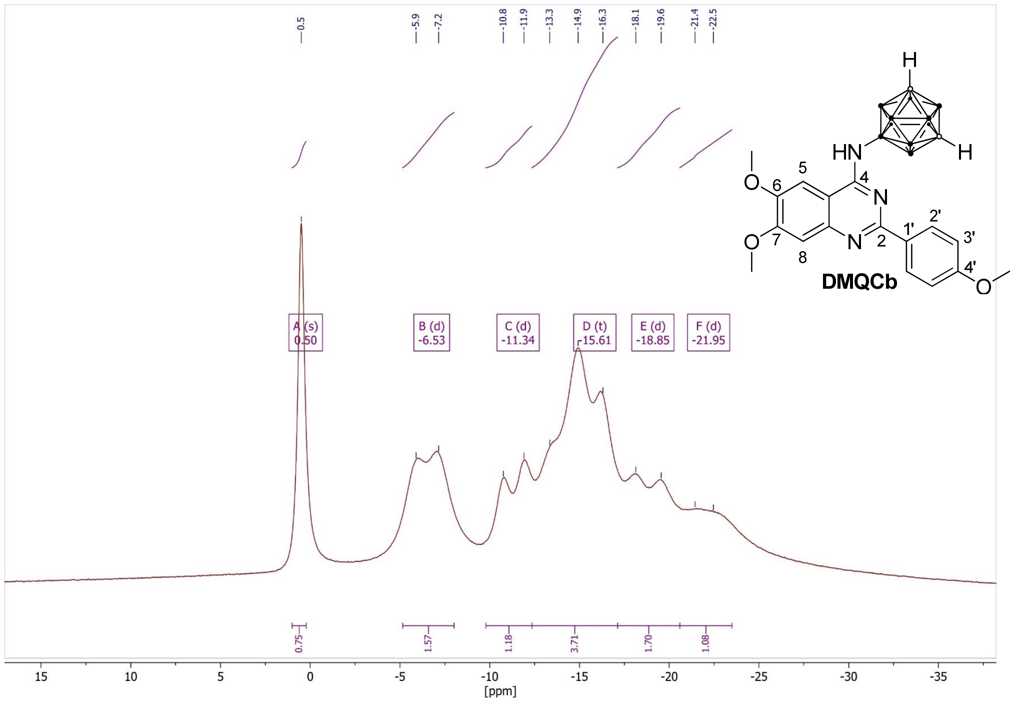

Figure S45. ^{11}B NMR spectrum of **DMQCb** in $CDCl_3$.

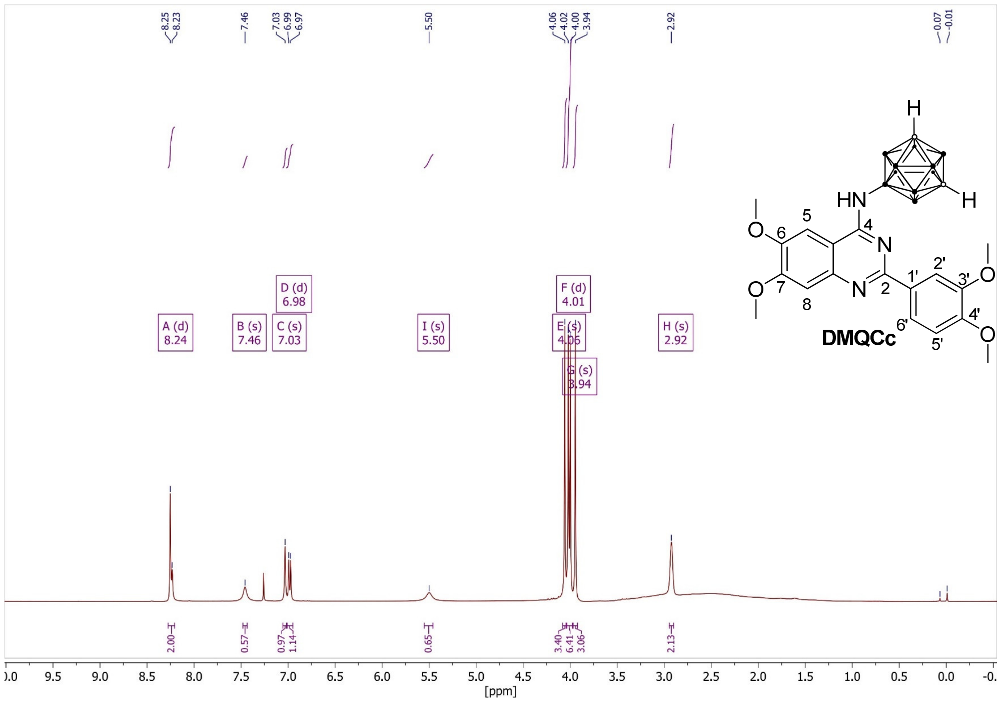

Figure S46. ^{1}H NMR spectrum of **DMQCc** in $CDCl_3$.

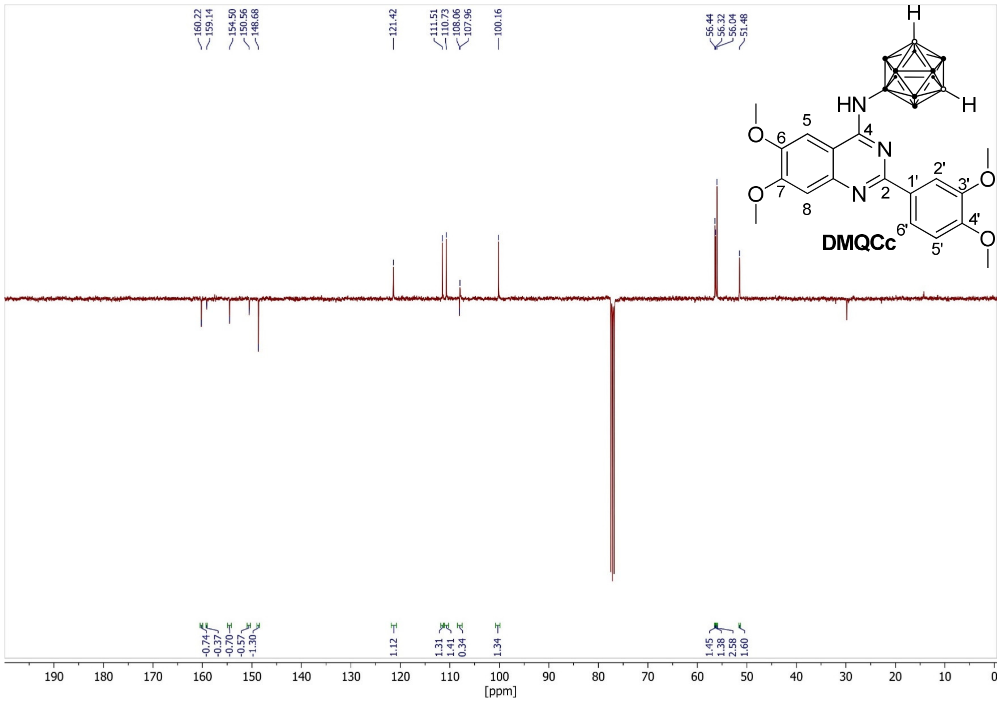

Figure S47. APT-$^{13}C\{^{1}H\}$ NMR spectrum of **DMQCc** in $CDCl_3$.

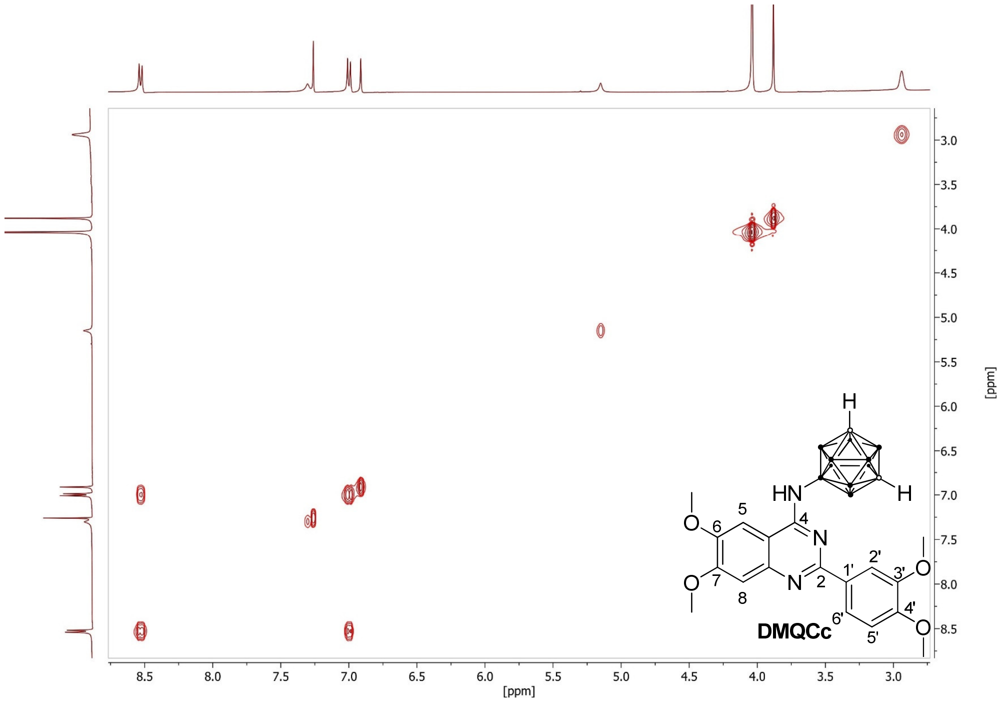

Figure S48. ^{1}H-COSY NMR spectrum of **DMQCc** in $CDCl_3$.

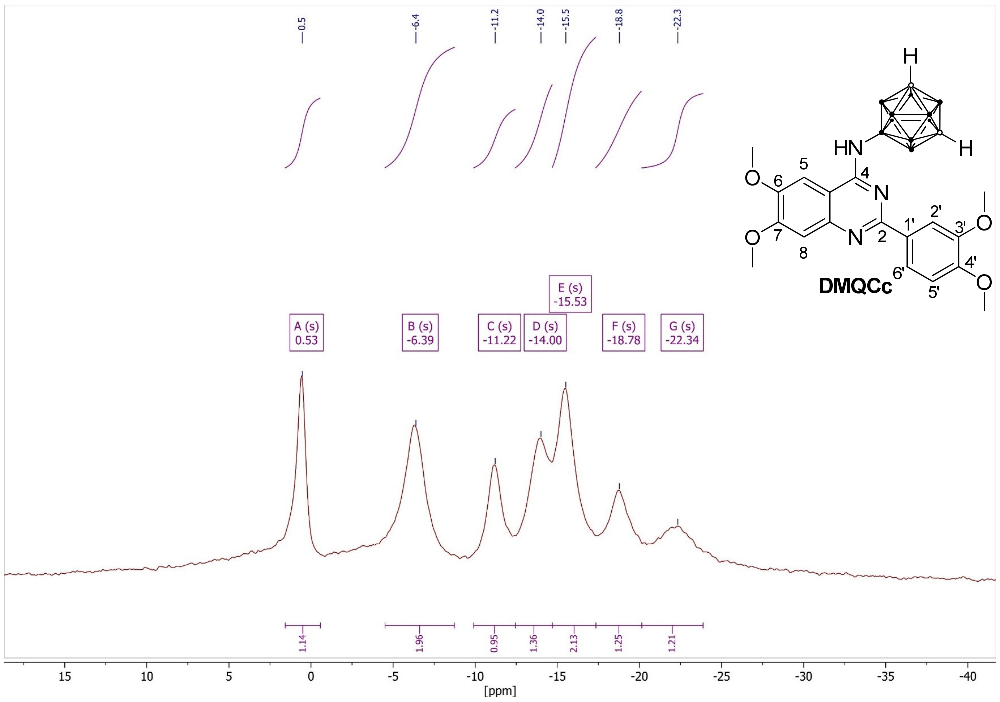

Figure S49. $^{11}B\{^{1}H\}$ NMR spectrum of **DMQCc** in $CDCl_3$.

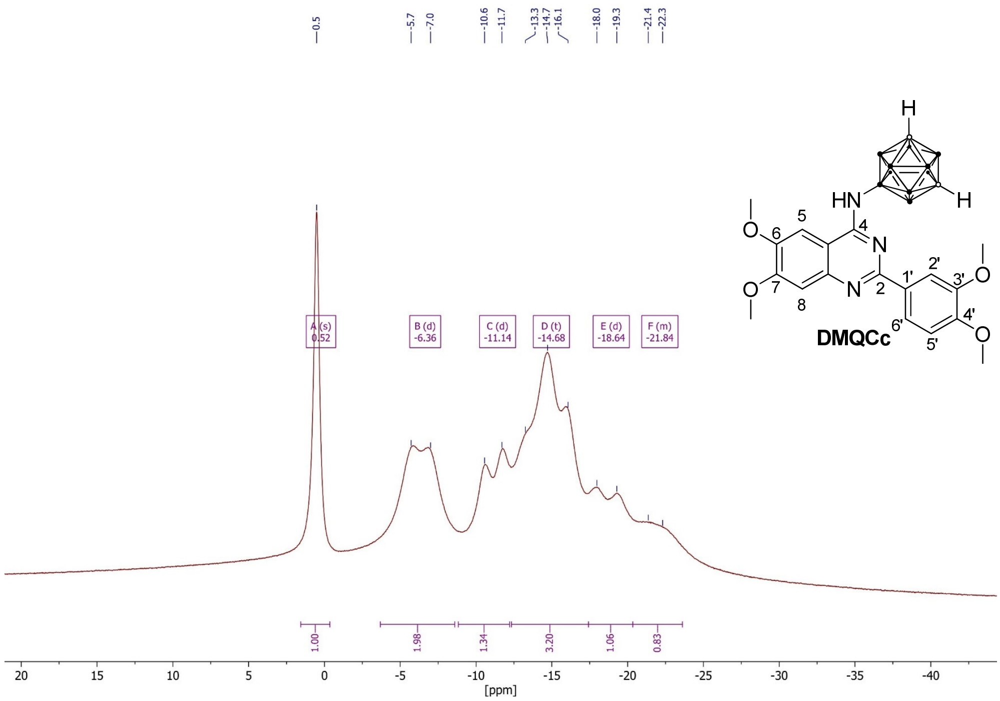

Figure S50. ^{11}B NMR spectrum of **DMQCc** in $CDCl_3$.

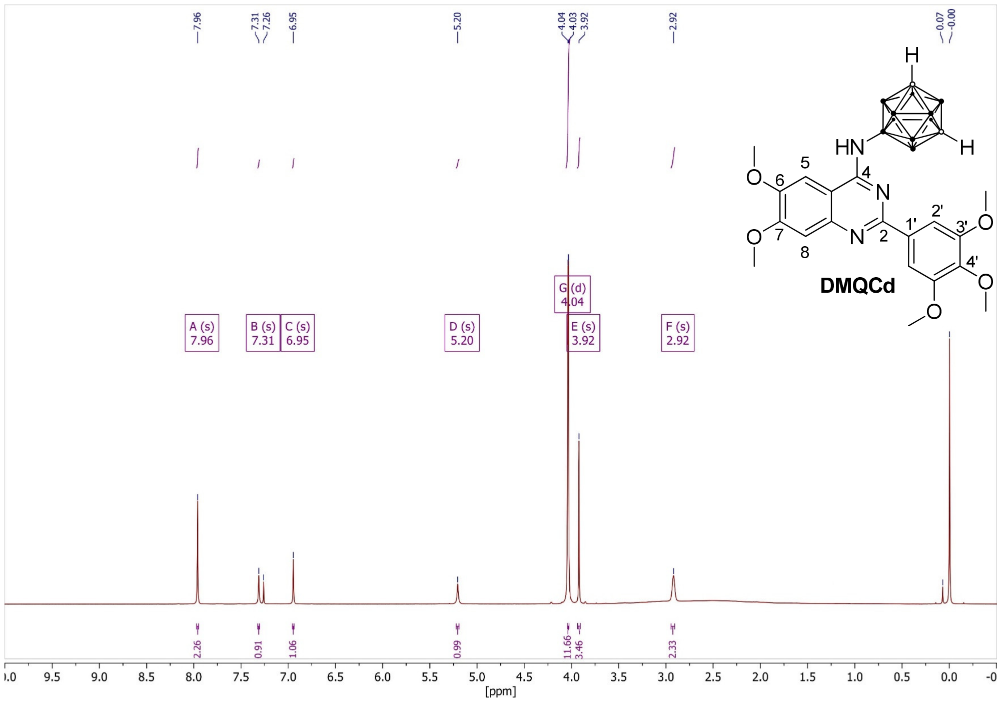

Figure S51. ^{1}H NMR spectrum of **DMQCd** in $CDCl_3$.

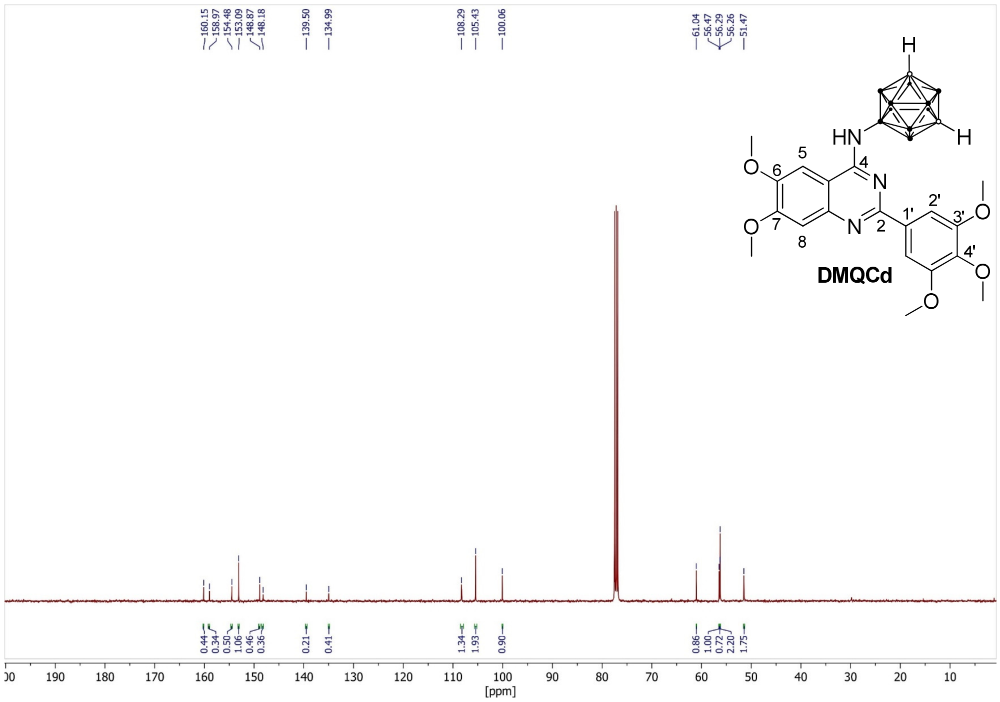

Figure S52. $^{13}C\{^{1}H\}$ NMR spectrum of **DMQCd** in $CDCl_3$.

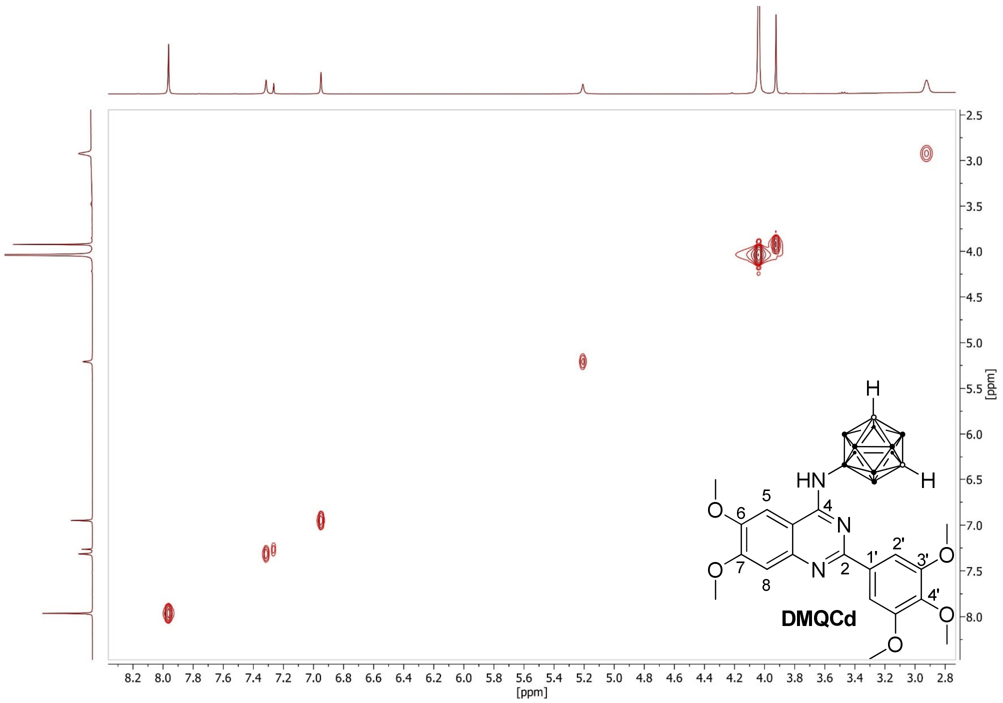

Figure S53. ^{1}H-COSY NMR spectrum of **DMQCd** in $CDCl_3$.

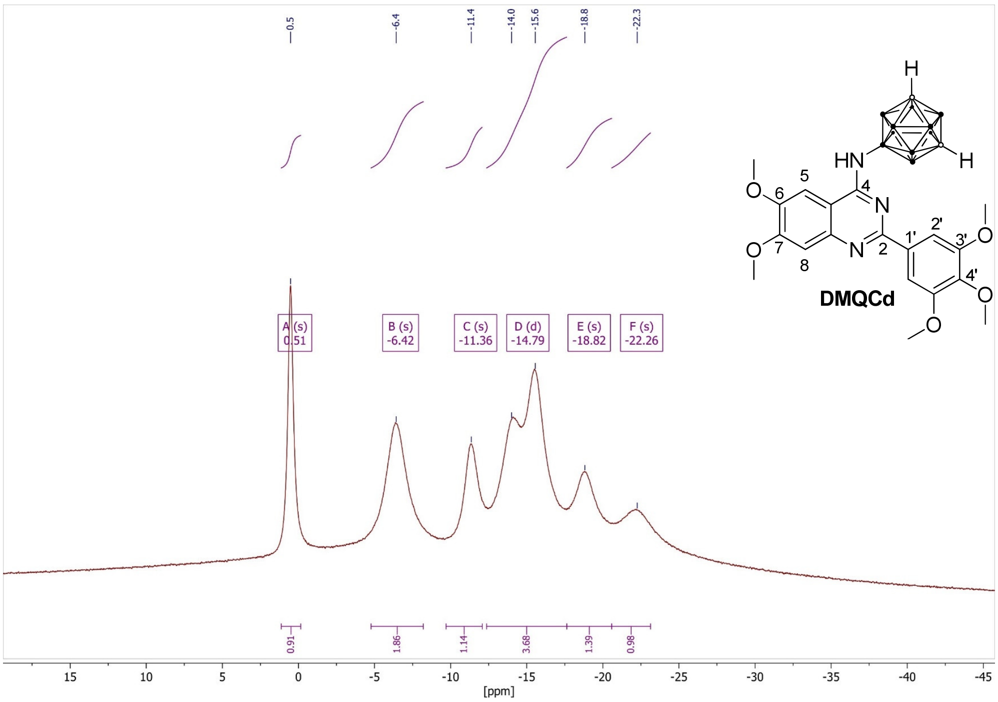

Figure S54. ${}^{11}B\{{}^{1}H\}$ NMR spectrum of **DMQCd** in $CDCl_3$.

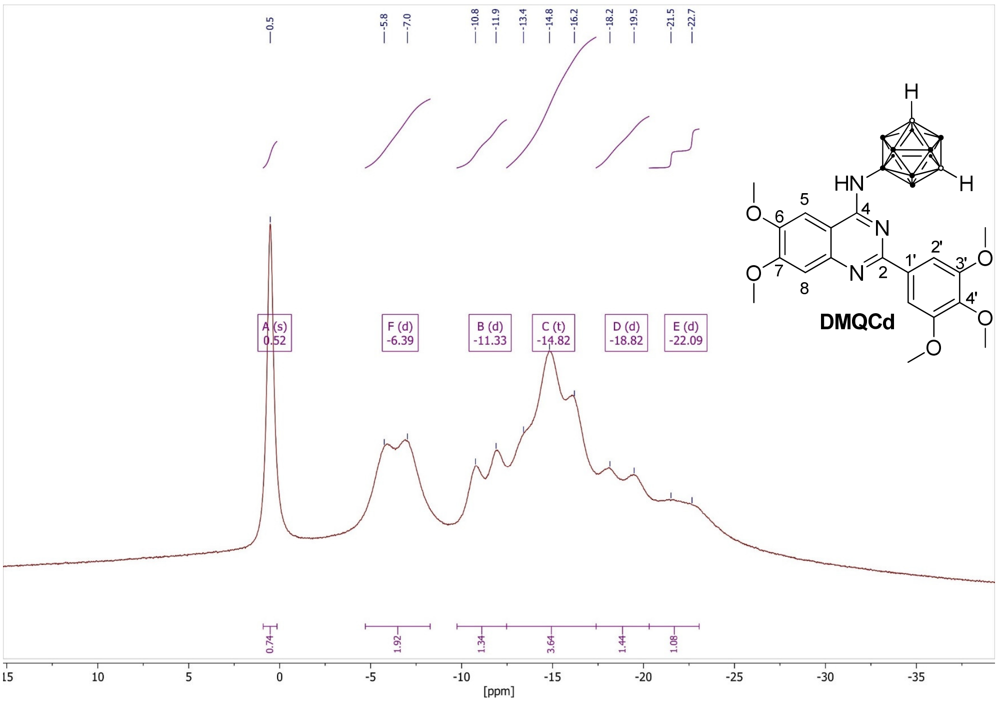

Figure S55. ${}^{11}B$ NMR spectrum of **DMQCd** in $CDCl_3$.

2. Biological Data

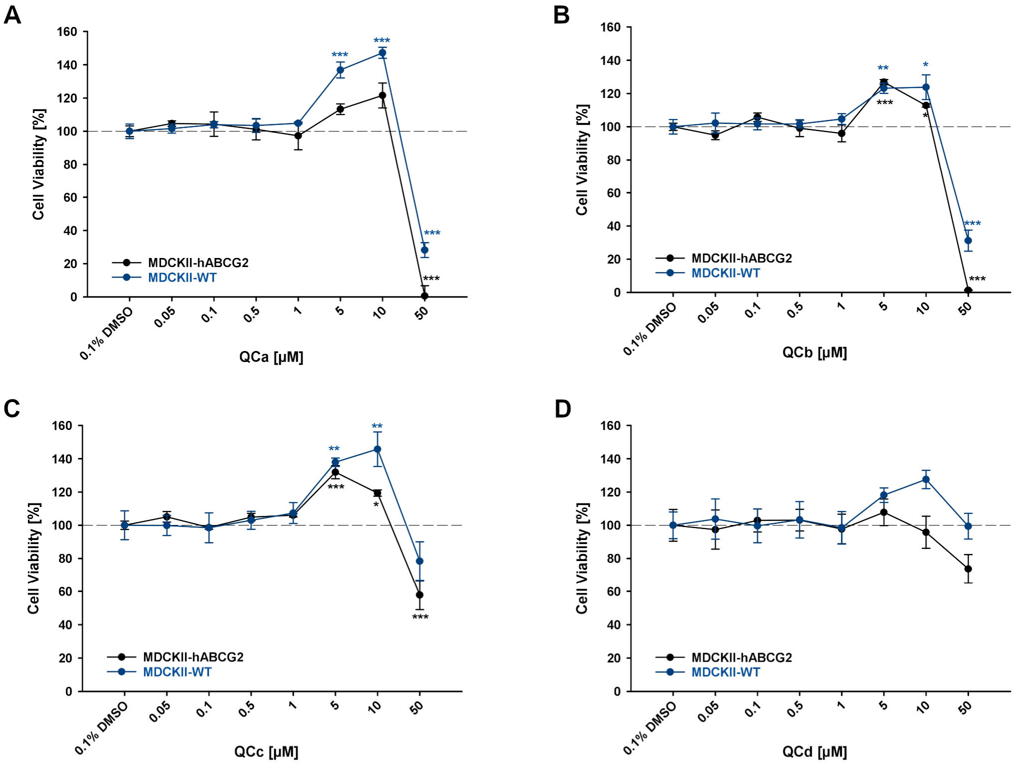

Figure S56. Cytotoxicity of quinazoline derivatives (A) **QCa**, (B) **QCb**, (C) **QCc**, and (D) **QCd**. MDCKII-hABCG2 and MDCKII-WT cells were incubated with selected compounds in increasing concentrations for 48 h. Afterwards, cell viability was assessed by water-soluble tetrazolium-1 (WST-1) assay. Data were normalized to solvent control (0.1% DMSO) and set as 100% (mean ± SEM, N = 3, one-way ANOVA with Holm-Šidák post hoc test, * significant difference in comparison to the solvent control. *** $p \leq 0.001$, ** $p \leq 0.01$, * $p \leq 0.05$).

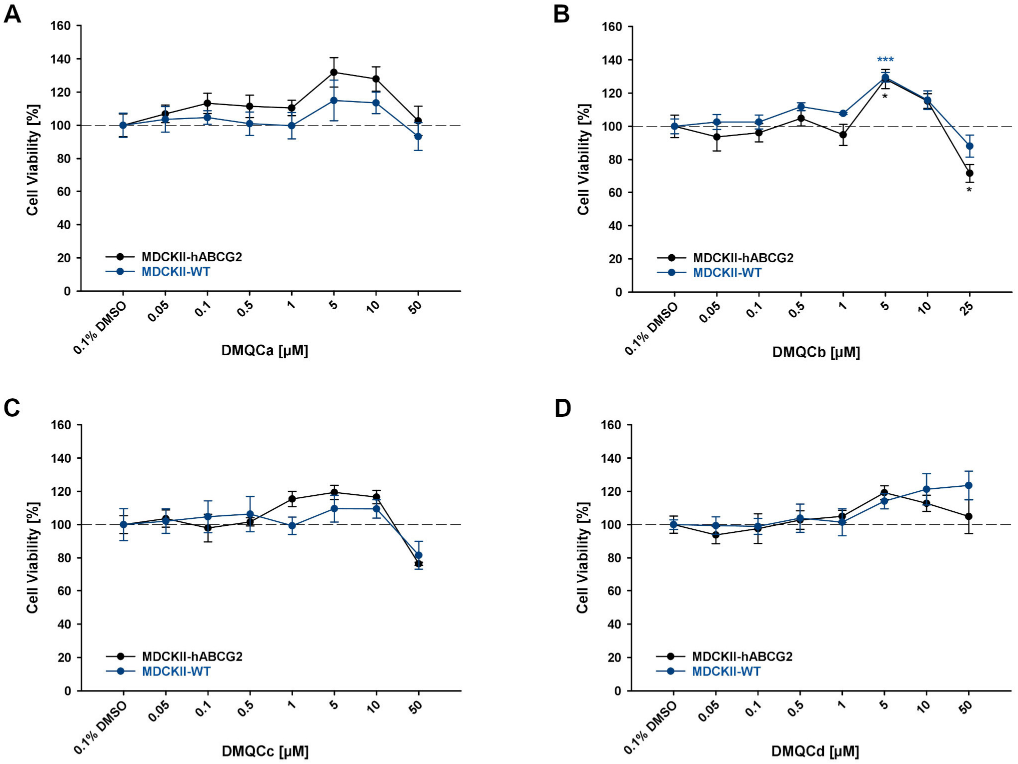

Figure S57. Cytotoxicity of dimethoxyquinazoline derivatives (A) **DMQCa**, (B) **DMQCb**, (C) **DMQCc,** and (D) **DMQCd**. MDCKII-hABCG2 and MDCKII-WT cells were incubated with dimethoxyquinazoline derivatives in increasing concentrations for 48 h. Afterwards, cell viability was assessed by water-soluble tetrazolium-1 (WST-1) assay. Data were normalized to solvent control (0.1% DMSO) and set as 100% (mean ± SEM, N = 3, one-way ANOVA with Holm-Šidák post hoc test, * significant difference in comparison to the solvent control. *** p ≤ 0.001, * p ≤ 0.05).

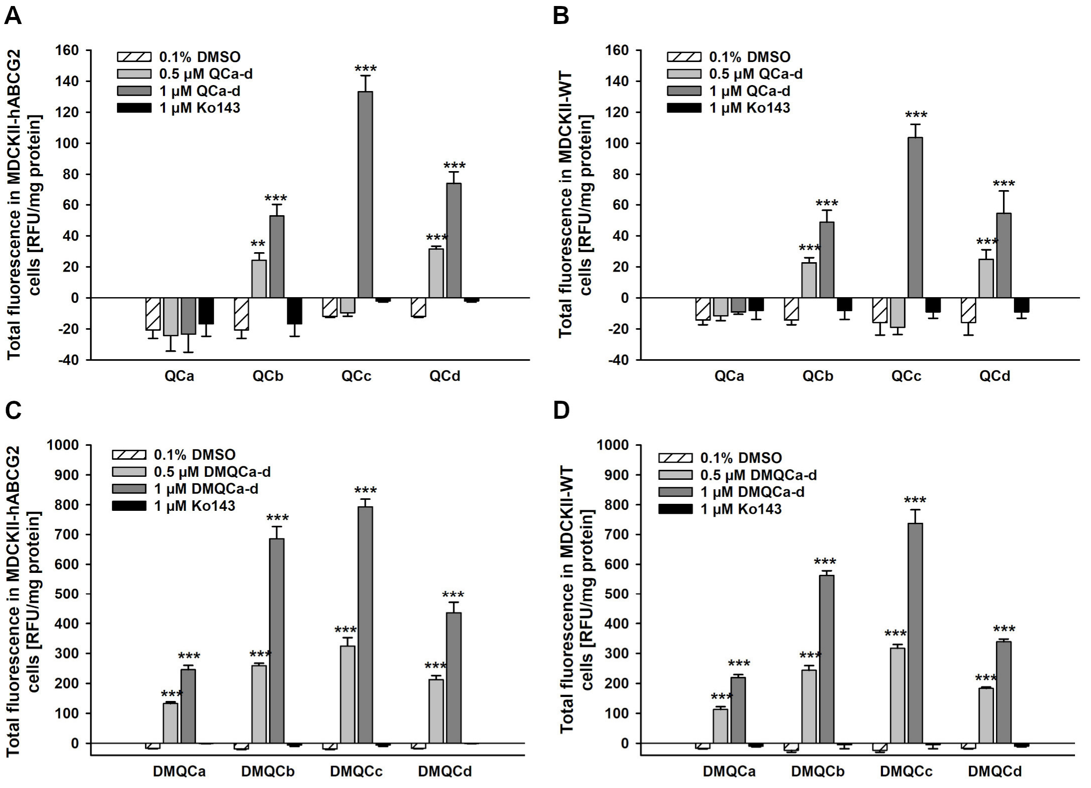

Figure S58. Autofluorescence of **QCa-d** in (A) MDCKII-hABCG2 and (B) MDCKII-WT cells, and **DMQCa-d** in (C) MDCKII-hABCG2 and (D) MDCKII-WT cells. Cells were incubated with **QCa-d** and **DMQCa-d** for 4 h and afterwards, cells were lyzed and intracellular fluorescence was determined as described (mean ± SEM, N = 3, one-way ANOVA with Holm-Šidák post hoc test, * significant difference in comparison to the solvent control. *** p ≤ 0.001, ** p ≤ 0.01,).

3. Molecular Docking

Table S1. Free binding energies of selected compounds towards human ABCG2 transporter in rigid ABCG2 protein (PDB code 5NJ3) and MXN-bound ABCG2 co-crystallized structure (MXN removed from crystal structure prior to docking; PDB code 6VXI).

Compound	Free binding energy [kcal/mol]	
	**5NJ3**	**6VXI**
**QCa**	−6.6	−5.6
**QCb**	−5.7	−5.1
**QCc**	−7.2	−7.0
**QCd**	−7.6	−5.9
**DMQCa**	−6.6	−6.1
**DMQCb**	−6.1	−6.2
**DMQCc**	−10.4	−9.8
**DMQCd**	−8.1	−8.3

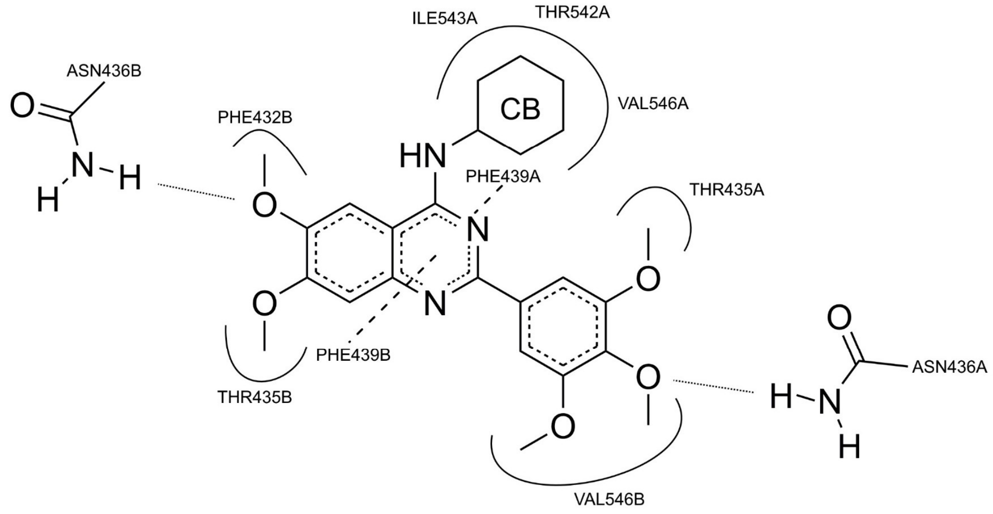

Figure S59. 2D interaction diagram of the top score pose of **DMQCd** within the inner cavity of the ABCG2 transporter protein (PDB code 5NJ3; CB equal to *meta*-carborane).
